# Supplementary material for: An evaluation of prescribing trends and patterns of claims within the Preferred Drugs Initiative in Ireland (2011–2016): an interrupted time-series study
Source: BMJ Open. 2018 Apr 20;8(4):e019315. doi: 10.1136/bmjopen-2017-019315 (PMC5914712; doi:10.1136/bmjopen-2017-019315)
Supplement: Supplementary file 1 [file bmjopen-2017-019315supp001.pdf]

Appendix

Table A1: Breakdown of PCRS reimbursed items: 2011-2016

Year	No. items issued	No. single-agent items issued across 7 drug classes*	% of items attributed to 7 drug classes*	No. items issued for preferred drugs	% preferred drug items within preferred drug classes	% preferred drug items across all prescriptions
**2011**	54,324,492	10,630,476	19.57%	2,520,986	23.71%	4.64%
**2012**	57,984,934	11,380,582	19.63%	2,641,897	23.21%	4.56%
**2013**	58,455,927	11,640,615	19.91%	2,708,855	23.27%	4.63%
**2014**	55,978,157	11,181,081	19.97%	2,655,422	23.75%	4.74%
**2015**	54,573,162	10,925,162	20.02%	2,610,926	23.90%	4.78%
**2016**	55,218,591	11,067,347	20.04%	2,627,631	23.74%	4.76%
**Total**	336,553,263	66,825,263	19.86%	15,765,717	23.59%	4.68%

***PPIs, Statins, ACEs, ARBs, SNRIs, SSRIs, Urology**

Table A2 Prevalence of PCRS reimbursed items by therapeutic drug class (single agent drugs)

	**PPIs**	**statins**	**ACEs**	**ARBs**	**SNRIs**	**SSRIs**	**urology**	**Other**	**Total**
**Year**									
**2011**	2,860,986<br>(5.27%)	3,286,352<br>(6.05%)	1,586,992<br>(2.92%)	849,807<br>(1.56%)	470,234<br>(0.87%)	1,247,643<br>(2.30%)	328,462<br>(0.60%)	43,694,016<br>(80.43%)	54,324,492<br>(100%)
**2012**	3,114,214<br>(5.37%)	3,501,257<br>(6.04%)	1,616,612<br>(2.79%)	899,594<br>(1.55%)	537,800<br>(0.93%)	1,355,921<br>(2.34%)	355,184<br>(0.61%)	46,604,352<br>(80.37%)	57,984,934<br>(100%)
**2013**	3,203,104<br>(5.48%)	3,582,112<br>(6.13%)	1,595,582<br>(2.73%)	920,851<br>(1.58%)	566,951<br>(0.97%)	1,404,466<br>(2.40%)	367,549<br>(0.63%)	46,815,312<br>(80.09%)	58,455,927<br>(100%)
**2014**	3,180,702<br>(5.68%)	3,339,227<br>(5.97%)	1,449,173<br>(2.59%)	867,567<br>(1.55%)	567,859<br>(1.01%)	1,399,724<br>(2.50%)	376,829<br>(0.67%)	44,797,076<br>(80.03%)	55,978,157<br>(100%)
**2015**	3,241,661<br>(5.94%)	3,129,117<br>(5.73%)	1,312,155<br>(2.40%)	816,250<br>(1.50%)	588,689<br>(1.08%)	1,441,270<br>(2.64%)	396,020<br>(0.73%)	43,648,000<br>(79.98%)	54,573,162<br>(100%)
**2016**	3,338,615<br>(6.05%)	3,106,569<br>(5.63%)	1,276,492<br>(2.31%)	817,135<br>(1.48%)	613,774<br>(1.11%)	1,499,543<br>(2.72%)	415,219<br>(0.75%)	44,151,244<br>(79.96%)	55,218,591<br>(100%)
**Total**	18,939,282<br>(5.63%)	19,944,634<br>(5.93%)	8,837,006<br>(2.63%)	5,171,204<br>(1.54%)	3,345,307<br>(0.99%)	8,348,567<br>(2.48%)	2,239,263<br>(0.67%)	269,710,000<br>(80.14%)	336,535,263<br>(100%)

Fig A1: Breakdown of PCRS reimbursed items by preferred drug status: 2011-2016

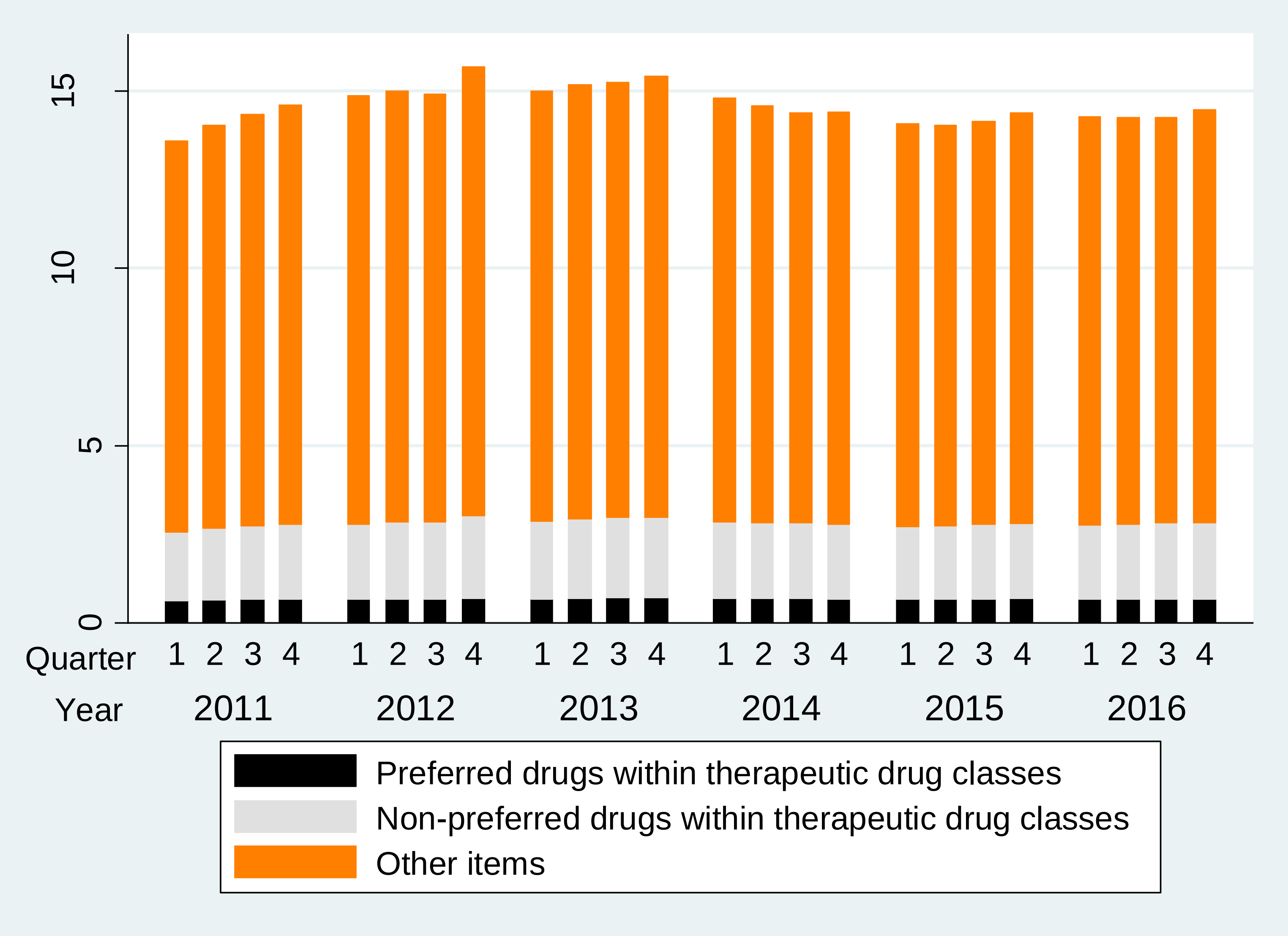

Fig A2: Observed percentage of preferred drugs by drug class

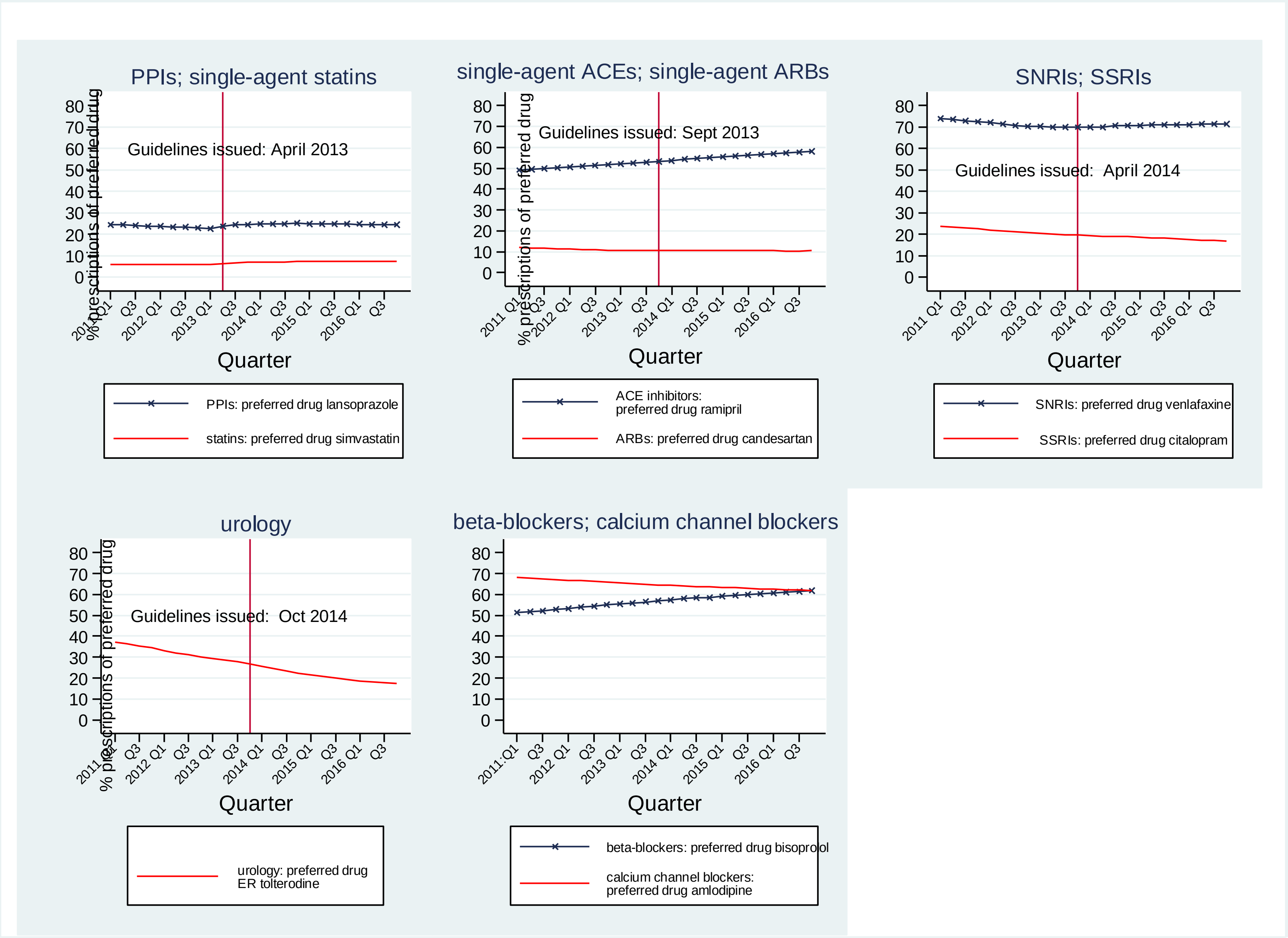

Table A3: Sensitivity analyses for segmented regression models

	Calendar quarters retained for analysis (Study Period)			
	**All available data: 9 quarters before guidelines, 15 quarters after guidelines, (Jan 11- Dec 16)**	**9 quarters before guidelines, 13 quarters after guidelines (Jan 11-Jun 16)**	**9 quarters before guidelines, 11 quarters after guidelines (Jan 11-Dec 15)**	**9 quarters before guidelines, 9 quarters after guidelines (Jan 11-Jun 15)**
**lansoprazole**				
Percentage of preferred drug items: beginning of study period (SE), 95% CI	24.53 (0.47), (23.59,25.47)	24.51 (0.40), (23.66,25.36)	24.47 (0.29), (23.85,25.09)	24.42 (0.19), (24.02,24.83)
Increase in % of preferred drug items per quarter following commencement of study period (SE), 95%CI, p-value	-0.21 (0.05), (-0.32,-0.11), p=0.001	-0.21 (0.05), (-0.32,-0.10), p=0.001	-0.21 (0.04), (-0.30,-0.11), p<0.001	-0.21 (0.04), (-0.28,-0.14), p<0.001
Increase in % of preferred drug items calendar quarter following PDI guidelines, (SE), 95%CI, p-value	1.21 (0.18), (0.84,1.57), p<0.001	1.21 (0.18), (0.83,1.59), p<0.001	1.22 (0.17), (0.85,1.59), p<0.001	1.26 (0.16), (0.90,1.61), p<0.001
Increase in % of preferred drug items per quarter post PDI guidelines, (SE), 95%CI, p-value	0.04 (0.04), (-0.03,0.12), p=0.25	0.06 (0.04), (-0.02,0.14), p=0.14	0.09 (0.04) (0.02,0.18), p=0.01	0.14 (0.03) (0.08,0.22), p<0.001
**simvastatin**				
Percentage of preferred drug items: beginning of study period (SE), 95% CI	5.94 (0.21), (5.50,6.38)	5.92 (0.17), (5.56,6.27)	5.89 (0.12), (5.63,6.15)	5.87 (0.07), (5.73,6.01)
Increase in % of preferred drug items per quarter following commencement of study period (SE), 95%CI, p-value	0.02 (0.03), (-0.04,0.07), p=0.54	0.02 (0.03), (-0.03,0.07), p=0.49	0.02 (0.02), (-0.03,0.07), p=0.32	0.02 (0.02), (-0.02,0.04), p=0.27
Increase in % of preferred drug items calendar quarter following PDI guidelines, (SE), 95%CI, p-value	0.30 (0.10), (0.10,0.50), p=0.01	0.30 (0.10), (0.10,0.51), p=0.01	0.32 (0.10), (0.12,0.52), p=0.01	0.42 (0.09), (0.23,0.62), p<0.001
Increase in % of preferred drug items per quarter post PDI guidelines, (SE), 95%CI, p-value	0.07 (0.02), (0.03,0.10), p=0.002	0.08 (0.02), (0.04,0.12), p=0.001	0.09 (0.02), (0.06,0.13), p<0.001	0.10 (0.01), (0.07,0.13), p<0.001

Table A3 (cont): sensitivity analyses for segmented regression models

	Calendar quarters retained for analysis (Study Period)					
	**All available data: 11 quarters before guidelines, 13 quarters after guidelines (Jan 11-Dec 16)**	**11 quarters before guidelines, 11 quarters after guidelines (Jan 11-Jun 16)**	**11 quarters before guidelines, 9 quarters after guidelines (Jan 11-Dec 15)**	**9 quarters before guidelines, 13 quarters after guidelines (Jul 11-Dec 16)**	**9 quarters before guidelines, 11 quarters after guidelines (Jul 11-Jun 16)**	**9 quarters before guidelines, 9 quarters after guidelines (Jul 11-Jan 15)**
**ramipril**						
Percentage of preferred drug items: beginning of study period (SE), 95% CI	49.14 (0.07), (48.99,49.28)	49.14 (0.08), (48.97,49.30)	49.14 (0.07), (48.98,49.28)	49.93 (0.07), (49.78,50.08)	49.92 (0.07), (49.76,50.10)	49.94 (0.07), (49.78,50.10)
Increase in % of preferred drug items per quarter following commencement of study period (SE), 95%CI, p-value	0.38 (0.01), (0.35,0.40), p<0.001	0.38 (0.02), (0.35,0.40), p<0.001	0.38 (0.02). (0.35,0.40), p<0.001	0.37 (0.01), (0.34,0.40), p<0.001	0.37 (0.01). (0.34,0.40), p<0.001	0.37 (0.01). (0.33,0.40), p<0.001
Increase in % of preferred drug items calendar quarter following PDI guidelines, (SE), 95%CI, p-value	0.16 (0.07), (0.01,0.31), p=0.04	0.16 (0.07), (0.01,0.32), p=0.04	0.16 (0.08), (-0.01,0.33), p=0.04	0.16 (0.07), (0.02,0.34), p=0.03	0.17 (0.08), (0.01,0.35), p=0.04	0.18 (0.09), (-0.01,0.35), p=0.06
Increase in % of preferred drug items per quarter post PDI guidelines, (SE), 95%CI, p-value	0.41 (0.01), (0.39,0.42), p<0.001	0.40 (0.01), (0.38,0.43), p<0.001	0.41 (0.01), (0.38,0.44), p<0.001	0.41 (0.01), (0.39,0.42), p<0.001	0.40 (0.01), (0.38,0.43), p<0.001	0.41 (0.01), (0.38,0.44), p<0.001
**candesartan**						
Percentage of preferred drug items: beginning of study period (SE), 95% CI	11.90 (0.08), (11.73,12.07)	11.90 (0.09), (11.71,12.09)	11.89 (0.08), (11.73,12.06)	11.60 (0.09), (11.42,11.80)	11.61 (0.11), (11.40,11.84)	11.60 (0.08), (11.41,11.78)
Increase in % of preferred drug items per quarter following commencement of study period (SE), 95%CI, p-value	-0.15 (0.01), (-0.17,-0.12), p<0.001	-0.15 (0.01), (-0.17,-0.12), p<0.001	-0.16 (0.01), (-0.17,-0.12), p<0.001	-0.15 (0.01), (-0.17,-0.12), p<0.001	-0.15 (0.01), (-0.18,-0.12), p<0.001	-0.15 (0.01), (-0.18,-0.12), p<0.001
Increase in % of preferred drug items calendar quarter following PDI guidelines, (SE), 95%CI, p-value	0.15 (0.06), (0.02,0.29), p=0.03	0.14 (0.06), (0.01,0.29), p=0.04	0.16 (0.06), (0.02,0.30), p=0.03	0.14 (0.07), (0.01,0.28), p=0.04	0.14 (0.07), (-0.01,0.28), p=0.06	0.15 (0.06), (0.01,0.29), p=0.05
Increase in % of preferred drug items per quarter post PDI guidelines, (SE), 95%CI, p-value	0.01 (0.01), (-0.01,0.03), p=0.46	0.01 (0.01), (-0.02,0.03), p=0.75	0.02 (0.01), (-0.01,0.05), p=0.25	0.01 (0.01), (-0.01,0.03), p=0.49	0.01 (0.01), (-0.03,0.03), p=0.82	0.01 (0.01), (-0.01,0.05), p=0.31

Table A3 (cont): sensitivity analyses for segmented regression models

	Calendar quarters retained for analysis (Study Period)					
	**All available data: 13 quarters before guidelines, 11 quarters after guidelines (Jan 11-Dec 16)**	**13 quarters before guidelines, 9 quarters after guidelines (Jan 11-Jun 16)**	**11 quarters before guidelines, 11 quarters after guidelines (Jul 11-Dec 16)**	**11 quarters before guidelines, 9 quarters after guidelines (Jul 11-Jun 16)**	**9 quarters before guidelines, 11 quarters after guidelines (Jan 12-Dec 16)**	**9 quarters before guidelines, 9 quarters after guidelines (Jan 12-Jun 16)**
**venlafaxine**						
Percentage of preferred drug items: beginning of study period (SE), 95% CI	73.61 (0.44), (72.69,74.53)	73.61 (0.46), (72.63,74.60)	72.56 (0.35), (71.81,73.31)	72.57(0.38), (71.75,73.40)	71.45 (0.22), (70.98,71.91)	71.46 (0.24), (70.94,71.98)
Increase in % of preferred drug items per quarter following commencement of study period (SE), 95%CI, p-value	-0.35 (0.05), (-0.46,-0.24), p<0.001	-0.35 (0.05), (-0.46,-0.24), p<0.001	-0.32 (0.05), (-0.43,-0.21), p<0.001	-0.32 (0.05), (-0.43,-0.20), p<0.001	-0.25 (0.04), (-0.34,-0.16), p<0.001	-0.25 (0.05), (-0.35,-0.14), p<0.001
Increase in % of preferred drug items calendar quarter following PDI guidelines, (SE), 95%CI, p-value	0.71 (0.27), (0.15,1.27), p=0.02	0.71 (0.28), (0.12,1.29), p=0.02	0.71 (0.28), (0.12,1.31), p=0.02	0.70 (0.29), (0.08,1.32), p=0.03	0.79 (0.29), (0.16,1.42), p=0.02	0.78 (0.31), (0.10,1.45), p=0.03
Increase in % of preferred drug items per quarter post PDI guidelines, (SE), 95%CI, p-value	0.26 (0.13), (-0.02,0.55), p=0.07	0.26 (0.14), (-0.04,0.55), p=0.08	0.27 (0.13), (-0.01,0.56), p=0.05	0.26 (0.14), (-0.02,0.57), p=0.08	0.26 (0.12), (0.01,0.52), p=0.04	0.26 (0.13), (-0.02,0.53), p=0.07
Increase in % of preferred drug April-June 2015 following introduction of generic duloxetine, (SE), 95%CI, p-value	-0.09 (0.30), (-0.73,0.54), p=0.76	-0.09 (0.31), (-0.76,0.57), p=0.77	-0.11 (0.32), (-0.79,0.57), p=0.74	-0.11 (0.33), (-0.82,0.60), p=0.75	-0.15 (0.35), (-0.89,0.60), p=0.68	-0.17 (0.37), (-0.97,0.64), p=0.66
Increase in % of preferred drug items per quarter post June 2015 , (SE), 95%CI, p-value	-0.08 (0.09), (-0.10,0.26), p=0.34	0.14 (0.12), (-0.10,0.39), p=0.24	-0.08 (0.08), (-0.09,0.26), p=0.34	0.14 (0.11), (-0.10,0.39), p=0.26	0.07 (0.06), (-0.06,0.20), p=0.27	0.10 (0.09), (-0.10,0.33), p=0.27
**citalopram**						
Percentage of preferred drug items: beginning of study period (SE), 95% CI	23.58 (0.13), (23.31,23.85)	23.58 (0.12), (23.32,23.83)	22.88 (0.14), (22.89,23.17)	22.87 (0.13), (22.59,23.14)	21.95 (0.08), (21.78,22.12)	21.93 (0.06), (21.81,22.04)
Increase in % of preferred drug items per quarter following commencement of study period (SE), 95%CI, p-value	-0.36 (0.01), (-0.39,-0.33), p<0.001	-0.36 (0.01), (-0.39,-0.33), p<0.001	-0.36 (0.02), (-0.40,-0.32), p<0.001	-0.36 (0.02), (-0.40,-0.33), p<0.001	-0.33 (0.01), (-0.36,-0.30), p<0.001	-0.33 (0.01), (-0.36,-0.31), p<0.001
Increase in % of preferred drug items calendar quarter following PDI guidelines, (SE), 95%CI, p-value	0.30 (0.08), (0.12,0.47), p=0.002	0.30 (0.08), (0.12,0.48), p=0.003	0.30 (0.09), (0.11,0.48), p=0.003	0.30 (0.09), (0.11,0.50), p=0.005	0.30 (0.08), (0.13,0.47), p=0.002	0.34 (0.08), (0.17,0.51), p=0.001
Increase in % of preferred drug items per quarter post PDI guidelines, (SE), 95%CI, p-value	-0.23 (0.02), (-0.27,-0.19), p<0.001	-0.22 (0.02), (-0.26,-0.18), p<0.001	-0.23 (0.02), (-0.27,-0.19), p<0.001	-0.22 (0.02), (-0.27,-0.17), p<0.001	-0.24 (0.02), (-0.26,-0.21), p<0.001	-0.23 (0.01), (-0.25,-0.20), p<0.001

Table A3 (cont): sensitivity analyses for segmented regression models

	Calendar quarters retained for analysis (Study Period)			
	**All available data: 15 quarters before guidelines, 9 quarters after guidelines (Jan 11-Dec 16)**	**13 quarters before guidelines, 9 quarters after guidelines (Jul 11-Dec 16)**	**11 quarters before guidelines, 9 quarters after guidelines (Jan 12-Dec 16)**	**9 quarters before guidelines, 9 quarters after guidelines (Jul 12-Dec 16)**
**ER tolterodine**				
Percentage of preferred drug items: beginning of study period (SE), 95% CI	37.27 (0.27), (36.69,37.84)	35.45 (0.30), (34.81,36.09)	33.16 (0.33), (32.46,33.87)	31.10 (0.39) (30.29,31.93)
Increase in % of preferred drug items per quarter following commencement of study period (SE), 95%CI, p-value	-1.00 (0.05), (-1.11,-0.88), p<0.001	-1.04 (0.07), (-1.21,-0.88), p<0.001	-0.97 (0.11), (-1.21,-0.73), p<0.001	-0.98 (0.06), (-1.11,-0.86), p<0.001
Increase in % of preferred drug items Jan-Mar 2013 following licensing of mirabegron, (SE), 95%CI, p-value	0.16 (0.24), (-0.35,0.66), p=0.52	0.21 (0.26), (-0.34,0.75), p=0.43	0.11 (0.25), (-0.44,0.65), p=0.68	*
Increase in % of preferred drug items per quarter post March 2013 (SE), 95%CI, p-value	-1.04 (0.06), (-1.17,-0.91), p<0.001	-1.03 (0.07), (-1.17,-0.89), p<0.001	-1.03 (0.06), (-1.17,-0.89), p<0.001	*
Increase in % of preferred drug items calendar quarter following PDI guidelines, (SE), 95%CI, p-value	-0.06 (0.24), (-0.57,0.45), p=0.82	-0.05 (0.25), (-0.57,0.49), p=0.86	-0.01 (0.24), (-0.51,0.49), p=0.96	-0.05 (0.22), (-0.52,0.43), p=0.86
Increase in % of preferred drug items per quarter post PDI guidelines, (SE), 95%CI, p-value	-0.63 (0.09), (-0.73,-0.52), p<0.001	-0.63 (0.06), (-0.73,-0.51), p<0.001	-0.62 (0.05), (-0.73,-0.51), p<0.001	-0.63 (0.06), (-0.75,-0.50), p<0.001

*omitted due to close proximity of study period (July 2012) and licensing of mirabegron (Jan 2013)

Table A4: Sensitivity analyses: alternative definition of calendar quarters for ACE inhibitors/ARBs

	**Calendar quarters: Jan-Mar, Apr-Jun, Jul-Sep, Oct-Dec (24 calendar quarters: Jan 11-Dec 16)**	**Calendar quarters: Mar-May, Jun-Aug, Sep-Nov, Dec-Feb (23 calendar quarters: Mar 11-Nov 16)**
**ramipril**		
Percentage of preferred drug items: beginning of study period (SE), 95% CI	49.14 (0.07), (48.99,49.28)	49.42 (0.07), (49.27, 49.58)
Increase in % of preferred drug items per quarter following commencement of study period (SE), 95%CI, p-value	0.38 (0.01), (0.35,0.40), p<0.001	0.37 (0.01), (0.34,0.40), p<0.001
Increase in % of preferred drug items calendar quarter following PDI guidelines, (SE), 95%CI, p-value	0.16 (0.07), (0.01,0.31), p=0.04	0.14 (0.08), (-0.01,0.32), p=0.17
Increase in % of preferred drug items per quarter post PDI guidelines, (SE), 95%CI, p-value	0.41 (0.01), (0.39,0.42), p<0.001	0.41 (0.01), (0.39,0.43), p<0.001
**candesartan**		
Percentage of preferred drug items: beginning of study period (SE), 95% CI	11.90 (0.08), (11.73,12.07)	11.78 (0.07), (11.63,11.92)
Increase in % of preferred drug items per quarter following commencement of study period (SE), 95%CI, p-value	-0.15 (0.01), (-0.17,-0.12), p<0.001	-0.15 (0.01), (-0.17,-0.13), p<0.001
Increase in % of preferred drug items calendar quarter following PDI guidelines, (SE), 95%CI, p-value	0.15 (0.06), (0.02,0.29), p=0.03	0.17 (0.06), (0.06,0.29), p=0.01
Increase in % of preferred drug items per quarter post PDI guidelines, (SE), 95%CI, p-value	0.01 (0.01), (-0.01,0.03), p=0.46	0.01 (0.01), (-0.02,0.02), p=0.90
