# Supplementary material for: An evaluation of prescribing trends and patterns of claims within the Preferred Drugs Initiative in Ireland (2011–2016): an interrupted time-series study
Source: BMJ Open. 2018 Apr 20;8(4):e019315. doi: 10.1136/bmjopen-2017-019315 (PMC5914712; doi:10.1136/bmjopen-2017-019315)
Supplement: Author's manuscript [file bmjopen-2017-019315.draft_revisions.pdf]

BMJ Open is committed to open peer review. As part of this commitment we make the peer review history of every article we publish publicly available.

When an article is published we post the peer reviewers' comments and the authors' responses online. We also post the versions of the paper that were used during peer review. These are the versions that the peer review comments apply to.

The versions of the paper that follow are the versions that were submitted during the peer review process. They are not the versions of record or the final published versions. They should not be cited or distributed as the published version of this manuscript.

BMJ Open is an open access journal and the full, final, typeset and author-corrected version of record of the manuscript is available on our site with no access controls, subscription charges or pay-per-view fees (http://bmjopen.bmj.com).

If you have any questions on BMJ Open's open peer review process please email info.bmjopen@bmj.com

BMJ Open

An evaluation of prescribing trends and patterns of claims within the Preferred Drugs Initiative in Ireland: 2011-2016

Journal:	*BMJ Open*
Manuscript ID	bmjopen-2017-019315
Article Type:	Research
Date Submitted by the Author:	25-Aug-2017
Complete List of Authors:	McDowell, Ronald; Royal College of Surgeons in Ireland, HRB Centre for Primary Care Research<br>Bennett, Kathleen; Royal College of Surgeons in Ireland, Division of Population Health Sciences<br>Moriarty, Frank; Royal College of Surgeons in Ireland, HRB Centre for Primary Care Research, Department of General Practice<br>Clarke, Sarah; Health Service Executive Medicines Management Programme<br>Barry, Michael; National Centre for Pharmacoeconomics, Trinity Centre for Health Sciences<br>Fahey, Tom; Royal College of Surgeons in Ireland, Department of General Practice
**Primary Subject Heading**:	Epidemiology
Secondary Subject Heading:	General practice / Family practice, Health policy, Public health
Keywords:	Ireland, physician prescribing patterns, interrupted time series

Note: The following files were submitted by the author for peer review, but cannot be converted to PDF. You must view these files (e.g. movies) online.

Fig1a.gph
Fig1cont.gph
Fig 2.gph

SCHOLARONE™ Manuscripts

An evaluation of prescribing trends and patterns of claims within the Preferred Drugs Initiative in Ireland: 2011-2016

McDowell RD[1], Bennett K[2], Moriarty F[1], Clarke S[3], Barry M[4], Fahey T[1]

[1] HRB Centre for Primary Care Research,
Department of General Practice,
Royal College of Surgeons in Ireland Medical School,
123 St Stephens Green,
Dublin 2

[2] Division of Population Health Sciences,
Royal College of Surgeons in Ireland,
Beaux Lane House,
Mercer Street Lower,
Dublin 2

[3] Health Service Executive Medicines Management Programme,
Trinity Centre for Health Sciences,
St. James's Hospital,
Dublin 8

[4] National Centre for Pharmacoeconomics,
Trinity Centre for Health Sciences,
St. James's Hospital,
Dublin 8

Correspondence to:

Ronald McDowell
Email: ronaldmcdowell@rcsi.ie

Word count: 3,413

Keywords: Ireland, physician prescribing patterns, interrupted time series

For peer review only

Abstract

Objective: To examine the impact of the Preferred Drugs Initiative (PDI), an Irish health policy aimed at reducing the cost of prescription medicines.

Design: Retrospective repeated cross-sectional study spanning the years 2011 to 2016.

Setting: Health Service Executive Primary Care Reimbursement Scheme pharmacy claims data for General Medical Services (GMS) patients, approximately 40% of the Irish population.

Participants: Adults aged ≥18 years between 2011 to 2016 eligible for the GMS scheme.

Primary and secondary outcome measures: The proportion of PDI medications within each drug class per calendar quarter. Logistic segmented regression analysis was used to model prescribing of the preferred drug within each medication group and to assess the impact of clinical practice guidelines. Savings in drug expenditure with changes in PDI drugs were estimated.

Results: Between 2011 and 2016 around one quarter (23.59%) of all medications were for single-agent drugs licensed in the seven therapeutic drug classes. There was a small increase in the proportion of PDI drugs, increasing from 4.64% of all medications in 2011 to 4.76% in 2016 (p<0.001). The proportion of preferred drugs within each drug class was significantly higher immediately following publication of the guidelines for all classes bar urology medications, with the largest effects noted for venlafaxine (OR 1.08, 95%CI (1.07,1.09), p<0.001), lansoprazole (OR 1.12, 95%CI (1.11,1.12), p<0.001) and simvastatin (OR 1.12, 95%CI (1.11,1.13), p<0.001). For four medicine groups prescribing of the preferred drug continued to increase until the end of 2016, although the increases per calendar quarter were smaller. The estimated cost savings between 2013 and 2016 was €3million.

Conclusion: There has been considerable variation between medicine groups in relation to the impact of PDI prescribing guidelines, with modest changes in prescribing observed in most classes. More intensive implementation is needed before the PDI scheme delivers the anticipated €15million per year cost saving.

For peer review only - http://bmjopen.bmj.com/site/about/guidelines.xhtml

Strengths and limitations of this study

- PCRS data covers pharmacy claims for prescriptions issued to General Medical Scheme (GMS) patients (around 38% of the Irish population)
- Methods used are appropriate given lack of a control group and the phased introduction of the preferred drug guidelines
- GMS patients are weighted towards older adults and those in receipt of social welfare
- Results based on aggregated data give an overview of the Preferred Drugs Initiative in its early years but require further detailed analysis to examine prescriber and patient heterogeneity

For peer review only

For peer review only - http://bmjopen.bmj.com/site/about/guidelines.xhtml

Background

The Health Service Executive (HSE) in Ireland spent €1.05 billion in 2015 reimbursing pharmacists for the cost of prescription items issued to General Medical Service (GMS) patients via the Primary Care Reimbursement Scheme (PCRS).(1) This is the largest community drug scheme in Ireland, providing access to free or minimal cost health care for eligible patients. Eligibility is based on criteria such as age or means testing. Historically Ireland has spent as much as 50% above the EU average per capita on drugs for a variety of reasons, such as low levels of use of generic medications and higher negotiated prices with pharmaceutical companies for both patented and generic drugs.(2, 3)

Against the background of an ageing population (4), the economic downturn of 2008 and rising drug costs the HSE established the Medicines Management Programme (MMP) in 2013. The MMP has undertaken a number of initiatives aimed at enhancing evidence-based and cost-effective prescribing (5), one of which is the Preferred Drugs Initiative (PDI). The PDI recommends a single ‘preferred drug’ within a therapeutic drug class as the prescriber’s drug of first choice. Factors considered when selecting the preferred drug include clinical efficacy, ease of administration, the possibility of side effects or interactions with other drugs, cost, and national and international clinical guidelines. Recommendations for preferred drugs are made on an ongoing basis, with the findings disseminated through the publication of prescribing guidelines and GP meetings. The issuing of preferred drugs is voluntary and no incentives are given to prescribers to issue the preferred drug instead of others from within the same therapeutic drug class. It is estimated that increased provision of the preferred drugs could save the HSE €15 million per year.(5)

As of September 2016 evaluation reports have been published for the first ten therapeutic drug classes covered by the Initiative.(6) These are Proton Pump Inhibitors (PPIs), statins, Angiotensin-Converting Enzyme (ACE) inhibitors, Angiotensin-II Receptor Blockers (ARBs), Serotonin Noradrenaline Reuptake Inhibitors (SNRI), Selective Serotonin Reuptake Inhibitors (SSRI), medications for treating urological conditions (urinary incontinence, frequency and overactive bladder), oral anticoagulants for stroke prevention in patients with non-valvular atrial fibrillation, beta-blockers and calcium channel blockers. The aims of this paper are to: (i) examine the trends and patterns of pharmacy claims for seven PDI drug classes among eligible adult GMS patients in Ireland between 2011 and 2016; (ii) assess the impact of the PDI recommendations over time using logistic segmented regression analysis; and (iii) estimate the cost savings due to the PDI during these years.

For peer review only

For peer review only - http://bmjopen.bmj.com/site/about/guidelines.xhtml

Methods

The STrengthening the Reporting of Observational Studies in Epidemiology (STROBE) guidelines were used in the reporting of this study (7).

Data

HSE-PCRS monthly pharmacy claims were analysed from 2011 to 2016. Analyses were limited to these years in order to avoid confounding changes in prescribing patterns associated with other pharmaceutical policy changes. The data includes all pharmacy claims made for GMS patients and for which the cost of the claim has been reimbursed to community pharmacies by the HSE.

Preferred Drugs Initiative

The first seven medication classes covered by the PDI are considered in this paper. The preferred drugs in each of these classes were lansoprazole(PPIs), simvastatin (statins), Ramipril (ACE inhibitors), candesartan (ARBs), venlafaxine (SNRIs), citalopram (SSRIs) and extended release (ER) tolterodine (urology medications). Guidelines for beta-blockers and calcium channel blockers were only introduced in September 2016. Prescribing of oral anticoagulants among patients with non-valvular atrial fibrillation was not examined as these patients could not be identified on the basis of their prescribing history alone; diagnostic codes are not available in PCRS data. Prescriptions issued to children (those under 18 years), hospital emergency items, out-of-hours prescriptions and items not considered medications and without an ATC code (such as medical devices and dressings) were excluded.

Analytical methods/approach

Descriptive statistics were used to summarise relevant medications from the HSE-PCRS database and the seven classes of PDI drugs. Only single-agent drugs are considered in this paper, as this is the primary focus of the PDI. Combination products containing the preferred drug do not always exist and the pricing structures for these products differ from single-agent medications.

Calendar quarters (January-March, April-June, July-September, October-December) for each year were used to aggregate the data in order to reduce the number of time-points included in the longitudinal analyses and because changes in prescribing patterns tend to be gradual rather than instantaneous. For each therapeutic drug class logistic segmented regression was used to estimate the proportion of the preferred drug per drug class per calendar quarter between 2011 to 2016, allowing for any changes that might have taken place following issuing of guidelines. This is a commonly-used strategy for analysing interrupted time series, considered to be the strongest, quasi-experimental approach for evaluating longitudinal effects of interventions where no control group is available.(8) The regression equations used had the form

$$\log\left(\frac{r_{ij}}{1-\left(\frac{r_{ij}}{n_{ij}}\right)}\right) = \left(\beta_{oj} + \beta_{1j}x_{ij1} + \beta_{2j}x_{ij2} + \beta_{3j}x_{ij3}\right) + e_{ij} \qquad (i = 0, \dots, 23)$$

where for each medicine group j $(j = 1, \dots, 7)$

For peer review only - http://bmjopen.bmj.com/site/about/guidelines.xhtml

r_{ij} is the number of items of the preferred drug reimbursed at time (quarter) i

n_{ij} is the total number of class-specific items reimbursed in the same quarter i

β_{oj} is the log-odds of items being preferred drugs at t=0 (Jan-Mar 2011),

β_{1j} is the log-odds of the estimated change in items being preferred drugs immediately following guidelines (the "change of level")

β_{2j} is the log-odds of the change in items being preferred drugs per calendar quarter (the "slope") before the guidelines

β_{3j} is the log-odds of the change in items being preferred drugs per calendar quarter (the "slope") post -guidelines

e_{ij} is the residual for calendar quarter i.

The x_{ijk} $(k = 1,2,3)$ were calculated from the data according to standard practice. (9)

By coincidence rather than design issuing of guidelines for each medicine group occurred at the beginning of the calendar quarters listed above, with the exception of the guidelines for ACEs and ARBs. Sensitivity analyses were used to explore whether the results varied when the calendar quarters were constructed differently (March-May, June-August, September-November, December-February) for these groups. Given that the time-periods prior to and post dissemination of the guidelines varied between medicine groups, sensitivity analyses were also used to examine whether results were dependent on the length of time considered before and after guidelines.

The models above were used to estimate increases or decreases in costs for each drug group associated with the PDI. The predicted number of preferred drug items from each class was compared with the number which would have been issued had the trend in prescribing estimated before the guidelines continued i.e. the estimates of $\beta_{oj}(\widehat{\beta_{oj}})$ and $\beta_{2j}(\widehat{\beta_{2j}})$ remained unchanged, $\widehat{\beta_{1j}}$ was constrained to be zero and the estimate of $\beta_{3j}(\widehat{\beta_{3j}})$ was set equal to $\widehat{\beta_{2j}}$. The difference in the number of preferred drug items under the two scenarios was multiplied by the average price of the preferred drug, calculated across all reimbursements between dissemination of the guidelines and the end of 2016. The difference in the number of non-preferred drug items was multiplied by a weighted average of the price of all other drugs from within the medicine class, weighted according to the overall distribution of these items between issuing of the guidelines and the end of December 2016. These two costs were combined to give an overall cost differential.

All analyses were conducted using Stata 14.0SE.(10) Results were held to be significant if they referred to statistical significance on a two-sided design-based test evaluated at the 0.05% level.

For peer review only - http://bmjopen.bmj.com/site/about/guidelines.xhtml

Results

Descriptive statistics

A total of 336,535,263 prescription items for medications were reimbursed by 4,465 PCRS prescribers for 1,919,681 GMS adults aged 18 years and over between 2011 and 2016. Approximately 55 million items were reimbursed per year, with the number of items peaking slightly in 2012 and 2013. During the six-year period 48.8 million (19.86%) prescription items were for the single-agent medicines licensed across the seven therapeutic drug classes considered. The drug classes most commonly prescribed to GMS patients were statins (5.93% of all items) and PPIs (5.63%), with the least common being SNRIs (0.99%) and drugs for treating urological conditions (0.67%). The descriptive statistics for each PDI medication class over the six-year period are outlined in Table 1.

The percentage of items relating to the seven drug classes increased slightly from 19.57% in 2011 to 20.04% in 2016, with small changes observed in the volume of prescriptions issued per each PDI medicine group over this time. More detailed breakdowns of PDI medicine groups per calendar year and quarter are given in Appendix Tables A1 & A2 and Figure A1.

Preferred Drugs Initiative

Within the seven PDI drug classes considered, 23.59% of all prescription items were for the named preferred drugs. However, there was considerable variation between PDI drug classes both in terms of ranking and percentage coverage of the preferred drug (see Table 1). The most commonly prescribed preferred drug within the relevant drug class was venlafaxine, which comprised 70.99% of all SNRI prescriptions. This was followed by ramipril (53.41% of all single-agent ACEs), ER tolterodine (25.79% of urology items), lansoprazole (24.14% of PPIs), citalopram (19.77% of SSRIs), candesartan (10.78% of all single-agent ARBs) and simvastatin (6.59% of all single-agent statins). The ranking of the preferred drugs within classes varied from first (ACE inhibitors and SNRIs), to second-last (statins). There was a small but statistically significant increase over time in the proportion of all medications which were for the PDI drugs, increasing from 4.64% in 2011 to 4.76% in 2016 ($p<0.001$).

Impact of clinical guidelines

Comparing prescribing patterns within each medication class in the three months pre-and post-publication of the PDI guidelines there was a modest increase in the proportion of preferred drugs in four drug classes (PPIs ($p<0.001$), statins ($p<0.001$), ACE inhibitors ($p<0.001$) and SNRIs ($p=0.08$)), little change in two other drugs classes (ARBs ($p=0.76$) and SSRIs ($p=0.37$)), and a large reduction in percentage terms in prescribing of the PDI agent ER tolterodine ($p<0.001$) (Table 1). Two preferred drugs, citalopram and ER tolterodine, were ranked lower within their respective classes between issuing of the guidelines and the end of 2016 than before. Figure 1 illustrates the secular trends for preferred drugs across the seven PDI categories by calendar quarters between 2011 and 2016: plots of the actual proportion of preferred drug items within each drug group between 2011 and 2016 are given in Appendix Figure A2.

Segmented logistic regression showed changes in the odds for all preferred drugs (Table 2). For three groups of medicines, there was significant evidence of modest increases in prescribing of the preferred drug in the quarter immediately after issuing of the guidelines (venlafaxine OR

For peer review only - http://bmjopen.bmj.com/site/about/guidelines.xhtml

1.08, 95%CI (1.07,1.09), p<0.001); lansoprazole (OR 1.12, 95%CI (1.11,1.12), p<0.001); simvastatin (OR 1.12, 95%CI (1.11,1.12), p<0.001). This corresponded to absolute increases of 1.67%, 1.72% and 0.56% respectively. For each of these three preferred drugs there was a small but continued increase in prescribing in subsequent quarters. For both candesartan and citalopram, for which prescribing within their PDI drug classes was in decline prior to the guidelines being issued, prescribing increased immediately following the PDI guidelines (candesartan OR 1.04, 95%CI (1.02,1.05), p<0.001; citalopram OR 1.03, 95%CI (1.02,1.04), p<0.001). However, declines in the prescribing of citalopram resumed in July 2014, although the decline was less steep than before the guidelines (p<0.001). For the other two medicine groups (ACEs and urology items), there was no notable impact and the secular trend observed before issuing of the guidelines continued afterwards: ramipril continued to increase in popularity as the ACE of choice and prescribing of ER tolterodine continued to decline. See Figure 2 for plots of the estimated proportion of preferred drug items within each therapeutic drug class between 2011 and 2016 according to the regression models.

Sensitivity analyses showed that the results were materially unaffected when the calendar quarters used for analyses of ACEs and ARBs varied or when the length of time studied before and after the guidelines was changed (Appendix Table A3). Consequently the results above span the entire study period of 2011-2016 and use the same calendar quarters (January-March, April-June, July-September, October-December) for each medicine group.

<u>Cost savings</u>

Overall, the cost savings after introduction of the PDI amounted to a total of €2,907,081 across all seven PDI drug classes (Table 2). The savings associated with changes in prescribing following issuing of guidelines for the seven drug classes were estimated to be €191k in 2013, €517k in 2014, €887k in 2015 and €1,312k in 2016. There were savings in each group except urology medications, even though changes in dispensed medications were often minimal. The greatest impact was on the amount spent on SNRIs, with an estimated saving of €1,377k between 2014-2016. This is due to the much higher cost of the non-preferred drug duloxetine to the preferred drug venlafaxine, such that small changes in prescribing translated into considerable savings. Other groups where the savings were marked were for the two larger volume groups where the guidelines had first been issued- PPIs saving €808k and statins saving €564k. For two medicine groups where prescribing of the preferred drug was in decline before guidelines were issued (ARBs and SSRIs), even the small short-term changes in prescribing translated into some savings. The smallest saving was in the prescribing of ramipril, despite being the most commonly prescribed ACE. No savings were observed for urology medications.

For peer review only - http://bmjopen.bmj.com/site/about/guidelines.xhtml

Discussion

Principal findings

The seven drug classes considered that form part of the PDI accounted for approximately 20% of all medications reimbursed by the PCRS between 2011 and 2016. Changes in prescribing observed over the study period varied by PDI drug class, with substantial differences in the ranking order and quantity of preferred drug prescribed. Overall, the impact of the PDI guidance was modest, with an inconsistent pattern observed across all therapeutic drug classes, and only a small increase (0.13%) in the proportion of preferred drugs issued overall between 2011 and 2016. Across the PDI drug classes some differences emerged: in the first group of PDI drugs there was a modest increase in prescribing of the preferred drug immediately following issuing of the guidelines and a continued though small increases subsequently (PPIs, Statins and SNRIs); in the second group of PDI drugs (SSRI and ARBs) there was a temporary increase in prescribing of the preferred drug just after the guidelines were issued; lastly, in the third group of PDI drugs (ACE and urology), there appeared to be little or no impact of clinical guidance. The reasons for such diversity are not known. ACE inhibitors are relatively inexpensive and this may account in part for the trend in ramipril prescribing remaining unaltered. Declines in the prescribing of ER tolterodine have been due to the increasing popularity of other more expensive non-preferred options such as mirabegron.

Context of other studies

PDI guidelines to date have been disseminated to prescribers mainly through correspondence and GP meetings. The literature shows that educational programmes and publication of guidelines in themselves tend to have little effect on influencing prescribing practice, and that these need to be enhanced with other strategies.(11) In a systematic review of 79 studies examining interventions which changed doctor prescribing behaviour, the most effective interventions were patient-mediated interventions, outreach (such as local opinion leaders and academic detailing), audit and feedback (including local consensus processes) and reminders.(12) A combined programme of education, therapeutic revelation of eligible patients and performance feedback resulted in savings when shifting physician prescribing to a preferred histamine-2-receptor antagonist.(13) In a study of changes in the use of losartan versus other single ARBs in Sweden investigators concluded that multiple and intensive demand-side measures are needed to change physician prescribing habits.(14) Other strategies which have been found to be helpful include direct involvement of the community pharmacist and repeated face-to-face engagement from those seeking to encourage change with the prescriber through academic detailing.(15) Technological advances, such as alerts and prompts when issuing a drug which is not the preferred drug may also prove useful.(16)

There are other options which may be considered more directive, such as reducing choice for either patient or prescriber. In Australia only four statins are licensed.(17) It has been suggested that because prescribers can develop expertise of only a certain number of drugs, more restrictive formularies may also provide benefits to quality of prescribing (18, 19). In Sweden, the introduction of the ‘Wise List’, an evidence-based formulary of essential medicines, increased adherence to guideline recommendations in primary care from 80% to 90% and reduced variation in prescribing(20). The vast majority of patients and doctors had positive attitudes towards the ‘Wise List’(21). The introduction of co-payments, where the patient has to pay the difference between the price of the preferred drug and their chosen alternative, has the potential to be a considerable driver of change. In a study of the effect of tiered prescription co-

For peer review only - http://bmjopen.bmj.com/site/about/guidelines.xhtml

payments on the use of preferred brand medications in the US, tiered prescription co-payments were associated with a significant shift from non-preferred to preferred brand medication.(22) While dramatic changes in co-payments may result in more patients switching to preferred agents (such as statins, ACE inhibitors and PPIs), they may also increase the risk of patients stopping their medication or becoming non-adherent (23, 24). However such a scheme may be difficult to develop for Irish GMS patients who are the least financially independent individuals. Co-payment may lead to accusations of a two-tier service between patients who cannot afford to choose their medication and those who can do so. Similarly, recent work has shown the drivers of drug expenditure in high income countries varies substantially, with several other factors aside from physician prescribing behavior and patient preference determining national drug expenditure.(25)

Strengths and limitations

There are a number of strengths to this study. Our prescription sample is large and generalisable: PCRS data covers the entire GMS population of Ireland (around 38% of individuals). Despite the guidelines being introduced incrementally, the results were invariant to the time periods studied pre- and post-publication of clinical guidelines. Although policy changes, such as the PDI, can be challenging to evaluate due to the lack of control or comparison group, this study utilised the most robust quasi-experimental approach available. However, there are limitations to the study. GMS patients are weighted towards older adults and those socially and financially disadvantaged and so the results may not be reflective of the entire population in receipt of prescription medication. There is no way of knowing whether prescribers approached existing patients with regards to changes in their medication and/or whether these approaches were successful. Patient-specific factors may mean that issuing of the preferred drug may not have been appropriate or possible. It is also feasible that non-preferred drugs have been initiated in secondary rather than primary care. Neither prescribers nor patients are homogeneous entities and considerable variation may exist within both; further research will examine the interaction between patient and practitioner effects.

Policy implications and future research

The Preferred Drug Initiative has been developed to encourage evidence-based, cost-effective prescribing, but in view of the limited changes to date it has delivered only a modest amount of potential cost savings in terms of the money spent on the prescription items. If efforts are to be enhanced, the energies need to focus on the larger volume medicine groups (PPIs and statins) where preferred drugs account for a small proportion of prescribing in that drug class, or medicine groups where there is considerable variation between the least and most expensive licensed medications (e.g. SNRIs). It may be challenging to encourage prescribers to switch to medicines which have been in decline in recent years. To enhance the impact of the PDI, multi-faceted interventions appear most likely to succeed. Financial incentives to prescribers may be one possible component of such interventions, as operated in Irish primary care for a time in the 1990's(26). They have been generally found to result in improvement in prescribing cost outcome (27), however any incentives for PDI drugs need to be aligned with professional values of prescriber, and be mindful of personal preferences of patients taking long-term medication (28-30). The effectiveness of such interventions is important to consider and although this has generally been evaluated using observational methods, experimental approaches may also be feasible. For example, the introduction of free, convenient access to an

For peer review only - http://bmjopen.bmj.com/site/about/guidelines.xhtml

evidence-based list of safe, effective and cost-effective medicines in Canadian primary care is currently being evaluated in the CLEAN Meds randomised controlled trial (30).

Conclusions

Since the introduction of the PDI in 2013, there have been some modest cost savings across the majority of PDI drug classes. However, more intensive implementation is needed before the PDI delivers the estimated €15million per year cost saving that was anticipated. Multifaceted interventions will be required to enhance the coverage and impact of the PDI so that these benefits can be realised.

For peer review only

For peer review only - http://bmjopen.bmj.com/site/about/guidelines.xhtml

Acknowledgements: Thanks to the Health Services Executive Primary Care Reimbursement Scheme (HSE-PCRS) for the use of pharmacy claims database.

Contributors: RMcD drafted and planned all aspects of study design, cleaned and prepared data for analysis, conducted the statistical analyses and conducted a preliminary overview of the literature. KB prepared the monthly PCRS claims downloads and gave significant methodological guidance on the analysis strategy. FM provided guidance on pharmaceutical matters and contributed to the discussion on context, policy implications and future research. SC and MB facilitated access to the claims data with the PCRS, gave detailed information on roll-out and implementation of the Preferred Drugs Initiative, and contributed to interpretation of the results within the wider context of prescribing in Ireland. All authors read and approved submission of the paper.

Funding: The primary author was funded by the Health Research Board (HRB) of Ireland under the HRB Centre for Primary Care Phase 2 Funding award, grant HRC/2014/1.

Competing interests: None declared.

Data-sharing statement: No additional data are available.

For peer review only

References

1. Service HSEPCR. Primary Care reimbursement Service Statistical Analysis of Claims and Payments 2015. Available from: http://www.hse.ie/eng/staff/PCRS/PCRS_Publications/PCRS-statistical-analysis-of-claims-and-payments-2015.pdf.
2. Children HotOJCoHa. Report on the Cost of Prescription Drugs in Ireland 2015. 2015.
3. Brick A GP, Nolan A. Ireland: Pharmaceutical Prices, Prescribing Practices and Usage of Generics in a Comparative Context. Dublin: ESRI, 2013.
4. Layte R. (ed) BM, Bennett K, Brick A, Morgenroth E., Normand C, O'Reilly J, Thomas S, Tilson L, Wiley M, Wren M.-A. Demographic Change on the Demand for and Delivery of Health Care in Ireland. Dublin: ESRI, 2009.
5. Programme MM. Welcome to the Medicines Management Programme 2016. Available from: http://www.hse.ie/eng/about/Who/clinical/natclinprog/medicinemanagementprogramme/yourmedicines/.
6. Programme MM. Evaluation Reports 2017. Available from: http://www.hse.ie/eng/about/Who/clinical/natclinprog/medicinemanagementprogramme/yourmedicines/Evaluation_Reports/.
7. von Elm E, Altman DG, Egger M, Pocock SJ, Gotzsche PC, Vandenbroucke JP, et al. The Strengthening the Reporting of Observational Studies in Epidemiology (STROBE) statement: guidelines for reporting observational studies. Journal of clinical epidemiology. 2008;61(4):344-9.
8. Wagner AK, Soumerai SB, Zhang F, Ross-Degnan D. Segmented regression analysis of interrupted time series studies in medication use research. Journal of clinical pharmacy and therapeutics. 2002;27(4):299-309.
9. Education UIfDRa. How can I run a piecewise regression in Stata? 2017. Available from: https://stats.idre.ucla.edu/stata/faq/how-can-i-run-a-piecewise-regression-in-stata/.
10. StataCorp. Stata Statistical Software. Texas: StataCorp; 2015.
11. Davis D, O'Brien MA, Freemantle N, Wolf FM, Mazmanian P, Taylor-Vaisey A. Impact of formal continuing medical education: do conferences, workshops, rounds, and other traditional continuing education activities change physician behavior or health care outcomes? Jama. 1999;282(9):867-74.
12. Gill PS, Makela M, Vermeulen KM, Freemantle N, Ryan G, Bond C, et al. Changing doctor prescribing behaviour. Pharmacy world & science : PWS. 1999;21(4):158-67.
13. Brufsky JW, Ross-Degnan D, Calabrese D, Gao X, Soumerai SB. Shifting physician prescribing to a preferred histamine-2-receptor antagonist. Effects of a multifactorial intervention in a mixed-model health maintenance organization. Medical care. 1998;36(3):321-32.
14. Godman B, Wettermark B, van Woerkom M, Fraeyman J, Alvarez-Madrazo S, Berg C, et al. Multiple policies to enhance prescribing efficiency for established medicines in Europe with a particular focus on demand-side measures: findings and future implications. Frontiers in pharmacology. 2014;5:106.
15. Soumerai SB, Avorn J. Principles of educational outreach ('academic detailing') to improve clinical decision making. Jama. 1990;263(4):549-56.
16. Kuperman GJ, Bobb A, Payne TH, Avery AJ, Gandhi TK, Burns G, et al. Medication-related clinical decision support in computerized provider order entry systems: a review. Journal of the American Medical Informatics Association : JAMIA. 2007;14(1):29-40.
17. Australian Government DoH. Available from: https://www.healthdirect.gov.au/statins.
18. Persaud N, Ahmad H. Canadian list of essential medications: Potential and uncertainties. Canadian family physician Medecin de famille canadien. 2017;63(4):266-8.

For peer review only - http://bmjopen.bmj.com/site/about/guidelines.xhtml

19. Wettermark B, Pehrsson A, Jinnerot D, Bergman U. Drug utilisation 90% profiles--a useful tool for quality assessment of prescribing in primary health care in Stockholm. Pharmacoepidemiology and drug safety. 2003;12(6):499-510.
20. Eriksen J, Gustafsson LL, Ateva K, Bastholm-Rahmner P, Ovesjo ML, Jirlow M, et al. High adherence to the 'Wise List' treatment recommendations in Stockholm: a 15-year retrospective review of a multifaceted approach promoting rational use of medicines. BMJ open. 2017;7(4):e014345.
21. Gustafsson LL, Wettermark B, Godman B, Andersen-Karlsson E, Bergman U, Hasselstrom J, et al. The 'wise list'- a comprehensive concept to select, communicate and achieve adherence to recommendations of essential drugs in ambulatory care in Stockholm. Basic & clinical pharmacology & toxicology. 2011;108(4):224-33.
22. Rector TS, Finch MD, Danzon PM, Pauly MV, Manda BS. Effect of tiered prescription copayments on the use of preferred brand medications. Medical care. 2003;41(3):398-406.
23. Sinnott SJ, Normand C, Byrne S, Woods N, Whelton H. Copayments for prescription medicines on a public health insurance scheme in Ireland. Pharmacoepidemiology and drug safety. 2016;25(6):695-704.
24. Huskamp HA, Deverka PA, Epstein AM, Epstein RS, McGuigan KA, Frank RG. The effect of incentive-based formularies on prescription-drug utilization and spending. The New England journal of medicine. 2003;349(23):2224-32.
25. Morgan SG, Leopold C, Wagner AK. Drivers of expenditure on primary care prescription drugs in 10 high-income countries with universal health coverage. CMAJ : Canadian Medical Association journal = journal de l'Association medicale canadienne. 2017;189(23):E794-E9.
26. Walley T, Murphy M, Codd M, Johnston Z, Quirke T. Effects of a monetary incentive on primary care prescribing in Ireland: changes in prescribing patterns in one health board 1990-1995. Pharmacoepidemiology and drug safety. 2000;9(7):591-8.
27. Flodgren G, Eccles MP, Shepperd S, Scott A, Parmelli E, Beyer FR. An overview of reviews evaluating the effectiveness of financial incentives in changing healthcare professional behaviours and patient outcomes. The Cochrane database of systematic reviews. 2011(7):CD009255.
28. Roland M. Incentives must be closely aligned to professional values. Bmj. 2012;345:e5982.
29. Majumdar SR, Soumerai SB. Why most interventions to improve physician prescribing do not seem to work. CMAJ : Canadian Medical Association journal = journal de l'Association medicale canadienne. 2003;169(1):30-1.
30. Persaud N, Lee T, Ahmad H, Li W, Taglione MS, Rajakulasingam Y, et al. Protocol for a randomised controlled trial evaluating the effects of providing essential medicines at no charge: the Carefully seLected and Easily Accessible at No Charge Medicines (CLEAN Meds) trial. BMJ open. 2017;7(5):e015686.

For peer review only - http://bmjopen.bmj.com/site/about/guidelines.xhtml

Table 1:Summary of impact of Preferred Drugs Initiative (2011-2016)

Preferred drug class	PPI	Statin	ACE	ARB	SNRI	SSRI	Urology	Total
Total no. items	18,939,282	19,944,634	8,837,006	5,171,204	3,345,307	8,348,567	2,239,263	336,535,263
% of all drugs	5.63%	5.93%	2.63%	1.54%	0.99%	2.48%	0.67%	19.86%
**Preferred drug**	**Lansoprazole**	**Simvastatin**	**Ramipril**	**Candesartan**	**Venlafaxine**	**Citalopram**	**ER Tolterodine**	
**Total no. single-agent items**	4,571,751	1,313,389	4,719,996	557,622	1,155,600	1,650,520	577,540	
**% within class**	24.14%	6.59%	53.41%	10.78%	70.99%	19.77%	25.79%	
**Rank within class pre-PDS**	2/5	4/5	1/10	5/8	1/2	2/6	1/9	
**Rank within class post-PDS**	2/5	4/5	1/10	5/8	1/2	3/6	3/9	
**Absolute change in proportion of preferred drug items: first 3 months post-PDS v previous 3 months**	↑ +0.98% (p<0.001)	↑ +0.30% (p<0.001)	↑ 0.53% (p<0.001)	↓ -0.03% (p=0.76)	↑ 0.30% (p=0.08)	↓ -0.09% (p=0.37)	↓ -0.98% (p<0.001)	

PPI- Proton Pump Inhibitor; ACE- Angiotensin Receptor Antagonist; ARB- Angiotensin Receptor Blocker; SSRI- Selective Serotonin Receptor Antagonists; SNRI- Serotonin and Noradrenaline Reuptake Inhibitors; ER: Extended Release; PDS: Preferred Drug Scheme

For peer review only - http://bmjopen.bmj.com/site/about/guidelines.xhtml

Figure 1: Distribution of preferred drug items by therapeutic drug class

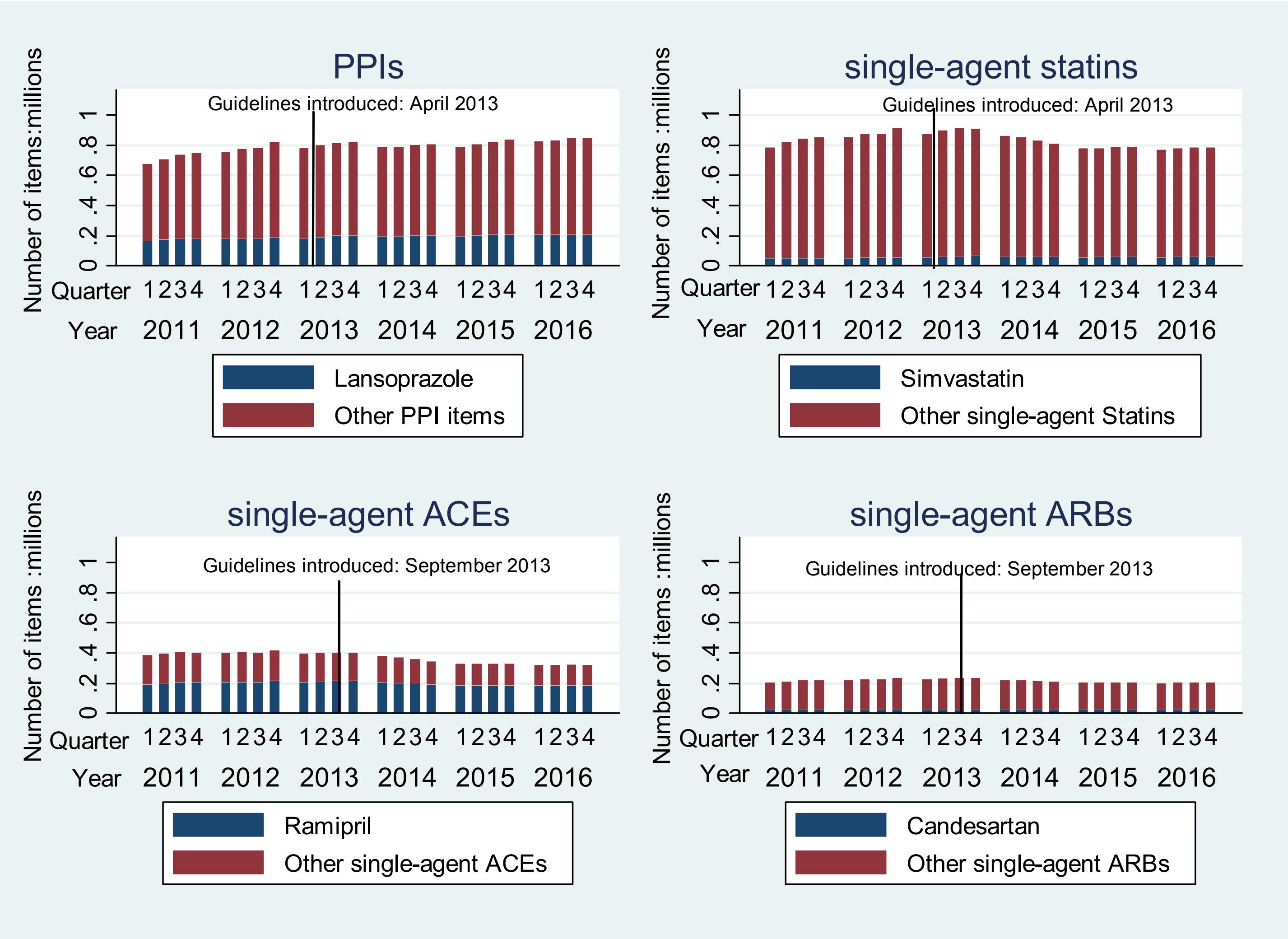

For peer review only - http://bmjopen.bmj.com/site/about/guidelines.xhtml

Figure 1 (cont): Distribution of preferred drug items by therapeutic drug class

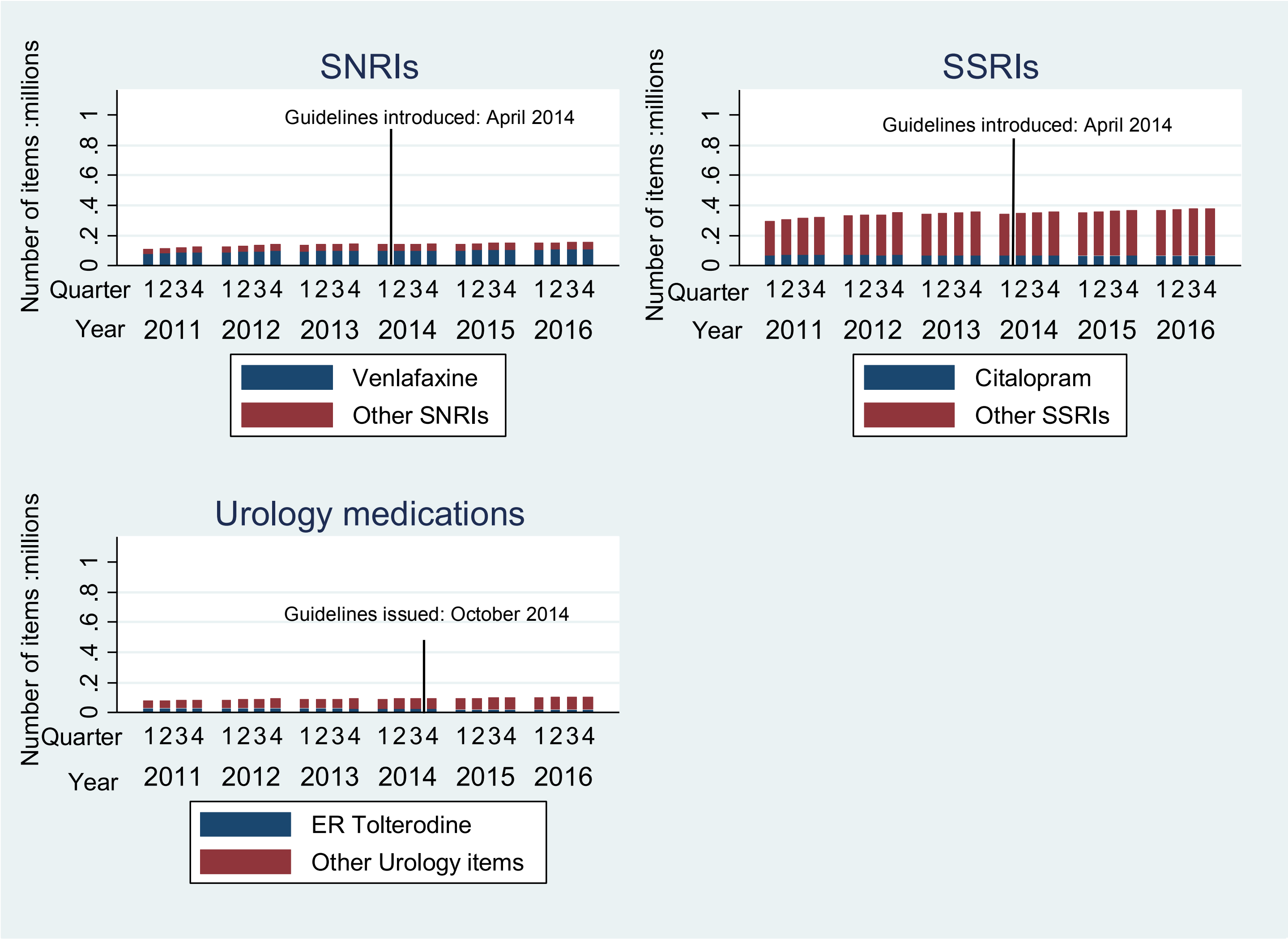

For peer review only - http://bmjopen.bmj.com/site/about/guidelines.xhtml

Table 2: Segmented regression analysis in relation to guideline publication and cost savings

Medicine group	Preferred drug	Guidelines introduced	Odds of preferred drug Jan-Mar 2011 (SE), (95% CI)	OR: change in odds of preferred drug per quarter pre-guidelines (SE), (95%CI), p-value	OR: change in odds of preferred drug in quarter immediately following guidelines (SE), (95%CI), p-value	OR: change in odds of preferred drug per quarter post guidelines (SE) (95%CI), p-value	Estimated savings between issuing of guidelines and Dec 2016 (€)
**PPIs**	**Lansoprazole**	April 2013	0.32 (0.001), (0.32, 0.32)	0.99 (0.001), (0.98,0.99), p<0.001	1.12 (0.003), (1.11,1.12), p<0.001	1.00 (0.001), (1.00,1.00), p<0.001	807,990
**Statins**	**Simvastatin**	April 2013	0.06 (0.001) (0.06, 0.06)	1.00 (0.001), (1.00,1.00), p=0.006	1.12 (0.004), (1.11,1.13), p<0.001	1.01 (0.001), (1.01,1.01), p<0.001	563,610
**ACEs**	**Ramipril**	Sept 2013	0.97 (0.002), (0.96,0.97)	1.02 (0.001), (1.01,1.02), p <0.001	1.01 (0.003), (1.00,1.01), p=0.008	1.02 (0.001), (1.02,1.02), p <0.001	5,059
**ARBs**	**Candesartan**	Sept 2013	0.14 (0.001), (0.13,0.14)	0.98 (0.001), (0.98,0.99), p<0.001	1.04 (0.006), (1.02,1.05), p<0.001	1.00 (0.001), (1.00,1.00), p =0.65	66,134
**SNRIs**	**Venlafaxine**	April 2014	2.75 (0.01), (2.73,2.77)	0.98 (0.001), (0.98,0.98), p<0.001	1.08 (0.01), (1.07,1.09), p<0.001	1.01 (0.001), (1.00,1.01), p<0.001	1,376,617
**SSRIs**	**Citalopram**	April 2014	0.31 (0.001), (0.31,0.31)	0.98 (0.001), (0.98,0.98), p <0.001	1.03 (0.004), (1.02,1.04), p<0.001	0.98 (0.001), (0.98,0.98), p <0.001	152,326
**Urology**	**ER Tolterodine**	October 2014	0.60 (0.002), (0.60,0.60)	0.95 (0.001), (0.95,0.96), p <0.001	0.95 (0.01), (0.93,0.96), p <0.001	0.96 (0.001), (0.96,0.96), p <0.001	-64,835
**Total**							**2,907,081**

OR: Odds ratio; CI: Confidence Interval; SE: Standard Error; ER: Extended Release

For peer review only - http://bmjopen.bmj.com/site/about/guidelines.xhtml

Fig 2 Estimated proportion of preferred drugs by drug class: segmented regression models

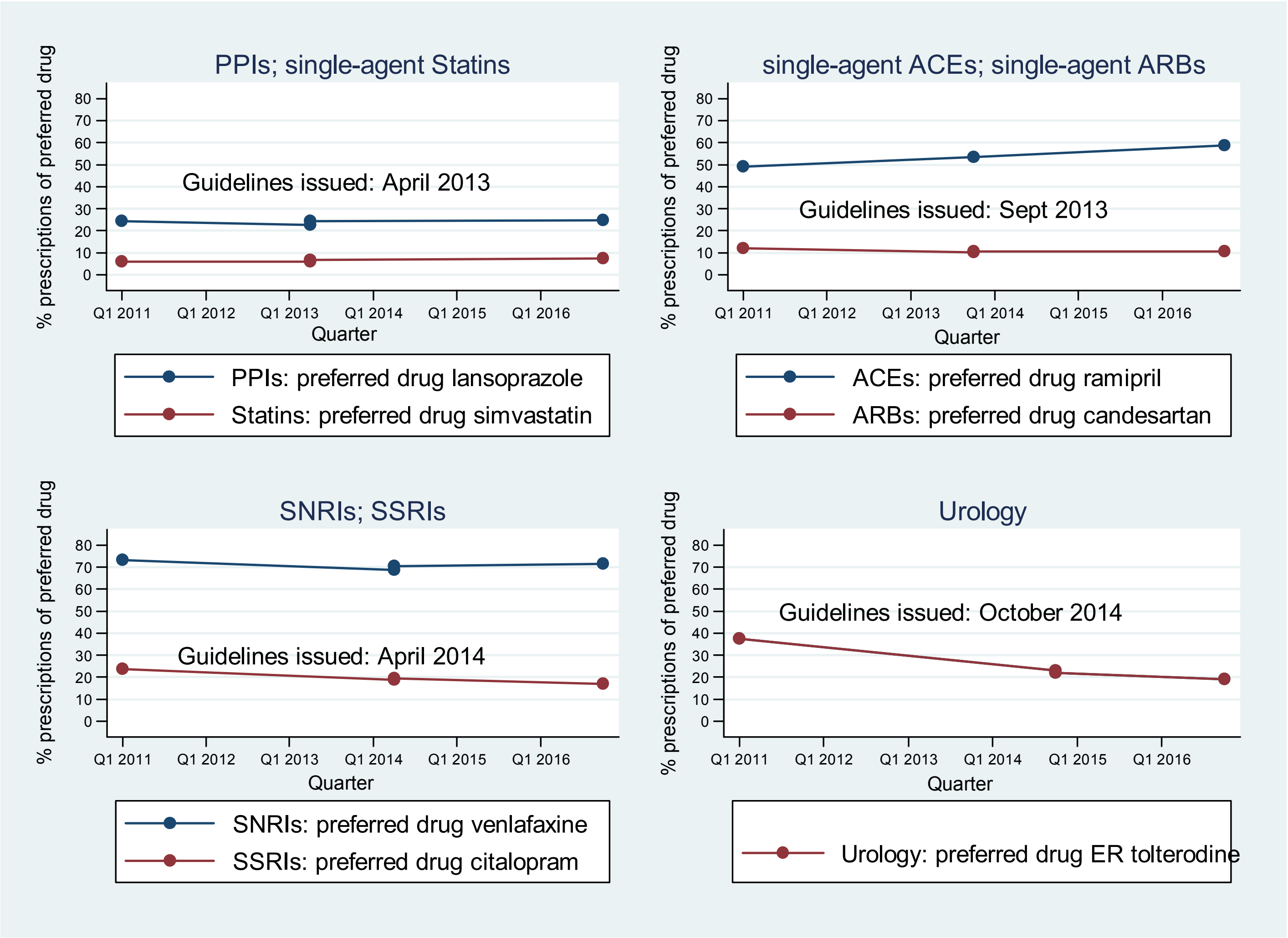

Appendix

Table A1: Breakdown of PCRS reimbursed items: 2011-2016

Year	No. items issued	No. single-agent items issued across 7 drug classes*	% of items attributed to 7 drug classes*	No. items issued for preferred drugs	% preferred drug items within preferred drug classes	% preferred drug items across all prescriptions
**2011**	54,324,492	10,630,476	19.57%	2,520,986	23.71%	4.64%
**2012**	57,984,934	11,380,582	19.63%	2,641,897	23.21%	4.56%
**2013**	58,455,927	11,640,615	19.91%	2,708,855	23.27%	4.63%
**2014**	55,978,157	11,181,081	19.97%	2,655,422	23.75%	4.74%
**2015**	54,573,162	10,925,162	20.02%	2,610,926	23.90%	4.78%
**2016**	55,218,591	11,067,347	20.04%	2,627,631	23.74%	4.76%
**Total**	336,553,263	66,825,263	19.86%	15,765,717	23.59%	4.68%

***PPIs, Statins, ACEs, ARBs, SNRIs, SSRIs, Urology**

Table A2 Prevalence of PCRS reimbursed items by therapeutic drug class (single agent drugs)

Year	PPIs	Statins	ACEs	ARBs	SNRIs	SSRIs	Urology	Other	Total
**2011**	2,860,986 (5.27%)	3,286,352 (6.05%)	1,586,992 (2.92%)	849,807 (1.56%)	470,234 (0.87%)	1,247,643 (2.30%)	328,462 (0.60%)	43,694,016 (80.43%)	54,324,492 (100%)
**2012**	3,114,214 (5.37%)	3,501,257 (6.04%)	1,616,612 (2.79%)	899,594 (1.55%)	537,800 (0.93%)	1,355,921 (2.34%)	355,184 (0.61%)	46,604,352 (80.37%)	57,984,934 (100%)
**2013**	3,203,104 (5.48%)	3,582,112 (6.13%)	1,595,582 (2.73%)	920,851 (1.58%)	566,951 (0.97%)	1,404,466 (2.40%)	367,549 (0.63%)	46,815,312 (80.09%)	58,455,927 (100%)
**2014**	3,180,702 (5.68%)	3,339,227 (5.97%)	1,449,173 (2.59%)	867,567 (1.55%)	567,859 (1.01%)	1,399,724 (2.50%)	376,829 (0.67%)	44,797,076 (80.03%)	55,978,157 (100%)
**2015**	3,241,661 (5.94%)	3,129,117 (5.73%)	1,312,155 (2.40%)	816,250 (1.50%)	588,689 (1.08%)	1,441,270 (2.64%)	396,020 (0.73%)	43,648,000 (79.98%)	54,573,162 (100%)
**2016**	3,338,615 (6.05%)	3,106,569 (5.63%)	1,276,492 (2.31%)	817,135 (1.48%)	613,774 (1.11%)	1,499,543 (2.72%)	415,219 (0.75%)	44,151,244 (79.96%)	55,218,591 (100%)
**Total**	18,939,282 (5.63%)	19,944,634 (5.93%)	8,837,006 (2.63%)	5,171,204 (1.54%)	3,345,307 (0.99%)	8,348,567 (2.48%)	2,239,263 (0.67%)	269,710,000 (80.14%)	336,535,263 (100%)

For peer review only

Fig A1: Breakdown of PCRS reimbursed items by Preferred Drug status: 2011-2016

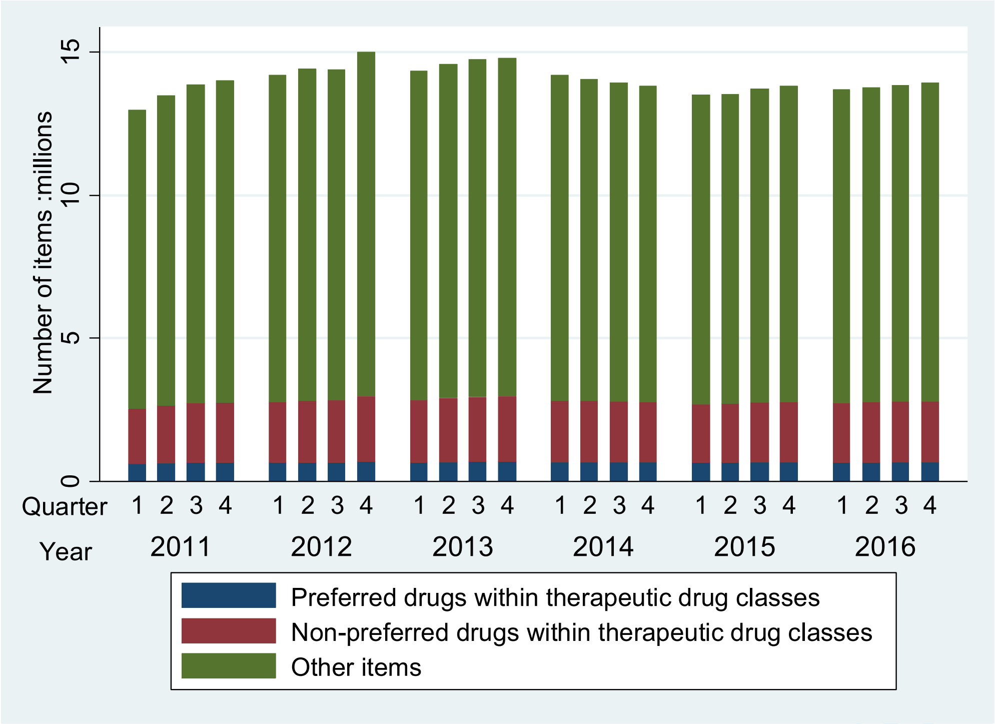

Fig A2: Observed proportion of preferred drugs by drug class

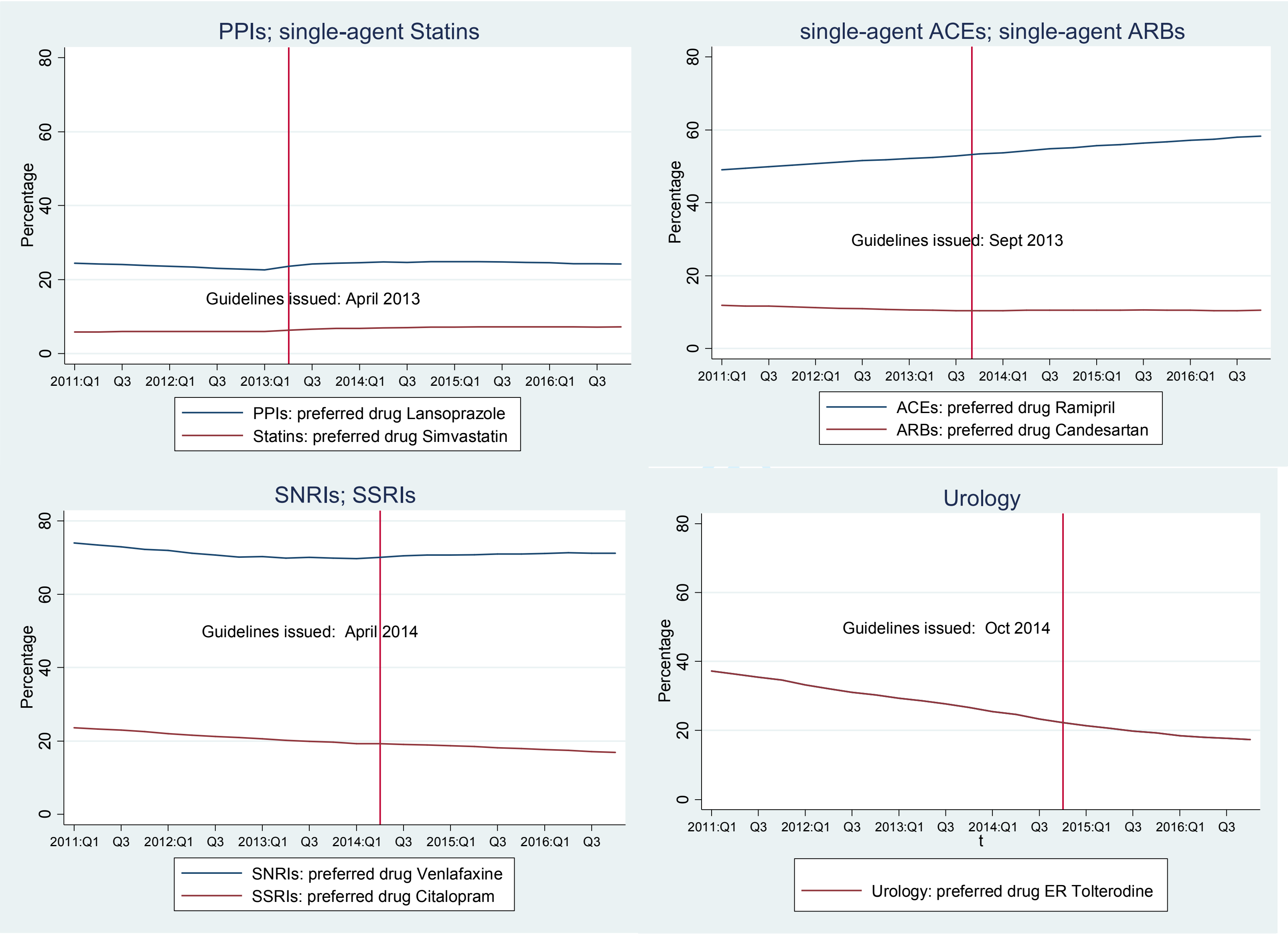

Table A3: Sensitivity analyses: alternative definition of calendar quarters for ACEs/ARBs

	**Calendar quarters: Jan-Mar, Apr-Jun, Jul-Sep, Oct-Dec (24 calendar quarters: Jan 11-Dec 16)**	**Calendar quarters: Mar-May, Jun-Aug, Sep-Nov, Dec-Feb (23 calendar quarters: Mar 11-Nov 16)**
**Ramipril**		
Odds of preferred drug at start of study period (SE), (95% CI)	0.97 (0.02), (0.96,0.97)	0.98 (0.02), (0.97,0.98)
OR: change in odds of preferred drug per quarter pre- guidelines (SE), (95%CI), p-value	1.02 (0.001), (1.01,1.02), p<0.001	1.01 (0.001), (1.01,1.02), P<0.001
OR: change in odds of preferred drug in quarter immediately following guidelines (SE), (95%CI), p-value	1.01 (0.003), (1.00,1.01), p=0.008	1.01 (0.003), (1.01,1.02), P<0.001
OR: change in odds of preferred drug per quarter post guidelines (SE) (95%CI), p-value	1.02 (0.001) (1.02,1.02), p<0.001	1.02 (0.001) (1.01,1.02), p<0.001
**Candesartan**		
Odds of preferred drug at start of study period (SE), (95% CI)	0.14 (0.001), (0.13,0.14)	0.14 (0.001). (0.13,0.13)
OR: change in odds of preferred drug per quarter pre- guidelines (SE), (95%CI), p-value	0.98 (0.001), (0.98,0.99), p<0.001	0.98 (0.001), (0.98,0.99), p<0.001
OR: change in odds of preferred drug in quarter immediately following guidelines (SE), (95%CI), p-value	1.04 (0.006), (1.02,1.05), p<0.001	1.03 (0.006), (1.02,1.05), p<0.001
OR: change in odds of preferred drug per quarter post guidelines (SE) (95%CI), p-value	1.00 (0.001), (1.00,1.00), p=0.65	1.00 (0.001), (1.00,1.00), p=0.54

For peer review only

Table A4: Sensitivity analyses for segmented regression models

	Calendar quarters retained for analysis (Study Period)			
	**All available data: 9 quarters before guidelines, 15 quarters after guidelines, (Jan 11- Dec 16)**	**9 quarters before guidelines, 13 quarters after guidelines (Jan 11-Jun 16)**	**9 quarters before guidelines, 11 quarters after guidelines (Jan 11-Dec 15)**	**9 quarters before guidelines, 9 quarters after guidelines (Jan 11-Jun 16)**
**Lansoprazole**				
Odds of preferred drug at start of study period (SE), (95% CI)	0.32 (0.001), (0.32,0.32)	0.32 (0.001), (0.32,0.32)	0.32 (0.001), (0.32,0.32)	0.32 (0.001), (0.32,0.32)
OR: change in odds of preferred drug per quarter pre- guidelines (SE), (95%CI), p-value	0.99 (0.001), (0.98,0.99), p <0.001	0.99 (0.001), (0.99,0.99), p <0.001,	0.99 (0.001), (0.99,0.99), p <0.001,	0.99 (0.001), (0.99,0.99), p <0.001,
OR: change in odds of preferred drug in quarter immediately following guidelines (SE), (95%CI), p-value	1.12 (0.003), (1.11,1.12), p<0.001	1.11 (0.003), (1.10,1.11), p<0.001	1.10 (0.003), (1.10,1.11), p<0.001	1.09 (0.003), (1.09,1.10), p<0.001
OR: change in odds of preferred drug per quarter post guidelines (SE) (95%CI), p-value	1.00 (0.001), (1.00,1.00), p<0.001	1.00 (0.001), (1.00,1.00), p<0.001	1.00 (0.001), (1.00,1.00), p<0.001	1.01 (0.001), (1.01,1.01), p<0.001
**Simvastatin**				
Odds of preferred drug at start of study period (SE), (95% CI)	0.06 (0.001), (0.06,0.06)	0.06 (0.001), (0.06,0.06)	0.06 (0.001), (0.06,0.06)	0.06 (0.001), (0.06,0.06)
OR: change in odds of preferred drug per quarter pre- guidelines (SE), (95%CI), p-value	1.00 (0.001), (1.00,1.00), p=0.006	1.00 (0.001), (1.00,1.00), p=0.006	1.00 (0.001), (1.00,1.00), p=0.006	1.00 (0.001), (1.00,1.00), p=0.006
OR: change in odds of preferred drug in quarter immediately following guidelines (SE), (95%CI), p-value	1.12 (0.004), (1.11,1.13), p<0.001	1.11 (0.004), (1.10,1.12), p<0.001	1.10 (0.004), (1.09,1.11), p<0.001	1.09 (0.005), (1.08,1.10), p<0.001
OR: change in odds of preferred drug per quarter post guidelines (SE) (95%CI), p-value	1.01 (0.001), (1.01,1.01), p<0.001	1.01 (0.001), (1.01,1.01), p<0.001	1.01 (0.001), (1.01,1.01), p<0.001	1.02 (0.001), (1.02,1.02), p<0.001

For peer review only

Table A4 (cont): sensitivity analyses for segmented regression models

	Calendar quarters retained for analysis (Study Period)					
	**All available data: 11 quarters before guidelines, 13 quarters after guidelines (Jan 11-Dec 16)**	**11 quarters before guidelines, 11 quarters after guidelines (Jan 11-Jun 16)**	**11 quarters before guidelines, 9 quarters after guidelines (Jan 11-Dec 15)**	**9 quarters before guidelines, 13 quarters after guidelines (Jul 11-Dec 16)**	**9 quarters before guidelines, 11 quarters after guidelines (Jul 11-Jun 16)**	**9 quarters before guidelines, 9 quarters after guidelines (Jul 11-Jan 15)**
**Ramipril**						
Odds of preferred drug at start of study period (SE), (95% CI)	0.97 (0.02), (0.96,0.97)	0.97 (0.02), (0.96,0.97)	0.97 (0.02), (0.96,0.97)	1.00 (0.02), (0.99,1.00)	1.00 (0.02), (0.99,1.00)	1.00 (0.02), (1.00,1.00)
OR: change in odds of preferred drug per quarter pre- guidelines (SE), (95%CI), p-value	1.02 (0.001), (1.01,1.02), p<0.001	1.02 (0.001), (1.01,1.02), p<0.001	1.02 (0.001), (1.01,1.02), p<0.001	1.02 (0.001), (1.01,1.02), p<0.001	1.01 (0.001), (1.01,1.02), p<0.001	1.01 (0.001), (1.01,1.02), p<0.001
OR: change in odds of preferred drug in quarter immediately following guidelines (SE), (95%CI), p-value	1.01 (0.003), (1.00,1.01), p=0.008	1.01 (0.003), (1.00,1.01), p=0.01	1.01 (0.003), (1.00,1.01), p=0.04	1.01 (0.003), (1.00,1.01), p=0.002	1.01 (0.003), (1.00,1.01), p=0.003	1.01 (0.003), (1.00,1.01), p=0.008
OR: change in odds of preferred drug per quarter post guidelines (SE) (95%CI), p-value	1.02 (0.001) (1.02,1.02), p<0.001	1.02 (0.001) (1.02,1.02), p<0.001	1.02 (0.001) (1.02,1.02), p<0.001	1.02 (0.001) (1.02,1.02), p<0.001	1.02 (0.001) (1.02,1.02), p<0.001	1.02 (0.001) (1.02,1.02), p<0.001
**Candesartan**						
Odds of preferred drug at start of study period (SE), (95% CI)	0.14 (0.001), (0.13,0.14)	0.14 (0.001), (0.13,0.14)	0.14 (0.001), (0.13,0.14)	0.13 (0.001), (0.13,0.13)	0.13 (0.001), (0.13,0.14)	0.13 (0.001), (0.13,0.14)
OR: change in odds of preferred drug per quarter pre- guidelines (SE), (95%CI), p-value	0.98 (0.001), (0.98,0.99), p<0.001	0.98 (0.001), (0.98,0.99), p<0.001	0.98 (0.001), (0.98,0.99), p<0.001	0.98 (0.001), (0.98,0.99), p<0.001	0.98 (0.001), (0.98,0.99), p<0.001	0.98 (0.001), (0.98,0.99), p<0.001
OR: change in odds of preferred drug in quarter immediately following guidelines (SE), (95%CI), p-value	1.04 (0.006), (1.02,1.05), p<0.001	1.04 (0.006), (1.02,1.05), p<0.001	1.04 (0.006), (1.02,1.05), p<0.001	1.03 (0.006), (1.02,1.05), p<0.001	1.03 (0.007), (1.02,1.05), p<0.001	1.03 (0.006), (1.02,1.04), p<0.001
OR: change in odds of preferred drug per quarter post guidelines (SE) (95%CI), p-value	1.00 (0.001), (1.00,1.00), p=0.65	1.00 (0.001), (1.00,1.00), p=0.98	1.00 (0.001), (1.00,1.00), p=0.28	1.00 (0.001), (1.00,1.00), p=0.65	1.00 (0.001), (1.00,1.00), p=0.95	1.00 (0.001), (1.00,1.00), p=0.28

Table A4 (cont): sensitivity analyses for segmented regression models

	Calendar quarters retained for analysis (Study Period)					
	**All available data: 13 quarters before guidelines, 11 quarters after guidelines (Jan 11-Dec 16)**	**13 quarters before guidelines, 9 quarters after guidelines (Jan 11-Jun 16)**	**11 quarters before guidelines, 11 quarters after guidelines (Jul 11-Dec 16)**	**11 quarters before guidelines, 9 quarters after guidelines (Jul 11-Jun 16)**	**9 quarters before guidelines, 11 quarters after guidelines (Jan 12-Dec 16)**	**9 quarters before guidelines, 9 quarters after guidelines (Jan 12-Jun 16)**
**Venlafaxine**						
Odds of preferred drug at start of study period (SE), (95% CI)	2.75 (0.01), (2.73,2.77)	2.75 (0.01), (2.73,2.77)	2.62 (0.01), (2.59,2.63)	2.61 (0.01), (2.59,2.63)	2.49 (0.01), (2.47,2.51)	2.49 (0.01), (2.47,2.51)
OR: change in odds of preferred drug per quarter pre- guidelines (SE), (95%CI), p-value	0.98 (0.001), (0.98,0.98), p<0.001	0.98 (0.001), (0.98,0.98), p<0.001	0.99 (0.001), (0.98,0.99), p<0.001	0.99 (0.001), (0.98,0.99), p<0.001	0.99 (0.001), (0.99,0.99), p<0.001	0.99 (0.001), (0.99,0.99), p<0.001
OR: change in odds of preferred drug in quarter immediately following guidelines (SE), (95%CI), p-value	1.08 (0.01), (1.07,1.09), p<0.001	1.08 (0.01), (1.07,1.09), p<0.001	1.07 (0.01), (1.06,1.08), p<0.001	1.07 (0.01), (1.06,1.08), p<0.001	1.06 (0.01), (1.04,1.07), p<0.001	1.06 (0.01), (1.04,1.06), p<0.001
OR: change in odds of preferred drug per quarter post guidelines (SE) (95%CI), p-value	1.01 (0.001), (1.00,1.01), p<0.001	1.01 (0.001), (1.01,1.01), p<0.001	1.01 (0.001), (1.00,1.01), p<0.001	1.01 (0.001), (1.01,1.01), p<0.001	1.01 (0.001), (1.00,1.01), p<0.001	1.01 (0.001), (1.01,1.01), p<0.001
**Citalopram**						
Odds of preferred drug at start of study period (SE), (95% CI)	0.31 (0.001), (0.31,0.31)	0.31 (0.001), (0.31,0.31)	0.30 (0.001), (0.29,0.30)	0.30 (0.001), (0.29,0.30)	0.28 (0.001), (0.28,0.28)	0.28 (0.001), (0.28,0.28)
OR: change in odds of preferred drug per quarter pre- guidelines (SE), (95%CI), p-value	0.98 (0.001), (0.98,0.98), p<0.001	0.98 (0.001), (0.98,0.98), p<0.001	0.98 (0.001), (0.98,0.98), p<0.001	0.98 (0.001), (0.98,0.98), p<0.001	0.98 (0.001), (0.98,0.98), p<0.001	0.98 (0.001), (0.98,0.98), p<0.001
OR: change in odds of preferred drug in quarter immediately following guidelines (SE), (95%CI), p-value	1.03 (0.004), (1.02,1.04), p<0.001	1.03 (0.004), (1.02,1.04), p<0.001	1.03 (0.004), (1.02,1.04), p<0.001	1.03 (0.004), (1.02,1.04), p<0.001	1.03 (0.004), (1.02,1.03), p<0.001	1.02 (0.004), (1.01,1.03), p<0.001
OR: change in odds of preferred drug per quarter post guidelines (SE) (95%CI), p-value	0.98 (0.001), (0.98,0.98), p<0.001	0.98 (0.001), (0.98,0.99), p<0.001	0.98 (0.001), (0.98,0.99), p<0.001	0.98 (0.001), (0.98,0.99), p<0.001	0.98 (0.001), (0.98,0.98), p<0.001	0.98 (0.001), (0.98,0.99), p<0.001

Table A4 (cont): sensitivity analyses for segmented regression models

	Calendar quarters retained for analysis (Study Period)			
	**All available data: 15 quarters before guidelines, 9 quarters after guidelines (Jan 11-Dec 16)**	**13 quarters before guidelines, 9 quarters after guidelines (Jul 11-Dec 16)**	**11 quarters before guidelines, 9 quarters after guidelines (Jan 12-Dec 16)**	**9 quarters before guidelines, 9 quarters after guidelines (Jul 12-Dec 16)**
**ER Tolterodine**				
Odds of preferred drug at start of study period (SE), (95% CI)	0.60 (0.002), (0.60,0.60)	0.55 (0.002), (0.55,0.55)	0.52 (0.002), (0.51,0.52)	0.46 (0.002), (0.45,0.46)
OR: change in odds of preferred drug per quarter pre- guidelines (SE), (95%CI), p-value	0.95 (0.001), (0.95,0.96), p<0.001	0.95 (0.001), (0.95,0.95), p<0.001	0.96 (0.001), (0.95,0.96), p<0.001	0.95 (0.001), (0.95,0.95), p<0.001
OR: change in odds of preferred drug in quarter immediately following guidelines (SE), (95%CI), p-value	0.95 (0.01), (0.93,0.96), p<0.001	0.95 (0.01), (0.94,0.96), p<0.001	0.94 (0.01), (0.93,0.95), p<0.001	0.95 (0.01), (0.94,0.97), p<0.001
OR: change in odds of preferred drug per quarter post guidelines (SE) (95%CI), p-value	0.96 (0.001), (0.96,0.96), p<0.001	0.96 (0.001), (0.96,0.96), p<0.001	0.96 (0.001), (0.96,0.96), p<0.001	0.96 (0.001), (0.96,0.96), p<0.001

For peer review only

STROBE 2007 (v4) checklist of items to be included in reports of observational studies in epidemiology*
Checklist for cohort, case-control, and cross-sectional studies (combined)

Section/Topic	Item #	Recommendation	Reported on page #
**Title and abstract**	1	(*a*) Indicate the study's design with a commonly used term in the title or the abstract	5
		(*b*) Provide in the abstract an informative and balanced summary of what was done and what was found	2
**Introduction**			
Background/rationale	2	Explain the scientific background and rationale for the investigation being reported	4
Objectives	3	State specific objectives, including any pre-specified hypotheses	4
**Methods**			
Study design	4	Present key elements of study design early in the paper	5
Setting	5	Describe the setting, locations, and relevant dates, including periods of recruitment, exposure, follow-up, and data collection	5
Participants	6	(*a*) *Cohort study*—Give the eligibility criteria, and the sources and methods of selection of participants. Describe methods of follow-up<br>*Case-control study*—Give the eligibility criteria, and the sources and methods of case ascertainment and control selection. Give the rationale for the choice of cases and controls<br>*Cross-sectional study*—Give the eligibility criteria, and the sources and methods of selection of participants	5
		(*b*) *Cohort study*—For matched studies, give matching criteria and number of exposed and unexposed<br>*Case-control study*—For matched studies, give matching criteria and the number of controls per case	
Variables	7	Clearly define all outcomes, exposures, predictors, potential confounders, and effect modifiers. Give diagnostic criteria, if applicable	5
Data sources/ measurement	8*	For each variable of interest, give sources of data and details of methods of assessment (measurement). Describe comparability of assessment methods if there is more than one group	*5*
Bias	9	Describe any efforts to address potential sources of bias	10-all available data used
Study size	10	Explain how the study size was arrived at	5-all available data used
Quantitative variables	11	Explain how quantitative variables were handled in the analyses. If applicable, describe which groupings were chosen and why	5-groupings as per medicine group
Statistical methods	12	(*a*) Describe all statistical methods, including those used to control for confounding	5/6
		(*b*) Describe any methods used to examine subgroups and interactions	n/a

For peer review only

		(*c*) Explain how missing data were addressed	n/a
		(*d*) *Cohort study*—If applicable, explain how loss to follow-up was addressed<br>*Case-control study*—If applicable, explain how matching of cases and controls was addressed<br>*Cross-sectional study*—If applicable, describe analytical methods taking account of sampling strategy	n/a
		(*e*) Describe any sensitivity analyses	6, Appendix
**Results**			
Participants	13*	(a) Report numbers of individuals at each stage of study—eg numbers potentially eligible, examined for eligibility, confirmed eligible, included in the study, completing follow-up, and analysed	7
		(b) Give reasons for non-participation at each stage	n/a
		(c) Consider use of a flow diagram	N/A
Descriptive data	14*	(a) Give characteristics of study participants (eg demographic, clinical, social) and information on exposures and potential confounders	N/A
		(b) Indicate number of participants with missing data for each variable of interest	N/A
		(c) *Cohort study*—Summarise follow-up time (eg, average and total amount)	All data 2011-2016 used
Outcome data	15*	*Cohort study*—Report numbers of outcome events or summary measures over time	N/a
		Case-control study—Report numbers in each exposure category, or summary measures of exposure	N/A
		Cross-sectional study—Report numbers of outcome events or summary measures	7, Table 1
Main results	16	(*a*) Give unadjusted estimates and, if applicable, confounder-adjusted estimates and their precision (eg, 95% confidence interval). Make clear which confounders were adjusted for and why they were included	7,8
		(*b*) Report category boundaries when continuous variables were categorized	n/a
		(*c*) If relevant, consider translating estimates of relative risk into absolute risk for a meaningful time period	8
Other analyses	17	Report other analyses done—eg analyses of subgroups and interactions, and sensitivity analyses	8, Appendix
**Discussion**			
Key results	18	Summarise key results with reference to study objectives	9
Limitations	19	Discuss limitations of the study, taking into account sources of potential bias or imprecision. Discuss both direction and magnitude of any potential bias	10
Interpretation	20	Give a cautious overall interpretation of results considering objectives, limitations, multiplicity of analyses, results from similar studies, and other relevant evidence	9/10
Generalisability	21	Discuss the generalisability (external validity) of the study results	9/10
**Other information**			
Funding	22	Give the source of funding and the role of the funders for the present study and, if applicable, for the original study on which the present article is based	12

For peer review only

*Give information separately for cases and controls in case-control studies and, if applicable, for exposed and unexposed groups in cohort and cross-sectional studies.
Note: An Explanation and Elaboration article discusses each checklist item and gives methodological background and published examples of transparent reporting. The STROBE checklist is best used in conjunction with this article (freely available on the Web sites of PLoS Medicine at http://www.plosmedicine.org/, Annals of Internal Medicine at http://www.annals.org/, and Epidemiology at http://www.epidem.com/). Information on the STROBE Initiative is available at www.strobe-statement.org.

For peer review only

BMJ Open

An evaluation of prescribing trends and patterns of claims within the Preferred Drugs Initiative in Ireland (2011-2016): an interrupted time-series study

Journal:	*BMJ Open*
Manuscript ID	bmjopen-2017-019315.R1
Article Type:	Research
Date Submitted by the Author:	24-Oct-2017
Complete List of Authors:	McDowell, Ronald; Royal College of Surgeons in Ireland, HRB Centre for Primary Care Research<br>Bennett, Kathleen; Royal College of Surgeons in Ireland, Population Health Sciences<br>Moriarty, Frank; Royal College of Surgeons in Ireland, HRB Centre for Primary Care Research, Department of General Practice<br>Clarke, Sarah; Health Service Executive Medicines Management Programme<br>Barry, Michael; National Centre for Pharmacoeconomics, Trinity Centre for Health Sciences<br>Fahey, Tom; Royal College of Surgeons in Ireland, Department of General Practice
**Primary Subject Heading**:	Epidemiology
Secondary Subject Heading:	General practice / Family practice, Health policy, Public health
Keywords:	Ireland, physician prescribing patterns, interrupted time series

SCHOLARONE™
Manuscripts

An evaluation of prescribing trends and patterns of claims within the Preferred Drugs Initiative in Ireland (2011-2016): an interrupted time-series study

McDowell RD[1], Bennett K[2], Moriarty F[1], Clarke S[3], Barry M[4], Fahey T[1]

[1] HRB Centre for Primary Care Research,
Department of General Practice,
Royal College of Surgeons in Ireland Medical School,
123 St Stephens Green,
Dublin 2

[2] Division of Population Health Sciences,
Royal College of Surgeons in Ireland,
Beaux Lane House,
Mercer Street Lower,
Dublin 2

[3] Health Service Executive Medicines Management Programme,
Trinity Centre for Health Sciences,
St. James's Hospital,
Dublin 8

[4] National Centre for Pharmacoeconomics,
Trinity Centre for Health Sciences,
St. James's Hospital,
Dublin 8

Correspondence to:

Ronald McDowell
Email: ronaldmcdowell@rcsi.ie

Word count: 4,031

Keywords: Ireland, physician prescribing patterns, interrupted time series

For peer review only

Abstract

Objective: To examine the impact of the Preferred Drugs Initiative (PDI), an Irish health policy aimed at enhancing evidence-based cost-effective prescribing, on prescribing trends and the cost of prescription medicines across seven medication classes.

Design: Retrospective repeated cross-sectional study spanning the years 2011 to 2016.

Setting: Health Service Executive Primary Care Reimbursement Scheme pharmacy claims data for General Medical Services (GMS) patients, approximately 40% of the Irish population.

Participants: Adults aged ≥18 years between 2011 to 2016 eligible for the GMS scheme.

Primary and secondary outcomes: The percentage of PDI medications within each drug class per calendar quarter. Linear regression was used to model prescribing of the preferred drug within each medication group and to assess the impact of PDI guidelines and other relevant changes in prescribing practice. Savings in drug expenditure were estimated.

Results: Between 2011 and 2016 around one quarter (23.59%) of all medications were for single-agent drugs licensed in the seven drug classes. There was a small increase in the percentage of PDI drugs, increasing from 4.64% of all medications in 2011 to 4.76% in 2016 ($p<0.001$). The percentage of preferred drugs within each drug class was significantly higher immediately following publication of the guidelines for all classes except urology, with the largest increases noted for lansoprazole (1.21%, 95%CI: 0.84% to 1.57%, $p<0.001$) and venlafaxine (0.71%, 95%CI: 0.15% to 1.27%), $p=0.02$). Trends in prescribing of the preferred drugs between PDI guidelines and the end of 2016 varied between drug classes. Total cost savings between 2013 and 2016 were estimated to be €2.7million.

Conclusion: There has been a small increase in prescribing of PDI drugs in response to prescribing guidelines, with inconsistent changes observed across therapeutic classes. These findings are relevant where health services are seeking to develop more active prescribing interventions aimed at changing prescribing practice.

For peer review only - http://bmjopen.bmj.com/site/about/guidelines.xhtml

Strengths and limitations of this study

- PCRS data covers pharmacy claims for prescriptions issued to General Medical Services (GMS) Scheme eligible patients (around 40% of the Irish population)
- Methods used are appropriate given the phased introduction of the preferred drug guidelines
- GMS patients over-represent older adults and those in receipt of social welfare
- Results based on aggregated data give an overview of the Preferred Drugs Initiative in its early years but require further detailed analysis to examine prescriber and patient heterogeneity.

For peer review only

Background

The Health Service Executive (HSE) in Ireland spent €1.05 billion in 2015 reimbursing pharmacists for the cost of prescription items issued to General Medical Services (GMS) eligible patients via the Primary Care Reimbursement Scheme (PCRS).(1) This is the largest community drug scheme in Ireland, providing access to free or minimal cost health care for patients whose household income falls below the eligibility threshold specified by the Irish Government, as well as the majority of people aged ≥70 years (approximately 95%) where a higher income threshold applies. Currently GMS eligible patients in Ireland have their prescription charges paid directly by the State, with a patient-levy of €2.50 for each item dispensed, up to a maximum of €25 per month. Historically Ireland has spent as much as 50% above the EU average per capita on drugs for a variety of reasons, such as low levels of use of generic medications and higher negotiated prices with pharmaceutical companies for both patented and generic drugs.(2, 3)

Against the background of an ageing population (4), the economic downturn of 2008 and rising drug costs the HSE established the Medicines Management Programme (MMP) in 2013. The MMP has undertaken a number of initiatives aimed at enhancing evidence-based and cost-effective prescribing (5), one of which is the Preferred Drugs Initiative (PDI). The PDI recommends a single 'preferred drug' within a therapeutic drug class as the prescriber's drug of first choice. Factors considered when selecting the preferred drug include clinical efficacy, ease of administration, the possibility of side effects or interactions with other drugs, cost, and national and international clinical guidelines. Recommendations for preferred drugs are made on an ongoing basis, with the findings disseminated through the publication of prescribing guidelines and GP meetings. The regulations covering generic substitution of branded medications are separate to the PDI guidelines, with generic substitution of drugs implemented where possible unless there are clinical reasons for prescribing the branded medication. The issuing of preferred drugs is voluntary and no incentives are given to prescribers to issue the preferred drug instead of others from within the same therapeutic drug class, with the patient levy remaining unaltered irrespective of preferred- or non-preferred drug status. Although the preferred drug may not necessarily be the least expensive licensed medication within each drug class, it has been estimated that increased provision of the preferred drugs could save the HSE €15 million per year.(5)

For peer review only - http://bmjopen.bmj.com/site/about/guidelines.xhtml

As of September 2016 reports detailing the rationale behind the choice of the preferred drugs have been published for the first ten therapeutic drug classes covered by the Initiative.(6) These are proton pump inhibitors (PPIs), statins, angiotensin-converting enzyme (ACE) inhibitors, angiotensin-II receptor blockers (ARBs), serotonin noradrenaline reuptake inhibitors (SNRIs), selective serotonin reuptake inhibitors (SSRIs), medications for treating urological conditions (urinary incontinence, frequency and overactive bladder), oral anticoagulants for stroke prevention in patients with non-valvular atrial fibrillation, beta-blockers and calcium channel blockers. There has been no evaluation of changes in prescribing following the introduction of the PDI to date. The aims of this paper are to: (i) examine the trends and patterns of pharmacy claims for seven PDI drug classes among eligible adult GMS patients in Ireland between 2011 and 2016; (ii) assess the impact of the PDI recommendations over time using segmented regression analysis; and (iii) estimate the cost savings due to the PDI during these years.

For peer review only - http://bmjopen.bmj.com/site/about/guidelines.xhtml

Methods

The STrengthening the Reporting of Observational Studies in Epidemiology (STROBE) guidelines were used in the reporting of this study (7).

Data

HSE-PCRS monthly pharmacy claims were analysed from 2011 to 2016 (8). This study period provided an average of three years of claims data both before and after the PDI across the seven drug classes considered. The data includes all pharmacy claims made for GMS patients and for which the cost of the claim has been reimbursed to community pharmacies by the HSE.

Preferred Drugs Initiative

The first seven medication classes covered by the PDI are considered in this paper. The preferred drugs in each of these classes were lansoprazole (PPIs), simvastatin (statins), ramipril (ACE inhibitors), candesartan (ARBs), venlafaxine (SNRIs), citalopram (SSRIs) and extended release (ER) tolterodine (urology medications). Guidelines for beta-blockers and calcium channel blockers were introduced in September 2016. Prescriptions issued to children (those under 18 years), hospital emergency items, out-of-hours prescriptions and items not considered medications (such as medical devices and dressings) were excluded; the PDI is primarily aimed at the treatment of adults in the general population.

Analytical methods/approach

Descriptive statistics were used to summarise relevant medications from the HSE-PCRS database and the classes of PDI drugs. Only single-agent drugs are considered in this paper, as this is the primary focus of the PDI.

The time-scale used for the analyses of time series depends on the research question of interest (9). Calendar quarters (January-March, April-June, July-September, October-December) were used to aggregate the data consistent with other analyses of prescribing data using interrupted time series (10-12). The use of calendar quarters was deemed clinically appropriate: changes in prescribing patterns tend to be gradual and guidelines are not necessarily disseminated or actioned on the first day of each calendar month. Furthermore Irish GMS eligible patients in receipt of prescription medication can receive three-months' worth of repeat prescriptions per consultation with their GP. For each therapeutic drug class a linear

For peer review only - http://bmjopen.bmj.com/site/about/guidelines.xhtml

regression model was used to estimate the percentage of the preferred drug per drug class per calendar quarter between 2011 to 2016, allowing for any changes that might have taken place following issuing of guidelines or other changes in clinical practice. This is a commonly-used strategy for analysing interrupted time series.(13) For medicine groups where the only “interruption” considered was dissemination of PDI guidelines, the regression equations used had the form

$$p_{ij} \quad = (\beta_{oj} + \beta_{1j}x_{ij1} + \beta_{2j}x_{ij2} + \beta_{3j}x_{ij3}) + e_{ij} \qquad (i = 0, \ldots, 23)$$

where for each medicine group j $(j = 1, \ldots, 7)$

p_{ij} is the percentage of items of the preferred drug reimbursed at time (quarter) i

β_{oj} is the estimated percentage of items being preferred drugs at t=0 (Jan-Mar 2011),

β_{1j} is the estimated change in the percentage of items being preferred drugs immediately following guidelines (the “change of level”)

β_{2j} is the estimated change in the percentage of items being preferred drugs per calendar quarter (the “slope”) before the guidelines

β_{3j} is the estimated change in the percentage of items being preferred drugs per calendar quarter (the “slope”) post -guidelines

e_{ij} is the residual for calendar quarter i.

The x_{ijk} $(k = 1,2,3)$ were calculated from the data according to standard practice. (14)

More than one change of level can be incorporated into any interrupted time series where this is relevant to the research question (13, 15). It was not feasible to include changes in the price of drugs in these models given the large number of drugs considered. Across the drug classes all drugs were licensed and available in Ireland between 2011 and 2016, and all generics were licensed prior to the study period, the key exceptions being the licensing of generic duloxetine in April 2015 and the licensing of mirabegron in January 2013. These two events were incorporated into the analyses for SNRIs and urology medications respectively.

Examination of the autocorrelation and partial autocorrelation coefficients showed that there was significant residual autocorrelation between adjacent calendar quarters (but not between non-adjacent quarters) in each drug group, and this was incorporated into the models using Prais–Winsten regression (16). The potential for seasonal autocorrelation was also considered:

For peer review only - http://bmjopen.bmj.com/site/about/guidelines.xhtml

in this context seasonal autocorrelation would mean that a given medication within a drug class is on average more or less likely to be prescribed than other drugs in the same class by virtue of the time of year. The PDI guidelines do not refer to any such clinical considerations (6) and we additionally hypothesised that seasonal autocorrelation would not be of statistical significance. This hypothesis was tested for each drug class by comparing the regression models which included Fourier terms to account for seasonality (9) and models without the seasonality terms. For each drug class seasonal autocorrelation was not of statistical significance and the seasonality terms were removed on the grounds of parsimony.

The PDI guidelines were national guidelines and consequently no control groups were available with which to compare prescribing under the PDI. However, we constructed two reference groups using the drug classes beta-blockers and calcium channel blockers. These were drug classes for which PDI guidelines were launched in September 2016 (the preferred drugs being bisoprolol and amlodipine respectively) but for which no recommendations had been made when the PDI guidelines were launched for the other drug classes. Given that the earlier guidelines were launched within six months of each other, two additional models were fitted: one examining prescribing of bisoprolol as the preferred beta-blocker over the study period, allowing for potential changes in prescribing when guidelines for PPIs/statins (April 2013) and SNRIs/SSRIs (April 2014) were disseminated, and one model examining prescribing of amlodipine as the preferred calcium channel blocker, allowing for potential changes in prescribing when guidelines for ACE inhibitors/ARBs (Sept 2013) and urology medications (October 2014) were issued.

By coincidence rather than design issuing of guidelines for each medicine group occurred at the beginning of the calendar quarters listed above, with the exception of the guidelines for ACE inhibitors and ARBs. Sensitivity analyses were used to explore whether the results varied when the calendar quarters were constructed differently (March-May, June-August, September-November, December-February) for these groups. Given that the PDI guidelines were launched in phases, sensitivity analyses were also used to examine whether results were dependent on the length of time considered before and after guidelines.

The models above were used to estimate increases or decreases in costs for each drug group associated with the PDI. Where only one interruption to the time series was included in the model, the predicted number of preferred drug items from each class was compared with the

For peer review only - http://bmjopen.bmj.com/site/about/guidelines.xhtml

number which would have been issued had the trend in prescribing estimated before the guidelines continued i.e. the estimates of $\beta_{oj}(\widehat{\beta_{oJ}})$ and $\beta_{2j}(\widehat{\beta_{2J}})$ remained unchanged, $\widehat{\beta_{1J}}$ was constrained to be zero and the estimate of $\beta_{3j}(\widehat{\beta_{3J}})$ was set equal to $\widehat{\beta_{2J}}$. The difference in the number of preferred drug items under the two scenarios was multiplied by the average price of the preferred drug, calculated across all reimbursements between dissemination of the guidelines and the end of 2016. The difference in the number of non-preferred drug items was multiplied by a weighted average of the price of all other drugs from within the medicine class, weighted according to the overall distribution of these items between issuing of the guidelines and the end of December 2016. These two costs were combined to give an overall cost differential. The process was extended analogously to include multiple interruptions as appropriate.

All analyses were conducted using Stata 14.0SE.(17) Results were held to be significant if they referred to statistical significance on a two-sided design-based test evaluated at the 0.05% level.

For peer review only

For peer review only - http://bmjopen.bmj.com/site/about/guidelines.xhtml

Results

Descriptive statistics

A total of 336,535,263 prescription items for medications were reimbursed by 4,465 PCRS prescribers for 1,919,681 GMS adults aged 18 years and over between 2011 and 2016. The median number of items reimbursed per GMS patient was 63 (Interquartile Range (IQR) 13 to 246) with a median total cost per patient of €905.75 (IQR €170.25 to €9,726.93). Approximately 55 million items were reimbursed per year, with the number of items peaking slightly in 2012 and 2013. During the six-year period 48.8 million (19.86%) prescription items were for the single-agent medicines licensed across the seven therapeutic drug classes considered. The drug classes most commonly prescribed to GMS patients were statins (5.93% of all items) and PPIs (5.63%), with the least common being SNRIs (0.99%) and drugs for treating urological conditions (0.67%). The descriptive statistics for each PDI medication class over the six-year period are outlined in Table 1.

The percentage of items relating to the seven drug classes increased slightly from 19.57% in 2011 to 20.04% in 2016, with small changes observed in the volume of prescriptions issued per each PDI medicine group over this time. More detailed breakdowns of PDI medicine groups per calendar year and quarter are given in Appendix Tables A1 & A2 and Figure A1.

Preferred Drugs Initiative

Within the seven PDI drug classes considered, 23.59% of all prescription items were for the named preferred drugs. However, there was considerable variation between PDI drug classes both in terms of ranking and percentage coverage of the preferred drug (see Table 1). The most commonly prescribed preferred drug within the relevant drug class was venlafaxine, which comprised 70.99% of all SNRI prescriptions. This was followed by ramipril (53.41% of all single-agent ACE inhibitors), ER tolterodine (25.79% of urology items), lansoprazole (24.14% of PPIs), citalopram (19.77% of SSRIs), candesartan (10.78% of all single-agent ARBs) and simvastatin (6.59% of all single-agent statins). The ranking of the preferred drugs within classes varied from first (ACE inhibitors and SNRIs), to second-last (statins). There was a small but statistically significant increase over time in the percentage of all medications which were for the PDI drugs, increasing from 4.64% in 2011 to 4.76% in 2016 ($p<0.001$).

Impact of clinical guidelines

For peer review only - http://bmjopen.bmj.com/site/about/guidelines.xhtml

Comparing prescribing patterns within each medication class in the three months pre-and post-publication of the PDI guidelines there was a small increase in the proportion of preferred drugs in four drug classes (PPIs (p<0.001), statins (p<0.001), ACE inhibitors (p<0.001) and SNRIs (p=0.08)), little change in two other drugs classes (ARBs (p=0.76) and SSRIs (p=0.37)), and a reduction in percentage terms in prescribing of the PDI agent ER tolterodine (p<0.001) (Table 1). Two preferred drugs, citalopram and ER tolterodine, were ranked lower within their respective classes between issuing of the guidelines and the end of 2016 than before. Figure 1 illustrates the secular trends for preferred drugs across the PDI categories by calendar quarters between 2011 and 2016: plots of the actual percentage of preferred drug items within each drug group between 2011 and 2016 are given in Appendix Figure A2.

Segmented linear regression showed changes over time in the prescribing of all preferred drugs (Table 2). For three medicine groups, there was significant evidence of an increase in the percentage of preferred drug items in the quarter immediately following issuing of the guidelines (lansoprazole (1.21%, 95%CI: 0.84% to 1.57%, p<0.001); venlafaxine (0.71%, 95%CI: 0.15% to 1.27%, p<0.001); simvastatin (0.30%, 95%CI: 0.1% to 0.5%, p=0.01)) and small increases in prescribing of the preferred drug in subsequent quarters. The percentage of SNRI medications which were venlafaxine did not change significantly immediately following the licensing of generic duloxetine in April 2015 (p=0.76) or in subsequent quarters (p=0.34). For both candesartan and citalopram, for which prescribing within their PDI drug classes was in decline prior to the guidelines being issued, prescribing increased immediately following the PDI guidelines (candesartan (0.15%, 95%CI: 0.02 to 0.29, p=0.03); citalopram (OR 0.30%, 95%CI: 0.12% to 0.47%, p=0.002)) but did not continue to increase significantly in subsequent quarters. Indeed prescribing of citalopram resumed in July 2014, although the decline was less steep than before the guidelines (p<0.001). For the other two medicine groups (ACE inhibitors and urology items), there was no notable impact of the PDI on prescribing of the preferred drugs. No statistically significant changes were observed in the prescribing of ER tolterodine immediately following the licensing of mirabegron in January 2013 (p=0.52) or the PDI guidelines in October 2014 (p=0.82), although the rate of decline in prescribing of ER tolterodine was lower following the PDI guidelines than between the licensing of mirabegron and dissemination of the PDI guidelines (p<0.001).

For peer review only - http://bmjopen.bmj.com/site/about/guidelines.xhtml

Sensitivity analyses showed that the results were materially unaffected when the calendar quarters used for analyses of ACE inhibitors and ARBs varied or when the length of time studied before and after the guidelines was changed (Appendix Tables A3, A4).

Reference groups

Beta-blockers and calcium channel blockers accounted for 3.58% (n=12,056,378) and 2.30% (n=7,753,755) of single-agent medications for GMS patients between 2011 and 2016, with the most commonly prescribed medications being bisoprolol (56.83% of all single-agent beta-blockers (n=6,852,022)) and amlodipine (64.70% of all single-agent calcium channel blockers (n=5,016,348)), both of which were selected as preferred drugs in September 2016. There was a steady increase in prescribing of bisoprolol as the beta-blocker of choice and a consistent fall in prescribing of amlodipine within the calcium channel blocker medications over the study period. Effects associated with dissemination of the PDI guidelines for the other drug groups were non-significant at the 5% level (Table 3). See Figure 2 for plots of the estimated percentage of preferred drug items within each therapeutic drug class between 2011 and 2016.

Cost savings

Overall, the cost savings after introduction of the PDI amounted to €2,671k across all seven PDI drug classes (Table 2). The savings associated with changes in prescribing following issuing of guidelines for the seven drug classes were estimated to be €123k in 2013, €396k in 2014, €837k in 2015 and €1,314k in 2016. There were savings in each group, even though changes in dispensed medications were often minimal. The greatest impact was on the amount spent on SNRIs, with an estimated saving of €1,291k between 2014-2016. This is due to the much higher cost of the non-preferred drug duloxetine to the preferred drug venlafaxine. Other groups where the savings were marked were for the two larger volume groups where the guidelines had first been issued- PPIs saving €618k and statins saving €363k. For medicine groups where prescribing of the preferred drug was in decline before guidelines were issued, even the small short-term changes in prescribing translated into some savings. The smallest cost savings were in the prescribing of ramipril and ER tolterodine, due to the lack of change in prescribing trends observed within these groups between 2011 and 2016. The combined savings in the reference groups, had the prescribing patterns observed prior to the PDI guidelines remained unchanged, was an estimated €17k.

For peer review only - http://bmjopen.bmj.com/site/about/guidelines.xhtml

Discussion

Principal findings

The seven drug classes considered that form part of the PDI accounted for approximately 20% of all medications reimbursed by the PCRS between 2011 and 2016. Changes in prescribing observed over the study period varied by PDI drug class, with substantial differences in the ranking order and quantity of preferred drug prescribed. Overall, the impact of the PDI guidance was limited, with an inconsistent pattern observed across all therapeutic drug classes, and only a small increase (0.13%) in the percentage of preferred drugs issued overall between 2011 and 2016. Across the PDI drug classes some differences emerged: in the first group of PDI drugs there were increases in prescribing of the preferred drug immediately following issuing of the guidelines and continued though small increases subsequently (PPIs, Statins and SNRIs); in the second group of PDI drugs (SSRIs and ARBs) there was a temporary increase in prescribing of the preferred drug just after the guidelines were issued; lastly, in the third group of PDI drugs (ACE and urology), there appeared to be little or no impact of clinical guidance. The reasons for such diversity are not known. ACE inhibitors are relatively inexpensive and this may account, in part, for the trend in ramipril prescribing remaining unaltered. Although mirabegron has become the most commonly prescribed urology item since its launch in 2013, prescribing of ER Tolterodine was in decline prior to this time.

Context of other studies

PDI guidelines to date have been disseminated to prescribers mainly through correspondence and GP meetings. The literature shows that educational programmes and publication of guidelines in themselves tend to have little effect on influencing prescribing practice, and that these need to be enhanced with other strategies.(18) In a systematic review of 79 studies examining interventions which changed doctor prescribing behaviour, the most effective interventions were patient-mediated interventions, outreach, audit and feedback, and reminders.(19) In a study of changes in the use of losartan versus other single ARBs in Sweden investigators concluded that multiple and intensive demand-side measures are needed to change physician prescribing habits.(20) Other strategies which have been found to be helpful include direct involvement of the community pharmacist and face-to-face engagement from those seeking to encourage change with the prescriber .(21) Technological advances, such as alerts and prompts when issuing a drug may also prove useful. (22)

For peer review only - http://bmjopen.bmj.com/site/about/guidelines.xhtml

Any excess expenditure incurred through the issuing of non-preferred drugs to GMS patients is met directly by the HSE and not by the patient. Options which could reduce such expenditure include reducing choice for either patient or prescriber. It has been suggested that because prescribers can develop expertise of only a certain number of drugs, more restrictive formularies may also provide benefits to quality of prescribing (23, 24). In Sweden, the introduction of the 'Wise List', an evidence-based formulary of essential medicines, increased adherence to guideline recommendations in primary care from 80% to 90% and reduced variation in prescribing (25). The introduction of co-payments, where the patient has to pay the difference between the price of the preferred drug and their chosen alternative, has the potential to be a considerable driver of change. Australia operates a therapeutic brand premium scheme, whereby a co-payment is required from patients when a prescriber has issued a drug within a drug class that is priced above the benchmark for drugs in that group.(26) While dramatic changes in co-payments may result in more patients switching to preferred agents (such as statins, ACE inhibitors and PPIs), they may also increase the risk of patients stopping their medication or becoming non-adherent (27, 28). Recent work has shown the drivers of drug expenditure in high income countries varies substantially, with several other factors aside from physician prescribing behavior and patient preference determining national drug expenditure.(29)

<u>Strengths and limitations</u>

There are a number of strengths to this study. Our prescription sample is large and generalisable: PCRS data covers the entire GMS population of Ireland (around 40% of individuals). Despite the guidelines being introduced incrementally, the results were invariant to the time periods studied pre- and post-publication of clinical guidelines. However, there are limitations to the study. GMS patients are weighted towards older adults and those socially and financially disadvantaged and so the results may not be reflective of the entire population in receipt of prescription medication. There is no way of knowing whether prescribers approached patients with regards to changes in their medication and/or whether these approaches were successful. Patient-specific factors may mean that issuing of the preferred drug may not have been appropriate or possible. Neither prescribers nor patients are homogeneous entities and considerable variation may exist within both.

For peer review only - http://bmjopen.bmj.com/site/about/guidelines.xhtml

Policy implications and future research

The PDI has been developed to encourage evidence-based, cost-effective prescribing, but in view of the limited changes to date has delivered only a small amount of cost savings in terms of the money spent on these prescription items. If cost savings are to be maximised, the energies need to focus on medicine groups which are large volume (e.g. PPIs and statins) and/or where there is considerable variation between the least and most expensive licensed medications in that group (e.g. SNRIs). To enhance the impact of the PDI, multi-faceted interventions appear most likely to succeed. Financial incentives to prescribers may be one possible component of such interventions, as operated in Irish primary care for a time in the 1990's (30), however any incentives for PDI drugs need to be aligned with professional values of prescriber, and be mindful of personal preferences of patients taking long-term medication (31-33). The effectiveness of such interventions is important to consider and although this has generally been evaluated using observational methods, experimental approaches may also be feasible.

Findings from this evaluation of the PDI in Ireland may be of interest to other countries which have implemented (e.g. Australia) or are considering preferred drug schemes or any intervention aimed at changing prescribing or clinical practice. The heterogeneity within our results illustrates that interventions developed using the same methodological framework may not necessarily yield comparable results even when launched concurrently.

Conclusions

Since the introduction of the PDI in 2013, there have been some cost savings across the PDI drug classes. However, more intensive implementation is needed before the PDI delivers the estimated €15million per year cost saving that was anticipated. Multifaceted interventions will be required to enhance the coverage and impact of the PDI so that these benefits can be realised.

For peer review only - http://bmjopen.bmj.com/site/about/guidelines.xhtml

Acknowledgements: Thanks to the Health Services Executive Primary Care Reimbursement Scheme (HSE-PCRS) for the use of pharmacy claims database.

Contributors: RMcD drafted and planned all aspects of study design, cleaned and prepared data for analysis, conducted the statistical analyses and conducted a preliminary overview of the literature. KB prepared the monthly PCRS claims downloads and gave significant methodological guidance on the analysis strategy. FM provided guidance on pharmaceutical matters and contributed to the discussion on context, policy implications and future research. SC and MB facilitated access to the claims data with the PCRS, gave detailed information on roll-out and implementation of the Preferred Drugs Initiative, and contributed to interpretation of the results within the wider context of prescribing in Ireland. TF generated the research question and commented on the conduct, analysis and write-up of the paper. All authors read and approved submission of the paper.

Funding: The primary author was funded by the Health Research Board (HRB) of Ireland under the HRB Centre for Primary Care Phase 2 Funding award, grant HRC/2014/1. KB funded by HRB under grant RL-15-1579

Competing interests: None declared.

Data-sharing statement: No additional data are available.

For peer review only

References

1. Service HSEPCR. Primary Care reimbursement Service Statistical Analysis of Claims and Payments 2015. Available from: http://www.hse.ie/eng/staff/PCRS/PCRS_Publications/PCRS-statistical-analysis-of-claims-and-payments-2015.pdf.
2. Children HotOJCoHa. Report on the Cost of Prescription Drugs in Ireland 2015. 2015.
3. Brick A GP, Nolan A. Ireland: Pharmaceutical Prices, Prescribing Practices and Usage of Generics in a Comparative Context. Dublin: ESRI, 2013.
4. Layte R. (ed) BM, Bennett K, Brick A, Morgenroth E., Normand C, O'Reilly J, Thomas S, Tilson L, Wiley M, Wren M.-A. Demographic Change on the Demand for and Delivery of Health Care in Ireland. Dublin: ESRI, 2009.
5. Programme MM. Welcome to the Medicines Management Programme 2016. Available from: http://www.hse.ie/eng/about/Who/clinical/natclinprog/medicinemanagementprogramme/yourmedicines/.
6. Programme MM. Evaluation Reports. 2017.
7. von Elm E, Altman DG, Egger M, Pocock SJ, Gotzsche PC, Vandenbroucke JP, et al. The Strengthening the Reporting of Observational Studies in Epidemiology (STROBE) statement: guidelines for reporting observational studies. Journal of clinical epidemiology. 2008;61(4):344-9.
8. Sinnott SJ, Bennett K, Cahir C. Pharmacoepidemiology resources in Ireland-an introduction to pharmacy claims data. European journal of clinical pharmacology. 2017.
9. Bhaskaran K, Gasparrini A, Hajat S, Smeeth L, Armstrong B. Time series regression studies in environmental epidemiology. International journal of epidemiology. 2013;42(4):1187-95.
10. Rector TS, Finch MD, Danzon PM, Pauly MV, Manda BS. Effect of tiered prescription copayments on the use of preferred brand medications. Medical care. 2003;41(3):398-406.
11. Lopatto J, Keith SW, Del Canale S, Templin M, Maio V. Evaluating sustained quality improvements: long-term effectiveness of a physician-focused intervention to reduce potentially inappropriate medication prescribing in an older population. Journal of Clinical Pharmacy & Therapeutics. 2014;39(3):266-71.
12. Penfold RB, Zhang F. Use of interrupted time series analysis in evaluating health care quality improvements. Academic pediatrics. 2013;13(6 Suppl):S38-44.
13. Wagner AK, Soumerai SB, Zhang F, Ross-Degnan D. Segmented regression analysis of interrupted time series studies in medication use research. Journal of clinical pharmacy and therapeutics. 2002;27(4):299-309.
14. Education UIfDRa. How can I run a piecewise regression in Stata? 2017. Available from: https://stats.idre.ucla.edu/stata/faq/how-can-i-run-a-piecewise-regression-in-stata/.
15. Bernal J, Cummins, S, Gasparrini, A. Interrupted time series regression for the evaluation of public health interventions: a tutorial. International journal of epidemiology. 2017;46(1):384-55.
16. Prais SJ, and C. B. Winsten. Trend estimators and serial correlation: Working paper 383, Cowles Commission; 1954. Available from: http://cowles.econ.yale.edu/P/ccdp/st/s-0383.pdf.
17. StataCorp. Stata Statistical Software. Texas: StataCorp; 2015.
18. Davis D, O'Brien MA, Freemantle N, Wolf FM, Mazmanian P, Taylor-Vaisey A. Impact of formal continuing medical education: do conferences, workshops, rounds, and other traditional continuing education activities change physician behavior or health care outcomes? Jama. 1999;282(9):867-74.
19. Gill PS, Makela M, Vermeulen KM, Freemantle N, Ryan G, Bond C, et al. Changing doctor prescribing behaviour. Pharmacy world & science : PWS. 1999;21(4):158-67.
20. Godman B, Wettermark B, Miranda J, Bennie M, Martin A, Malmstrom RE. Influence of multiple initiatives in Sweden to enhance ARB prescribing efficiency following generic losartan; findings and implications for other countries. International journal of clinical practice. 2013;67(9):853-62.

For peer review only - http://bmjopen.bmj.com/site/about/guidelines.xhtml

21. Soumerai SB, Avorn J. Principles of educational outreach ('academic detailing') to improve clinical decision making. Jama. 1990;263(4):549-56.
22. Kuperman GJ, Bobb A, Payne TH, Avery AJ, Gandhi TK, Burns G, et al. Medication-related clinical decision support in computerized provider order entry systems: a review. Journal of the American Medical Informatics Association : JAMIA. 2007;14(1):29-40.
23. Persaud N, Ahmad H. Canadian list of essential medications: Potential and uncertainties. Canadian family physician Medecin de famille canadien. 2017;63(4):266-8.
24. Wettermark B, Pehrsson A, Jinnerot D, Bergman U. Drug utilisation 90% profiles--a useful tool for quality assessment of prescribing in primary health care in Stockholm. Pharmacoepidemiology and drug safety. 2003;12(6):499-510.
25. Eriksen J, Gustafsson LL, Ateva K, Bastholm-Rahmner P, Ovesjo ML, Jirlow M, et al. High adherence to the 'Wise List' treatment recommendations in Stockholm: a 15-year retrospective review of a multifaceted approach promoting rational use of medicines. BMJ open. 2017;7(4):e014345.
26. Health AGo. Therapeutic Group Premium Policy 2017. Available from: https://www.pbs.gov.au/browse/group-premium.
27. Sinnott SJ, Normand C, Byrne S, Woods N, Whelton H. Copayments for prescription medicines on a public health insurance scheme in Ireland. Pharmacoepidemiology and drug safety. 2016;25(6):695-704.
28. Huskamp HA, Deverka PA, Epstein AM, Epstein RS, McGuigan KA, Frank RG. The effect of incentive-based formularies on prescription-drug utilization and spending. The New England journal of medicine. 2003;349(23):2224-32.
29. Morgan SG, Leopold C, Wagner AK. Drivers of expenditure on primary care prescription drugs in 10 high-income countries with universal health coverage. CMAJ : Canadian Medical Association journal = journal de l'Association medicale canadienne. 2017;189(23):E794-E9.
30. Walley T, Murphy M, Codd M, Johnston Z, Quirke T. Effects of a monetary incentive on primary care prescribing in Ireland: changes in prescribing patterns in one health board 1990-1995. Pharmacoepidemiology and drug safety. 2000;9(7):591-8.
31. Roland M. Incentives must be closely aligned to professional values. Bmj. 2012;345:e5982.
32. Majumdar SR, Soumerai SB. Why most interventions to improve physician prescribing do not seem to work. CMAJ : Canadian Medical Association journal = journal de l'Association medicale canadienne. 2003;169(1):30-1.
33. Persaud N, Lee T, Ahmad H, Li W, Taglione MS, Rajakulasingam Y, et al. Protocol for a randomised controlled trial evaluating the effects of providing essential medicines at no charge: the Carefully seLected and Easily Accessible at No Charge Medicines (CLEAN Meds) trial. BMJ open. 2017;7(5):e015686.

For peer review only - http://bmjopen.bmj.com/site/about/guidelines.xhtml

Table 1:Summary of impact of Preferred Drugs Initiative (2011-2016)

Preferred drug class	PPI	Statin	ACE	ARB	SNRI	SSRI	urology	Total
Total no. items	18,939,282	19,944,634	8,837,006	5,171,204	3,345,307	8,348,567	2,239,263	336,535,263
% of all drugs	5.63%	5.93%	2.63%	1.54%	0.99%	2.48%	0.67%	19.86%
**Preferred drug**	**lansoprazole**	**simvastatin**	**ramipril**	**candesartan**	**venlafaxine**	**citalopram**	**ER tolterodine**	
**Total no. single-agent items**	4,571,751	1,313,389	4,719,996	557,622	1,155,600	1,650,520	577,540	
**% within class**	24.14%	6.59%	53.41%	10.78%	70.99%	19.77%	25.79%	
**Rank within class pre-PDI**	2/5	4/5	1/10	5/8	1/2	2/6	1/9	
**Rank within class post-PDI**	2/5	4/5	1/10	5/8	1/2	3/6	3/9	
**Absolute change in proportion of preferred drug items: first 3 months post-PDI v previous 3 months**	↑ +0.98% ($p<0.001$)	↑ +0.30% ($p<0.001$)	↑ 0.53% ($p<0.001$)	↓ -0.03% ($p=0.76$)	↑ 0.30% ($p=0.08$)	↓ -0.09% ($p=0.37$)	↓ -0.98% ($p<0.001$)	

PPI: proton pump inhibitor; ACE: angiotensin-converting enzyme (ACE) inhibitor; ARB: angiotensin-II receptor blocker; SNRI: serotonin and noradrenaline reuptake inhibitor ; SSRI: selective serotonin receptor antagonist; ER: Extended Release; PDI: Preferred Drugs Initiative

For peer review only

For peer review only - http://bmjopen.bmj.com/site/about/guidelines.xhtml

Table 2: Segmented regression analysis in relation to PDI guideline publication, class-specific changes and cost savings

Medicine group (Preferred drug)	Guidelines introduced	Percentage of preferred drug items: Jan-March 2011 (SE), 95% CI	Increase in % of preferred drug items per quarter post March 2011 (SE), 95%CI, p-value	Increase in % of preferred drug items Jan-Mar 2013 following licensing of mirabegron, (SE), 95%CI, p-value	Increase in % of preferred drug items per quarter post March 2013 (SE), 95%CI, p-value	Increase in % of preferred drug items quarter immediately following PDI guidelines, (SE), 95%CI, p-value	Increase in % of preferred drug items per quarter post PDI guidelines, (SE), 95%CI, p-value	Increase in % of preferred drug April-June 2015 following introduction of generic duloxetine, (SE), 95%CI, p-value	Increase in % of preferred drug items per quarter post June 2015 , (SE), 95%CI, p-value	Estimated savings between issuing of guidelines and Dec 2016 (€)
PPIs (lansoprazole)	April 2013	24.53 (0.47), (23.59,25.47)	-0.21 (0.05), (-0.32,-0.11), p=0.001	-	-	1.21 (0.18), (0.84,1.57), p<0.001	0.04 (0.04) (-0.03,0.12), p=0.25	-	-	618,158
Statins (simvastatin)	April 2013	5.94 (0.21), (5.50,6.38)	0.02 (0.03), (-0.04,0.07), p=0.54	-	-	0.30 (0.10), (0.10,0.50), p=0.01	0.07 (0.02), (0.03,0.10), p=0.002	-	-	363,194
ACEs (ramipril)	September 2013	49.14 (0.07), (48.99,49.28)	0.38 (0.01). (0.35,0.40), p<0.001	-	-	0.16 (0.07), (0.01,0.31), p=0.04	0.41 (0.01), (0.39,0.42), p<0.001	-	-	50,163
ARBs (candesartan)	September 2013	11.90 (0.08), (11.73,12.07)	-0.15 (0.01), (-0.17,-0.12), p<0.001	-	-	0.15 (0.06), (0.02,0.29), p=0.03	0.01 (0.01), (-0.01,0.03), p=0.46	-	-	132,625
SNRIs (venlafaxine)	April 2014	73.61 (0.44), (72.69,74.53)	-0.35 (0.05), (-0.46,-0.24), p<0.001	-	-	0.71 (0.27), (0.15,1.27), p=0.02	0.26 (0.13), (-0.02,0.55), p=0.07	-0.09 (0.30), (-0.73,0.54), p=0.76	-0.08 (0.09), (-0.10,0.26), p=0.34	1,291,160
SSRIs (citalopram)	April 2014	23.58 (0.13), (23.31,23.85)	-0.36 (0.01), (-0.39,-0.33), p<0.001	-	-	0.30 (0.08), (0.12,0.47), p=0.002	-0.23 (0.02), (-0.27,-0.19), p<0.001	-	-	169,493
urology (ER tolterodine)	October 2014	37.27 (0.27), (36.69,37.84)	-1.00 (0.05), (-1.11,-0.88), p<0.001	0.16 (0.24), (-0.35,0.66), p=0.52	-1.04 (0.06), (-1.17,-0.91), p<0.001	-0.06 (0.24), (-0.57,0.45), p=0.82	-0.63 (0.09), (-0.73,-0.52), p<0.001	-	-	46,695
**Total savings**										**2,671,447**

For peer review only

For peer review only - http://bmjopen.bmj.com/site/about/guidelines.xhtml

CI: Confidence Interval; SE: Standard Error; ER: Extended Release; PPI: proton pump inhibitor; ACE: angiotensin-converting enzyme (ACE) inhibitor; ARB: angiotensin-II receptor blocker; SNRI: serotonin and noradrenaline reuptake inhibitor ; SSRI: selective serotonin receptor antagonist; ER: Extended Release; PDI: Preferred Drugs Initiative

For peer review only

For peer review only - http://bmjopen.bmj.com/site/about/guidelines.xhtml

Table 3: Segmented regression analysis in relation to PDI guideline publication, reference groups

Medicine group (Preferred drug)	Guidelines introduced	Percentage of preferred drug items: Jan-March 2011 (SE), 95% CI	Increase in % of preferred drug items per quarter post March 2011 (SE), 95%CI, p-value	Increase in % of preferred drug items April-June 2013, (SE), 95%CI, p-value	Increase in % of preferred drug items per quarter post June 2013, (SE), 95%CI, p-value	Increase in % of preferred drug items Oct-Dec 2013, (SE), 95%CI, p-value	Increase in % of preferred drug items per quarter post Dec 2013, (SE), 95%CI, p-value	Increase in % of preferred drug items April-June 2014, (SE), 95%CI, p-value	Increase in % of preferred drug items per quarter post June 2014, (SE), 95%CI, p-value	Increase in % of preferred drug items Oct-Dec 2014, (SE), 95%CI, p-value	Increase in % of preferred drug items per quarter post Dec 2014, (SE), 95%CI, p-value
beta-blockers (bisoprolol)	September 2016	51.20 (0.03), (51.15,51.26)	0.53 (0.01), (0.52,0.54), p<0.001	-0.02 (0.05), (-0.13,0.09), p=0.71	0.50 (0.02), (0.45,0.54), p<0.001	-	-	-0.05 (0.06), (-0.18,0.08), p=0.44	0.41 (0.001), (0.40,0.42), p<0.001	-	-
calcium channel blockers (amlodipine)	September 2016	68.18 (0.03), (68.12,68.29)	-0.34 (0.01), (-0.35,-0.33), p<0.001	-	-	0.12 (0.06), (-0.001,0.23), p=0.06	-0.26 (0.02) (-0.31,-0.21), p<0.001	-	-	0.02 (0.07), (-0.13,0.17), p=0.76	-0.21 (0.01) (-0.22,-0.19), p<0.001

CI: Confidence Interval; SE: Standard Error; ER: Extended Release

For peer review only

For peer review only - http://bmjopen.bmj.com/site/about/guidelines.xhtml

Figure Legends

Figure 1: Distribution of preferred drug items by therapeutic drug class

Figure 2: Estimated percentage of preferred drugs by drug class: segmented regression models

For peer review only

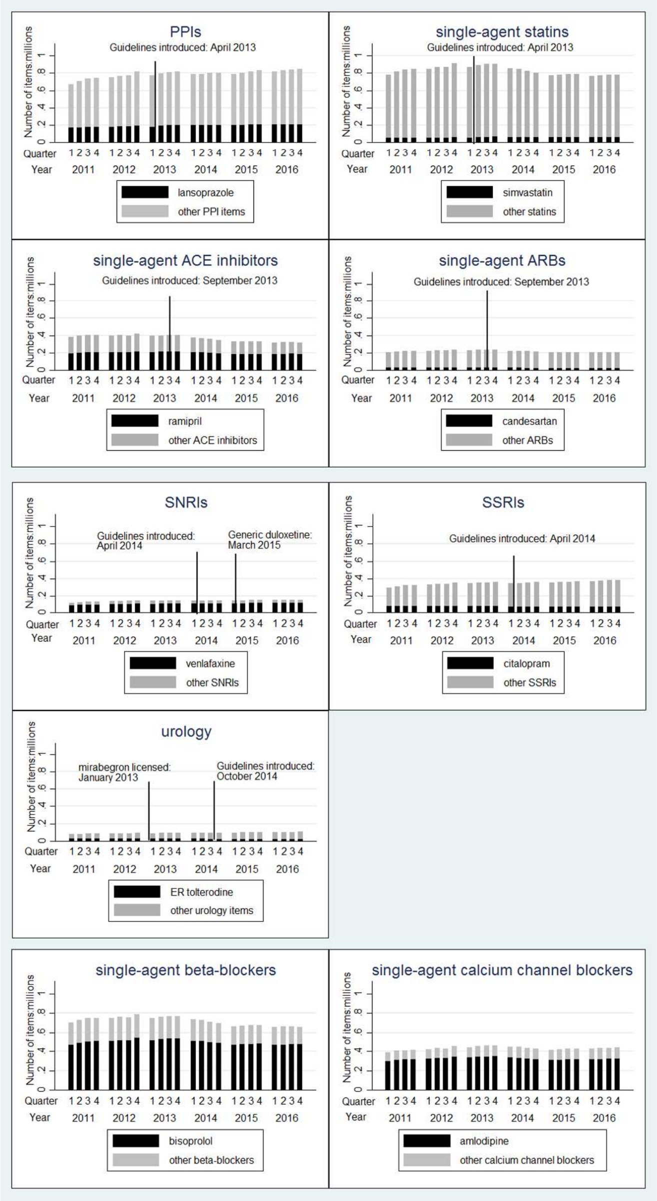

Figure 1: Distribution of preferred drug items by therapeutic drug class

162x297mm (300 x 300 DPI)

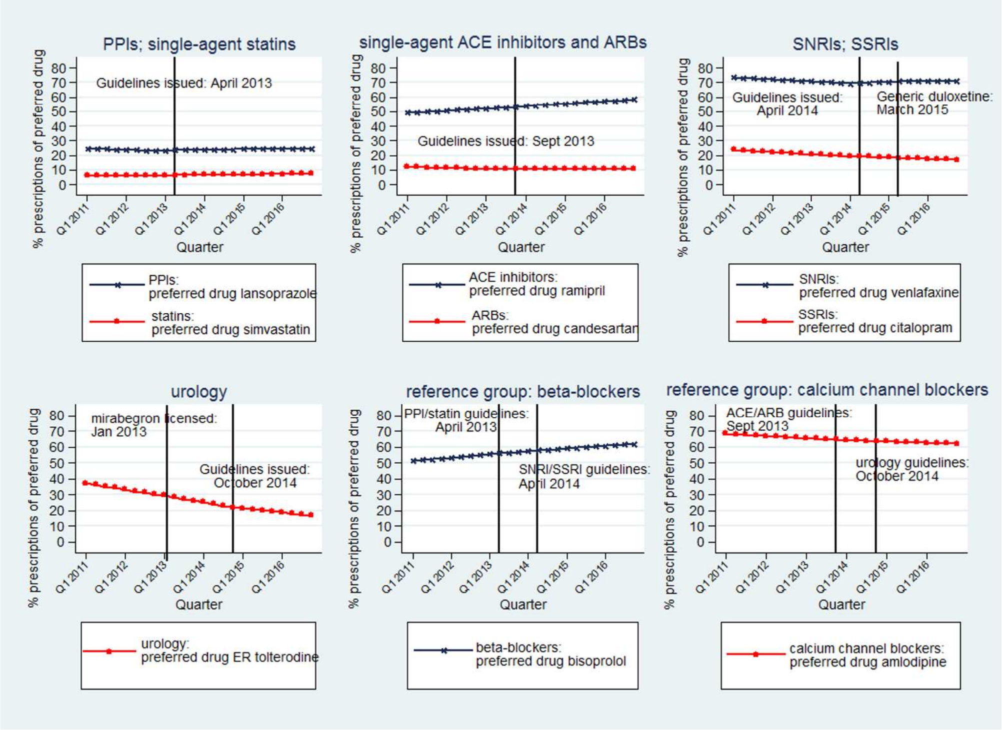

Figure 2: Estimated percentage of preferred drugs by drug class: segmented regression models

209x152mm (300 x 300 DPI)

Appendix

Table A1: Breakdown of PCRS reimbursed items: 2011-2016

Year	No. items issued	No. single-agent items issued across 7 drug classes*	% of items attributed to 7 drug classes*	No. items issued for preferred drugs	% preferred drug items within preferred drug classes	% preferred drug items across all prescriptions
**2011**	54,324,492	10,630,476	19.57%	2,520,986	23.71%	4.64%
**2012**	57,984,934	11,380,582	19.63%	2,641,897	23.21%	4.56%
**2013**	58,455,927	11,640,615	19.91%	2,708,855	23.27%	4.63%
**2014**	55,978,157	11,181,081	19.97%	2,655,422	23.75%	4.74%
**2015**	54,573,162	10,925,162	20.02%	2,610,926	23.90%	4.78%
**2016**	55,218,591	11,067,347	20.04%	2,627,631	23.74%	4.76%
**Total**	336,553,263	66,825,263	19.86%	15,765,717	23.59%	4.68%

***PPIs, Statins, ACEs, ARBs, SNRIs, SSRIs, Urology**

Table A2 Prevalence of PCRS reimbursed items by therapeutic drug class (single agent drugs)

Year	PPIs	statins	ACEs	ARBs	SNRIs	SSRIs	urology	Other	Total
**2011**	2,860,986 (5.27%)	3,286,352 (6.05%)	1,586,992 (2.92%)	849,807 (1.56%)	470,234 (0.87%)	1,247,643 (2.30%)	328,462 (0.60%)	43,694,016 (80.43%)	54,324,492 (100%)
**2012**	3,114,214 (5.37%)	3,501,257 (6.04%)	1,616,612 (2.79%)	899,594 (1.55%)	537,800 (0.93%)	1,355,921 (2.34%)	355,184 (0.61%)	46,604,352 (80.37%)	57,984,934 (100%)
**2013**	3,203,104 (5.48%)	3,582,112 (6.13%)	1,595,582 (2.73%)	920,851 (1.58%)	566,951 (0.97%)	1,404,466 (2.40%)	367,549 (0.63%)	46,815,312 (80.09%)	58,455,927 (100%)
**2014**	3,180,702 (5.68%)	3,339,227 (5.97%)	1,449,173 (2.59%)	867,567 (1.55%)	567,859 (1.01%)	1,399,724 (2.50%)	376,829 (0.67%)	44,797,076 (80.03%)	55,978,157 (100%)
**2015**	3,241,661 (5.94%)	3,129,117 (5.73%)	1,312,155 (2.40%)	816,250 (1.50%)	588,689 (1.08%)	1,441,270 (2.64%)	396,020 (0.73%)	43,648,000 (79.98%)	54,573,162 (100%)
**2016**	3,338,615 (6.05%)	3,106,569 (5.63%)	1,276,492 (2.31%)	817,135 (1.48%)	613,774 (1.11%)	1,499,543 (2.72%)	415,219 (0.75%)	44,151,244 (79.96%)	55,218,591 (100%)
**Total**	18,939,282 (5.63%)	19,944,634 (5.93%)	8,837,006 (2.63%)	5,171,204 (1.54%)	3,345,307 (0.99%)	8,348,567 (2.48%)	2,239,263 (0.67%)	269,710,000 (80.14%)	336,535,263 (100%)

Fig A1: Breakdown of PCRS reimbursed items by preferred drug status: 2011-2016

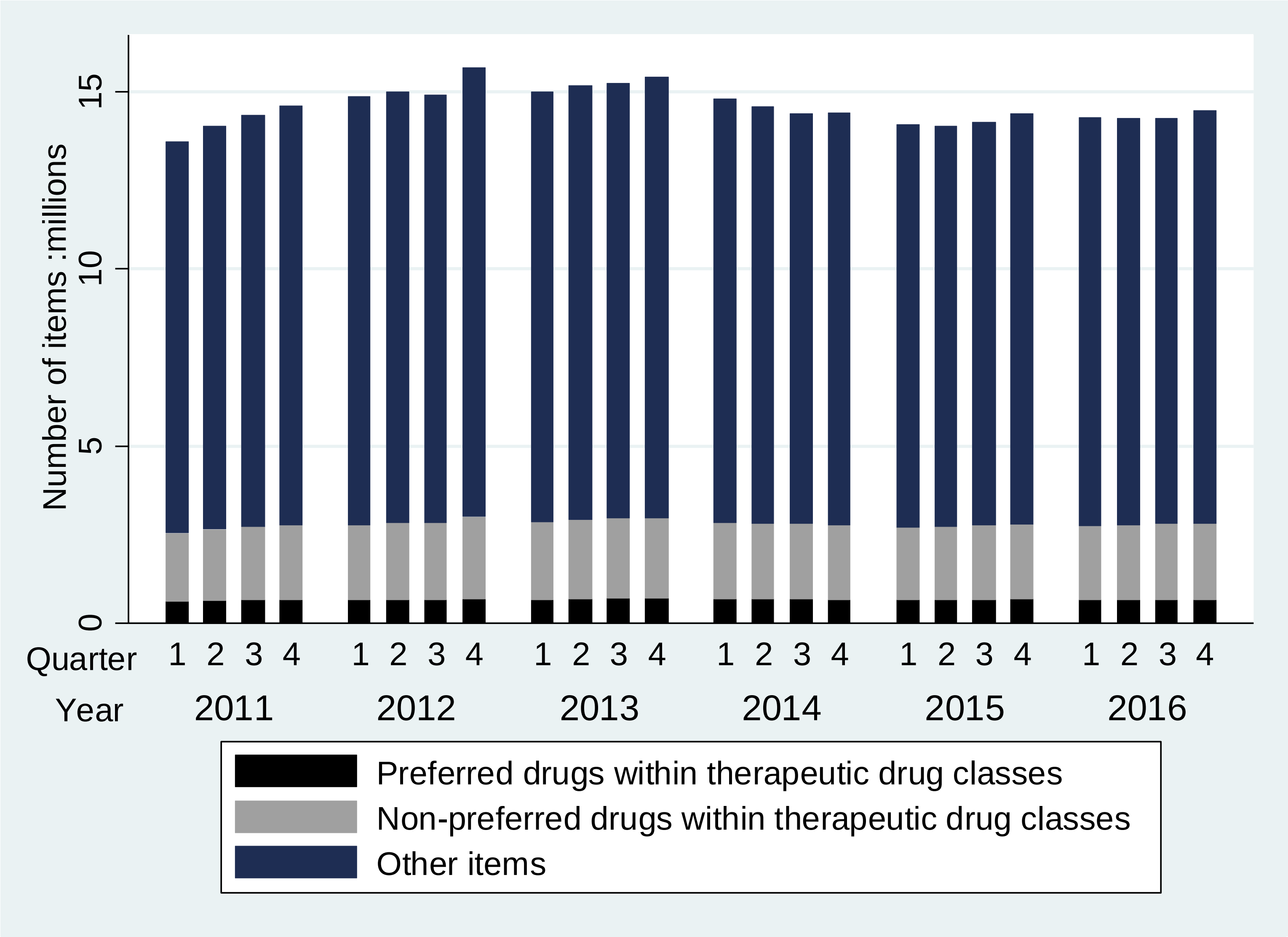

Fig A2: Observed percentage of preferred drugs by drug class

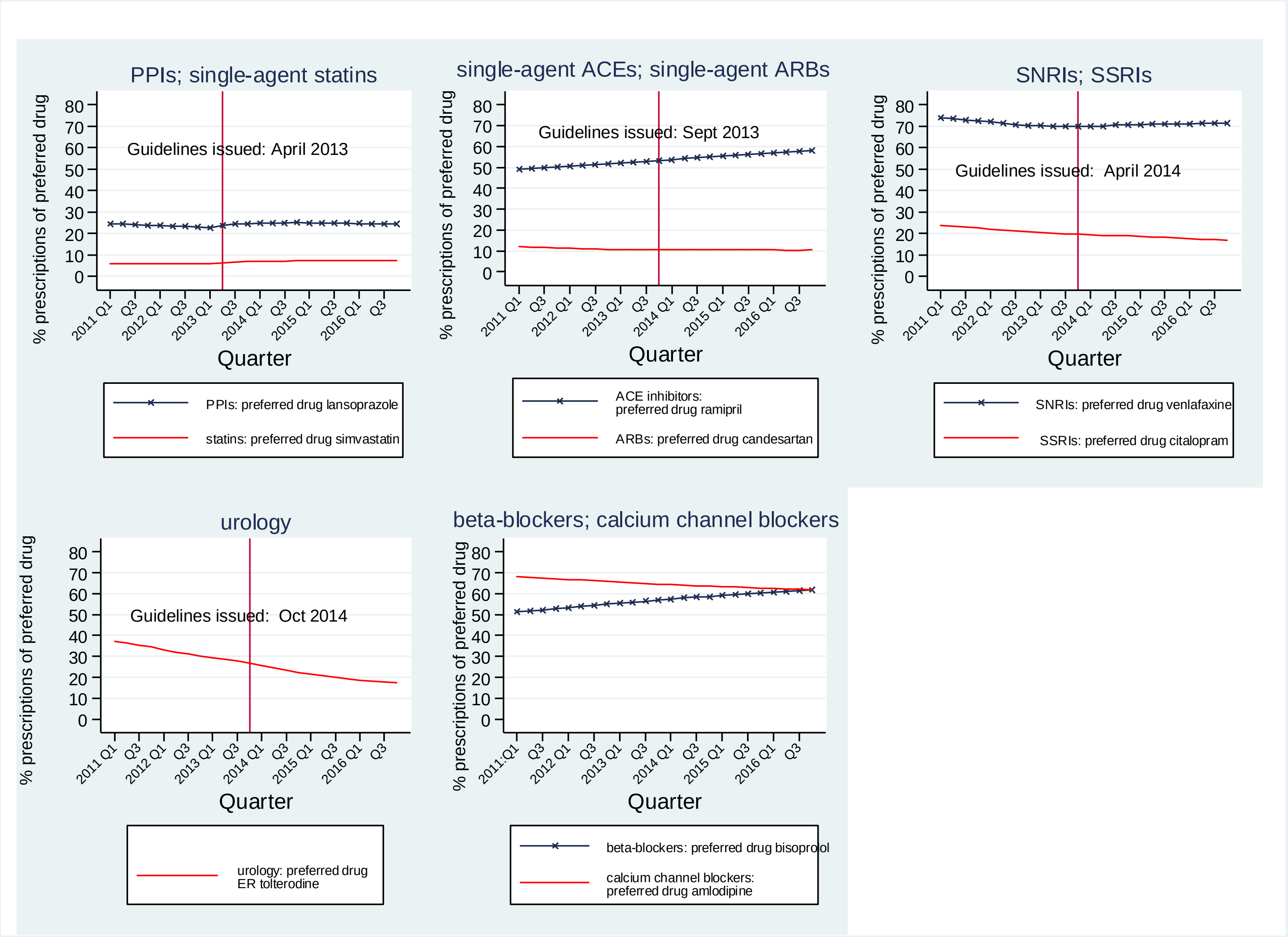

Table A3: Sensitivity analyses for segmented regression models

	Calendar quarters retained for analysis (Study Period)			
	**All available data: 9 quarters before guidelines, 15 quarters after guidelines, (Jan 11- Dec 16)**	**9 quarters before guidelines, 13 quarters after guidelines (Jan 11-Jun 16)**	**9 quarters before guidelines, 11 quarters after guidelines (Jan 11-Dec 15)**	**9 quarters before guidelines, 9 quarters after guidelines (Jan 11-Jun 15)**
**lansoprazole**				
Percentage of preferred drug items: beginning of study period (SE), 95% CI	24.53 (0.47), (23.59,25.47)	24.51 (0.40), (23.66,25.36)	24.47 (0.29), (23.85,25.09)	24.42 (0.19), (24.02,24.83)
Increase in % of preferred drug items per quarter following commencement of study period (SE), 95%CI, p-value	-0.21 (0.05), (-0.32,-0.11), p=0.001	-0.21 (0.05), (-0.32,-0.10), p=0.001	-0.21 (0.04), (-0.30,-0.11), p<0.001	-0.21 (0.04), (-0.28,-0.14), p<0.001
Increase in % of preferred drug items quarter immediately following PDI guidelines, (SE), 95%CI, p-value	1.21 (0.18), (0.84,1.57), p<0.001	1.21 (0.18), (0.83,1.59), p<0.001	1.22 (0.17), (0.85,1.59), p<0.001	1.26 (0.16), (0.90,1.61), p<0.001
Increase in % of preferred drug items per quarter post PDI guidelines, (SE), 95%CI, p-value	0.04 (0.04), (-0.03,0.12), p=0.25	0.06 (0.04), (-0.02,0.14), p=0.14	0.09 (0.04) (0.02,0.18), p=0.01	0.14 (0.03) (0.08,0.22), p<0.001
**simvastatin**				
Percentage of preferred drug items: beginning of study period (SE), 95% CI	5.94 (0.21), (5.50,6.38)	5.92 (0.17), (5.56,6.27)	5.89 (0.12), (5.63,6.15)	5.87 (0.07), (5.73,6.01)
Increase in % of preferred drug items per quarter following commencement of study period (SE), 95%CI, p-value	0.02 (0.03), (-0.04,0.07), p=0.54	0.02 (0.03), (-0.03,0.07), p=0.49	0.02 (0.02), (-0.03,0.07), p=0.32	0.02 (0.02), (-0.02,0.04), p=0.27
Increase in % of preferred drug items quarter immediately following PDI guidelines, (SE), 95%CI, p-value	0.30 (0.10), (0.10,0.50), p=0.01	0.30 (0.10), (0.10,0.51), p=0.01	0.32 (0.10), (0.12,0.52), p=0.01	0.42 (0.09), (0.23,0.62), p<0.001
Increase in % of preferred drug items per quarter post PDI guidelines, (SE), 95%CI, p-value	0.07 (0.02), (0.03,0.10), p=0.002	0.08 (0.02), (0.04,0.12), p=0.001	0.09 (0.02), (0.06,0.13), p<0.001	0.10 (0.01), (0.07,0.13), p<0.001

For peer review

Table A3 (cont): sensitivity analyses for segmented regression models

	Calendar quarters retained for analysis (Study Period)					
	**All available data: 11 quarters before guidelines, 13 quarters after guidelines (Jan 11-Dec 16)**	**11 quarters before guidelines, 11 quarters after guidelines (Jan 11-Jun 16)**	**11 quarters before guidelines, 9 quarters after guidelines (Jan 11-Dec 15)**	**9 quarters before guidelines, 13 quarters after guidelines (Jul 11-Dec 16)**	**9 quarters before guidelines, 11 quarters after guidelines (Jul 11-Jun 16)**	**9 quarters before guidelines, 9 quarters after guidelines (Jul 11-Jan 15)**
**ramipril**						
Percentage of preferred drug items: beginning of study period (SE), 95% CI	49.14 (0.07), (48.99,49.28)	49.14 (0.08), (48.97,49.30)	49.14 (0.07), (48.98,49.28)	49.93 (0.07), (49.78,50.08)	49.92 (0.07), (49.76,50.10)	49.94 (0.07), (49.78,50.10)
Increase in % of preferred drug items per quarter following commencement of study period (SE), 95%CI, p-value	0.38 (0.01), (0.35,0.40), p<0.001	0.38 (0.02), (0.35,0.40), p<0.001	0.38 (0.02). (0.35,0.40), p<0.001	0.37 (0.01), (0.34,0.40), p<0.001	0.37 (0.01). (0.34,0.40), p<0.001	0.37 (0.01). (0.33,0.40), p<0.001
Increase in % of preferred drug items quarter immediately following PDI guidelines, (SE), 95%CI, p-value	0.16 (0.07), (0.01,0.31), p=0.04	0.16 (0.07), (0.01,0.32), p=0.04	0.16 (0.08), (-0.01,0.33), p=0.04	0.16 (0.07), (0.02,0.34), p=0.03	0.17 (0.08), (0.01,0.35), p=0.04	0.18 (0.09), (-0.01,0.35), p=0.06
Increase in % of preferred drug items per quarter post PDI guidelines, (SE), 95%CI, p-value	0.41 (0.01), (0.39,0.42), p<0.001	0.40 (0.01), (0.38,0.43), p<0.001	0.41 (0.01), (0.38,0.44), p<0.001	0.41 (0.01), (0.39,0.42), p<0.001	0.40 (0.01), (0.38,0.43), p<0.001	0.41 (0.01), (0.38,0.44), p<0.001
**candesartan**						
Percentage of preferred drug items: beginning of study period (SE), 95% CI	11.90 (0.08), (11.73,12.07)	11.90 (0.09), (11.71,12.09)	11.89 (0.08), (11.73,12.06)	11.60 (0.09), (11.42,11.80)	11.61 (0.11), (11.40,11.84)	11.60 (0.08), (11.41,11.78)
Increase in % of preferred drug items per quarter following commencement of study period (SE), 95%CI, p-value	-0.15 (0.01), (-0.17,-0.12), p<0.001	-0.15 (0.01), (-0.17,-0.12), p<0.001	-0.16 (0.01), (-0.17,-0.12), p<0.001	-0.15 (0.01), (-0.17,-0.12), p<0.001	-0.15 (0.01), (-0.18,-0.12), p<0.001	-0.15 (0.01), (-0.18,-0.12), p<0.001
Increase in % of preferred drug items quarter immediately following PDI guidelines, (SE), 95%CI, p-value	0.15 (0.06), (0.02,0.29), p=0.03	0.14 (0.06), (0.01,0.29), p=0.04	0.16 (0.06), (0.02,0.30), p=0.03	0.14 (0.07), (0.01,0.28), p=0.04	0.14 (0.07), (-0.01,0.28), p=0.06	0.15 (0.06), (0.01,0.29), p=0.05
Increase in % of preferred drug items per quarter post PDI guidelines, (SE), 95%CI, p-value	0.01 (0.01), (-0.01,0.03), p=0.46	0.01 (0.01), (-0.02,0.03), p=0.75	0.02 (0.01), (-0.01,0.05), p=0.25	0.01 (0.01), (-0.01,0.03), p=0.49	0.01 (0.01), (-0.03,0.03), p=0.82	0.01 (0.01), (-0.01,0.05), p=0.31

Table A3 (cont): sensitivity analyses for segmented regression models

	Calendar quarters retained for analysis (Study Period)					
	**All available data: 13 quarters before guidelines, 11 quarters after guidelines (Jan 11-Dec 16)**	**13 quarters before guidelines, 9 quarters after guidelines (Jan 11-Jun 16)**	**11 quarters before guidelines, 11 quarters after guidelines (Jul 11-Dec 16)**	**11 quarters before guidelines, 9 quarters after guidelines (Jul 11-Jun 16)**	**9 quarters before guidelines, 11 quarters after guidelines (Jan 12-Dec 16)**	**9 quarters before guidelines, 9 quarters after guidelines (Jan 12-Jun 16)**
**venlafaxine**						
Percentage of preferred drug items: beginning of study period (SE), 95% CI	73.61 (0.44), (72.69,74.53)	73.61 (0.46), (72.63,74.60)	72.56 (0.35), (71.81,73.31)	72.57(0.38), (71.75,73.40)	71.45 (0.22), (70.98,71.91)	71.46 (0.24), (70.94,71.98)
Increase in % of preferred drug items per quarter following commencement of study period (SE), 95%CI, p-value	-0.35 (0.05), (-0.46,-0.24), p<0.001	-0.35 (0.05), (-0.46,-0.24), p<0.001	-0.32 (0.05), (-0.43,-0.21), p<0.001	-0.32 (0.05), (-0.43,-0.20), p<0.001	-0.25 (0.04), (-0.34,-0.16), p<0.001	-0.25 (0.05), (-0.35,-0.14), p<0.001
Increase in % of preferred drug items quarter immediately following PDI guidelines, (SE), 95%CI, p-value	0.71 (0.27), (0.15,1.27), p=0.02	0.71 (0.28), (0.12,1.29), p=0.02	0.71 (0.28), (0.12,1.31), p=0.02	0.70 (0.29), (0.08,1.32), p=0.03	0.79 (0.29), (0.16,1.42), p=0.02	0.78 (0.31), (0.10,1.45), p=0.03
Increase in % of preferred drug items per quarter post PDI guidelines, (SE), 95%CI, p-value	0.26 (0.13), (-0.02,0.55), p=0.07	0.26 (0.14), (-0.04,0.55), p=0.08	0.27 (0.13), (-0.01,0.56), p=0.05	0.26 (0.14), (-0.02,0.57), p=0.08	0.26 (0.12), (0.01,0.52), p=0.04	0.26 (0.13), (-0.02,0.53), p=0.07
Increase in % of preferred drug April-June 2015 following introduction of generic duloxetine, (SE), 95%CI, p-value	-0.09 (0.30), (-0.73,0.54), p=0.76	-0.09 (0.31), (-0.76,0.57), p=0.77	-0.11 (0.32), (-0.79,0.57), p=0.74	-0.11 (0.33), (-0.82,0.60), p=0.75	-0.15 (0.35), (-0.89,0.60), p=0.68	-0.17 (0.37), (-0.97,0.64), p=0.66
Increase in % of preferred drug items per quarter post June 2015 , (SE), 95%CI, p-value	-0.08 (0.09), (-0.10,0.26), p=0.34	0.14 (0.12), (-0.10,0.39), p=0.24	-0.08 (0.08), (-0.09,0.26), p=0.34	0.14 (0.11), (-0.10,0.39), p=0.26	0.07 (0.06), (-0.06,0.20), p=0.27	0.10 (0.09), (-0.10,0.33), p=0.27
**citalopram**						
Percentage of preferred drug items: beginning of study period (SE), 95% CI	23.58 (0.13), (23.31,23.85)	23.58 (0.12), (23.32,23.83)	22.88 (0.14), (22.89,23.17)	22.87 (0.13), (22.59,23.14)	21.95 (0.08), (21.78,22.12)	21.93 (0.06), (21.81,22.04)
Increase in % of preferred drug items per quarter following commencement of study period (SE), 95%CI, p-value	-0.36 (0.01), (-0.39,-0.33), p<0.001	-0.36 (0.01), (-0.39,-0.33), p<0.001	-0.36 (0.02), (-0.40,-0.32), p<0.001	-0.36 (0.02), (-0.40,-0.33), p<0.001	-0.33 (0.01), (-0.36,-0.30), p<0.001	-0.33 (0.01), (-0.36,-0.31), p<0.001
Increase in % of preferred drug items quarter immediately following PDI guidelines, (SE), 95%CI, p-value	0.30 (0.08), (0.12,0.47), p=0.002	0.30 (0.08), (0.12,0.48), p=0.003	0.30 (0.09), (0.11,0.48), p=0.003	0.30 (0.09), (0.11,0.50), p=0.005	0.30 (0.08), (0.13,0.47), p=0.002	0.34 (0.08), (0.17,0.51), p=0.001
Increase in % of preferred drug items per quarter post PDI guidelines, (SE), 95%CI, p-value	-0.23 (0.02), (-0.27,-0.19), p<0.001	-0.22 (0.02), (-0.26,-0.18), p<0.001	-0.23 (0.02), (-0.27,-0.19), p<0.001	-0.22 (0.02), (-0.27,-0.17), p<0.001	-0.24 (0.02), (-0.26,-0.21), p<0.001	-0.23 (0.01), (-0.25,-0.20), p<0.001

For peer review only - http://bmjopen.bmj.com/site/about/guidelines.xhtml

Table A3 (cont): sensitivity analyses for segmented regression models

	Calendar quarters retained for analysis (Study Period)			
	**All available data: 15 quarters before guidelines, 9 quarters after guidelines (Jan 11-Dec 16)**	**13 quarters before guidelines, 9 quarters after guidelines (Jul 11-Dec 16)**	**11 quarters before guidelines, 9 quarters after guidelines (Jan 12-Dec 16)**	**9 quarters before guidelines, 9 quarters after guidelines (Jul 12-Dec 16)**
**ER tolterodine**				
Percentage of preferred drug items: beginning of study period (SE), 95% CI	37.27 (0.27), (36.69,37.84)	35.45 (0.30), (34.81,36.09)	33.16 (0.33), (32.46,33.87)	31.10 (0.39) (30.29,31.93)
Increase in % of preferred drug items per quarter following commencement of study period (SE), 95%CI, p-value	-1.00 (0.05), (-1.11,-0.88), p<0.001	-1.04 (0.07), (-1.21,-0.88), p<0.001	-0.97 (0.11), (-1.21,-0.73), p<0.001	-0.98 (0.06), (-1.11,-0.86), p<0.001
Increase in % of preferred drug items Jan-Mar 2013 following licensing of mirabegron, (SE), 95%CI, p-value	0.16 (0.24), (-0.35,0.66), p=0.52	0.21 (0.26), (-0.34,0.75), p=0.43	0.11 (0.25), (-0.44,0.65), p=0.68	*
Increase in % of preferred drug items per quarter post March 2013 (SE), 95%CI, p-value	-1.04 (0.06), (-1.17,-0.91), p<0.001	-1.03 (0.07), (-1.17,-0.89), p<0.001	-1.03 (0.06), (-1.17,-0.89), p<0.001	*
Increase in % of preferred drug items quarter immediately following PDI guidelines, (SE), 95%CI, p-value	-0.06 (0.24), (-0.57,0.45), p=0.82	-0.05 (0.25), (-0.57,0.49), p=0.86	-0.01 (0.24), (-0.51,0.49), p=0.96	-0.05 (0.22), (-0.52,0.43), p=0.86
Increase in % of preferred drug items per quarter post PDI guidelines, (SE), 95%CI, p-value	-0.63 (0.09), (-0.73,-0.52), p<0.001	-0.63 (0.06), (-0.73,-0.51), p<0.001	-0.62 (0.05), (-0.73,-0.51), p<0.001	-0.63 (0.06), (-0.75,-0.50), p<0.001

*omitted due to close proximity of study period (July 2012) and licensing of mirabegron (Jan 2013)

For peer review only

Table A4: Sensitivity analyses: alternative definition of calendar quarters for ACE inhibitors/ARBs

	**Calendar quarters: Jan-Mar, Apr-Jun, Jul-Sep, Oct-Dec (24 calendar quarters: Jan 11-Dec 16)**	**Calendar quarters: Mar-May, Jun-Aug, Sep-Nov, Dec-Feb (23 calendar quarters: Mar 11-Nov 16)**
**ramipril**		
Percentage of preferred drug items: beginning of study period (SE), 95% CI	49.14 (0.07), (48.99,49.28)	49.25 (0.07), (49.27, 49.58)
Increase in % of preferred drug items per quarter following commencement of study period (SE), 95%CI, p-value	0.38 (0.01), (0.35,0.40), p<0.001	0.37 (0.01), (0.34,0.40), p<0.001
Increase in % of preferred drug items quarter immediately following PDI guidelines, (SE), 95%CI, p-value	0.16 (0.07), (0.01,0.31), p=0.04	0.14 (0.08), (-0.01,0.30), p=0.05
Increase in % of preferred drug items per quarter post PDI guidelines, (SE), 95%CI, p-value	0.41 (0.01), (0.39,0.42), p<0.001	0.41 (0.01), (0.39,0.43), p<0.001
**candesartan**		
Percentage of preferred drug items: beginning of study period (SE), 95% CI	11.90 (0.08), (11.73,12.07)	11.78 (0.07), (11.63,11.92)
Increase in % of preferred drug items per quarter following commencement of study period (SE), 95%CI, p-value	-0.15 (0.01), (-0.17,-0.12), p<0.001	-0.15 (0.01), (-0.17,-0.13), p<0.001
Increase in % of preferred drug items quarter immediately following PDI guidelines, (SE), 95%CI, p-value	0.15 (0.06), (0.02,0.29), p=0.03	0.17 (0.06), (0.06,0.29), p=0.01
Increase in % of preferred drug items per quarter post PDI guidelines, (SE), 95%CI, p-value	0.01 (0.01), (-0.01,0.03), p=0.46	0.01 (0.01), (-0.02,0.02), p=0.90

For peer review only

STROBE 2007 (v4) checklist of items to be included in reports of observational studies in epidemiology*
Checklist for cohort, case-control, and cross-sectional studies (combined)

Section/Topic	Item #	Recommendation	Reported on page #
**Title and abstract**	1	(*a*) Indicate the study's design with a commonly used term in the title or the abstract	5
		(*b*) Provide in the abstract an informative and balanced summary of what was done and what was found	2
**Introduction**			
Background/rationale	2	Explain the scientific background and rationale for the investigation being reported	4
Objectives	3	State specific objectives, including any pre-specified hypotheses	4
**Methods**			
Study design	4	Present key elements of study design early in the paper	5
Setting	5	Describe the setting, locations, and relevant dates, including periods of recruitment, exposure, follow-up, and data collection	5
Participants	6	(*a*) *Cohort study*—Give the eligibility criteria, and the sources and methods of selection of participants. Describe methods of follow-up<br>*Case-control study*—Give the eligibility criteria, and the sources and methods of case ascertainment and control selection. Give the rationale for the choice of cases and controls<br>*Cross-sectional study*—Give the eligibility criteria, and the sources and methods of selection of participants	5
		(*b*) *Cohort study*—For matched studies, give matching criteria and number of exposed and unexposed<br>*Case-control study*—For matched studies, give matching criteria and the number of controls per case	
Variables	7	Clearly define all outcomes, exposures, predictors, potential confounders, and effect modifiers. Give diagnostic criteria, if applicable	5
Data sources/ measurement	8*	For each variable of interest, give sources of data and details of methods of assessment (measurement). Describe comparability of assessment methods if there is more than one group	*5*
Bias	9	Describe any efforts to address potential sources of bias	10-all available data used
Study size	10	Explain how the study size was arrived at	5-all available data used
Quantitative variables	11	Explain how quantitative variables were handled in the analyses. If applicable, describe which groupings were chosen and why	5-groupings as per medicine group
Statistical methods	12	(*a*) Describe all statistical methods, including those used to control for confounding	5/6
		(*b*) Describe any methods used to examine subgroups and interactions	n/a

For peer review only

		(*c*) Explain how missing data were addressed	n/a
		(*d*) *Cohort study*—If applicable, explain how loss to follow-up was addressed<br>*Case-control study*—If applicable, explain how matching of cases and controls was addressed<br>*Cross-sectional study*—If applicable, describe analytical methods taking account of sampling strategy	n/a
		(*e*) Describe any sensitivity analyses	6, Appendix
**Results**			
Participants	13*	(a) Report numbers of individuals at each stage of study—eg numbers potentially eligible, examined for eligibility, confirmed eligible, included in the study, completing follow-up, and analysed	7
		(b) Give reasons for non-participation at each stage	n/a
		(c) Consider use of a flow diagram	N/A
Descriptive data	14*	(a) Give characteristics of study participants (eg demographic, clinical, social) and information on exposures and potential confounders	N/A
		(b) Indicate number of participants with missing data for each variable of interest	N/A
		(c) *Cohort study*—Summarise follow-up time (eg, average and total amount)	All data 2011-2016 used
Outcome data	15*	*Cohort study*—Report numbers of outcome events or summary measures over time	N/a
		Case-control study—Report numbers in each exposure category, or summary measures of exposure	N/A
		Cross-sectional study—Report numbers of outcome events or summary measures	7, Table 1
Main results	16	(*a*) Give unadjusted estimates and, if applicable, confounder-adjusted estimates and their precision (eg, 95% confidence interval). Make clear which confounders were adjusted for and why they were included	7,8
		(*b*) Report category boundaries when continuous variables were categorized	n/a
		(*c*) If relevant, consider translating estimates of relative risk into absolute risk for a meaningful time period	8
Other analyses	17	Report other analyses done—eg analyses of subgroups and interactions, and sensitivity analyses	8, Appendix
**Discussion**			
Key results	18	Summarise key results with reference to study objectives	9
Limitations	19	Discuss limitations of the study, taking into account sources of potential bias or imprecision. Discuss both direction and magnitude of any potential bias	10
Interpretation	20	Give a cautious overall interpretation of results considering objectives, limitations, multiplicity of analyses, results from similar studies, and other relevant evidence	9/10
Generalisability	21	Discuss the generalisability (external validity) of the study results	9/10
**Other information**			
Funding	22	Give the source of funding and the role of the funders for the present study and, if applicable, for the original study on which the present article is based	12

For peer review only

*Give information separately for cases and controls in case-control studies and, if applicable, for exposed and unexposed groups in cohort and cross-sectional studies.

Note: An Explanation and Elaboration article discusses each checklist item and gives methodological background and published examples of transparent reporting. The STROBE checklist is best used in conjunction with this article (freely available on the Web sites of PLoS Medicine at http://www.plosmedicine.org/, Annals of Internal Medicine at http://www.annals.org/, and Epidemiology at http://www.epidem.com/). Information on the STROBE Initiative is available at www.strobe-statement.org.

For peer review only

An evaluation of prescribing trends and patterns of claims within the Preferred Drugs Initiative in Ireland (2011-2016): an interrupted time-series study

Journal:	*BMJ Open*
Manuscript ID	bmjopen-2017-019315.R2
Article Type:	Research
Date Submitted by the Author:	17-Nov-2017
Complete List of Authors:	McDowell, Ronald; Royal College of Surgeons in Ireland, HRB Centre for Primary Care Research<br>Bennett, Kathleen; Royal College of Surgeons in Ireland, Population Health Sciences<br>Moriarty, Frank; Royal College of Surgeons in Ireland, HRB Centre for Primary Care Research, Department of General Practice<br>Clarke, Sarah; Health Service Executive Medicines Management Programme<br>Barry, Michael; National Centre for Pharmacoeconomics, Trinity Centre for Health Sciences<br>Fahey, Tom; Royal College of Surgeons in Ireland, Department of General Practice
**Primary Subject Heading**:	Epidemiology
Secondary Subject Heading:	General practice / Family practice, Health policy, Public health
Keywords:	Ireland, physician prescribing patterns, interrupted time series

SCHOLARONE™
Manuscripts

An evaluation of prescribing trends and patterns of claims within the Preferred Drugs Initiative in Ireland (2011-2016): an interrupted time-series study

McDowell RD[1], Bennett K[2], Moriarty F[1], Clarke S[3], Barry M[4], Fahey T[1]

[1] HRB Centre for Primary Care Research,
Department of General Practice,
Royal College of Surgeons in Ireland Medical School,
123 St Stephens Green,
Dublin 2

[2] Division of Population Health Sciences,
Royal College of Surgeons in Ireland,
Beaux Lane House,
Mercer Street Lower,
Dublin 2

[3] Health Service Executive Medicines Management Programme,
Trinity Centre for Health Sciences,
St. James's Hospital,
Dublin 8

[4] National Centre for Pharmacoeconomics,
Trinity Centre for Health Sciences,
St. James's Hospital,
Dublin 8

Correspondence to:

Ronald McDowell
Email: ronaldmcdowell@rcsi.ie

Word count: 4,131

Keywords: Ireland, physician prescribing patterns, interrupted time series

For peer review only

For peer review only - http://bmjopen.bmj.com/site/about/guidelines.xhtml

Abstract

Objective: To examine the impact of the Preferred Drugs Initiative (PDI), an Irish health policy aimed at enhancing evidence-based cost-effective prescribing, on prescribing trends and the cost of prescription medicines across seven medication classes.

Design: Retrospective repeated cross-sectional study spanning the years 2011 to 2016.

Setting: Health Service Executive Primary Care Reimbursement Scheme pharmacy claims data for General Medical Services (GMS) patients, approximately 40% of the Irish population.

Participants: Adults aged ≥18 years between 2011 to 2016 eligible for the GMS scheme.

Primary and secondary outcomes: The percentage of PDI medications within each drug class per calendar quarter. Linear regression was used to model prescribing of the preferred drug within each medication group and to assess the impact of PDI guidelines and other relevant changes in prescribing practice. Savings in drug expenditure were estimated.

Results: Between 2011 and 2016 around one quarter (23.59%) of all medications were for single-agent drugs licensed in the seven drug classes. There was a small increase in the percentage of PDI drugs, increasing from 4.64% of all medications in 2011 to 4.76% in 2016 (p<0.001). The percentage of preferred drugs within each drug class was significantly higher immediately following publication of the guidelines for all classes except urology, with the largest increases noted for lansoprazole (1.21%, 95%CI: 0.84% to 1.57%, p<0.001) and venlafaxine (0.71%, 95%CI: 0.15% to 1.27%), p=0.02). Trends in prescribing of the preferred drugs between PDI guidelines and the end of 2016 varied between drug classes. Total cost savings between 2013 and 2016 were estimated to be €2.7million.

Conclusion: There has been a small increase in prescribing of PDI drugs in response to prescribing guidelines, with inconsistent changes observed across therapeutic classes. These findings are relevant where health services are seeking to develop more active prescribing interventions aimed at changing prescribing practice.

For peer review only - http://bmjopen.bmj.com/site/about/guidelines.xhtml

Strengths and limitations of this study

- PCRS data covers pharmacy claims for prescriptions issued to General Medical Services (GMS) Scheme eligible patients (around 40% of the Irish population)
- Methods used are appropriate given the phased introduction of the preferred drug guidelines
- GMS patients over-represent older adults and those in receipt of social welfare
- Results based on aggregated data give an overview of the Preferred Drugs Initiative in its early years but require further detailed analysis to examine prescriber and patient heterogeneity.

For peer review only

Background

The Health Service Executive (HSE) in Ireland spent €1.05 billion in 2015 reimbursing pharmacists for the cost of prescription items issued to General Medical Services (GMS) eligible patients via the Primary Care Reimbursement Scheme (PCRS).(1) This is the largest community drug scheme in Ireland, providing access to free or minimal cost health care for patients whose household income falls below the eligibility threshold specified by the Irish Government, as well as the majority of people aged ≥70 years (approximately 95%) where a higher income threshold applies. Currently GMS eligible patients in Ireland have their prescription charges paid directly by the State, with a patient-levy of €2.50 for each item dispensed, up to a maximum of €25 per month. Historically Ireland has spent as much as 50% above the EU average per capita on drugs for a variety of reasons, such as low levels of use of generic medications and higher negotiated prices with pharmaceutical companies for both patented and generic drugs.(2, 3)

Against the background of an ageing population (4), the economic downturn of 2008 and rising drug costs the HSE established the Medicines Management Programme (MMP) in 2013. The MMP has undertaken a number of initiatives aimed at enhancing evidence-based and cost-effective prescribing (5), one of which is the Preferred Drugs Initiative (PDI). The PDI recommends a single 'preferred drug' within a therapeutic drug class as the prescriber's drug of first choice. Factors considered when selecting the preferred drug include clinical efficacy, ease of administration, the possibility of side effects or interactions with other drugs, cost, and national and international clinical guidelines. Recommendations for preferred drugs are made on an ongoing basis, with the findings disseminated through the publication of prescribing guidelines and GP meetings. The regulations covering generic substitution of branded medications are separate to the PDI guidelines, with generic substitution of drugs implemented where possible unless there are clinical reasons for prescribing the branded medication. The issuing of preferred drugs is voluntary and no incentives are given to prescribers to issue the preferred drug instead of others from within the same therapeutic drug class, with the patient levy remaining unaltered irrespective of preferred- or non-preferred drug status. Although the preferred drug may not necessarily be the least expensive licensed medication within each drug class, it has been estimated that increased provision of the preferred drugs could save the HSE €15 million per year.(5)

For peer review only - http://bmjopen.bmj.com/site/about/guidelines.xhtml

As of September 2016 reports detailing the rationale behind the choice of the preferred drugs have been published for the first ten therapeutic drug classes covered by the Initiative.(6) These are proton pump inhibitors (PPIs), statins, angiotensin-converting enzyme (ACE) inhibitors, angiotensin-II receptor blockers (ARBs), serotonin noradrenaline reuptake inhibitors (SNRIs), selective serotonin reuptake inhibitors (SSRIs), medications for treating urological conditions (urinary incontinence, frequency and overactive bladder), oral anticoagulants for stroke prevention in patients with non-valvular atrial fibrillation, beta-blockers and calcium channel blockers. There has been no evaluation of changes in prescribing following the introduction of the PDI to date. The aims of this paper are to: (i) examine the trends and patterns of pharmacy claims for seven PDI drug classes among eligible adult GMS patients in Ireland between 2011 and 2016; (ii) assess the impact of the PDI recommendations over time using segmented regression analysis; and (iii) estimate the cost savings due to the PDI during these years.

For peer review only

For peer review only - http://bmjopen.bmj.com/site/about/guidelines.xhtml

Methods

The STrengthening the Reporting of Observational Studies in Epidemiology (STROBE) guidelines were used in the reporting of this study (7).

Data

HSE-PCRS monthly pharmacy claims were analysed from 2011 to 2016 (8). This study period provided an average of three years of claims data both before and after the PDI across the seven drug classes considered. The data includes all pharmacy claims made for GMS patients and for which the cost of the claim has been reimbursed to community pharmacies by the HSE.

Preferred Drugs Initiative

The first seven medication classes covered by the PDI are considered in this paper. The preferred drugs in each of these classes were lansoprazole (PPIs), simvastatin (statins), ramipril (ACE inhibitors), candesartan (ARBs), venlafaxine (SNRIs), citalopram (SSRIs) and extended release (ER) tolterodine (urology medications). Guidelines for beta-blockers and calcium channel blockers were introduced in September 2016. Prescriptions issued to children (those under 18 years), hospital emergency items, out-of-hours prescriptions and items not considered medications (such as medical devices and dressings) were excluded; the PDI is primarily aimed at the treatment of adults in the general population.

Analytical methods/approach

Descriptive statistics were used to summarise relevant medications from the HSE-PCRS database and the classes of PDI drugs. Only single-agent drugs are considered in this paper, as this is the primary focus of the PDI.

The time-scale used for the analyses of time series depends on the research question of interest (9). Calendar quarters (January-March, April-June, July-September, October-December) were used to aggregate the data consistent with other analyses of prescribing data using interrupted time series (10-12). The use of calendar quarters was deemed clinically appropriate: changes in prescribing patterns tend to be gradual and guidelines are not necessarily disseminated or actioned on the first day of each calendar month. Furthermore Irish GMS eligible patients in receipt of prescription medication can receive three-months' worth of repeat prescriptions per consultation with their GP. For each therapeutic drug class a linear

For peer review only - http://bmjopen.bmj.com/site/about/guidelines.xhtml

regression model was used to estimate the percentage of the preferred drug per drug class per calendar quarter between 2011 to 2016, allowing for any changes that might have taken place following issuing of guidelines or other changes in clinical practice. This is a commonly-used strategy for analysing interrupted time series.(13) For medicine groups where the only “interruption” considered was dissemination of PDI guidelines, the regression equations used had the form

$$p_{ij} \quad = \left(\beta_{oj} + \beta_{1j}x_{ij1} + \beta_{2j}x_{ij2} + \beta_{3j}x_{ij3}\right) + e_{ij} \qquad (i = 0, \ldots, 23)$$

where for each medicine group j $(j = 1, \ldots, 7)$

p_{ij} is the percentage of items of the preferred drug reimbursed at time (quarter) i

β_{oj} is the estimated percentage of items being preferred drugs at t=0 (Jan-Mar 2011),

β_{1j} is the estimated change in the percentage of items being preferred drugs immediately following guidelines (the “change of level”)

β_{2j} is the estimated change in the percentage of items being preferred drugs per calendar quarter (the “slope”) before the guidelines

β_{3j} is the estimated change in the percentage of items being preferred drugs per calendar quarter (the “slope”) post -guidelines

e_{ij} is the residual for calendar quarter i.

The x_{ijk} $(k = 1,2,3)$ were calculated from the data according to standard practice. (14)

More than one change of level can be incorporated into any interrupted time series where this is relevant to the research question (13, 15). It was not feasible to include changes in the price of drugs in these models given the large number of drugs considered. Across the drug classes all drugs were licensed and available in Ireland between 2011 and 2016, and all generics were licensed prior to the study period, the key exceptions being the licensing of generic duloxetine in April 2015 and the licensing of mirabegron in January 2013. These two events were incorporated into the analyses for SNRIs and urology medications respectively.

Examination of the autocorrelation and partial autocorrelation coefficients showed that there was significant residual autocorrelation between adjacent calendar quarters (but not between non-adjacent quarters) in each drug group, and this was incorporated into the models using Prais–Winsten regression (16). The potential for seasonal autocorrelation was also considered:

For peer review only - http://bmjopen.bmj.com/site/about/guidelines.xhtml

in this context seasonal autocorrelation would mean that a given medication within a drug class is on average more or less likely to be prescribed than other drugs in the same class by virtue of the time of year. The PDI guidelines do not refer to any such clinical considerations (6) and we additionally hypothesised that seasonal autocorrelation would not be of statistical significance. This hypothesis was tested for each drug class by comparing the regression models which included Fourier terms to account for seasonality (9) and models without the seasonality terms. For each drug class seasonal autocorrelation was not of statistical significance and the seasonality terms were removed on the grounds of parsimony.

The PDI guidelines were national guidelines and consequently no control groups were available with which to compare prescribing under the PDI. However, we constructed two reference groups using the drug classes beta-blockers and calcium channel blockers. These were drug classes for which PDI guidelines were launched in September 2016 (the preferred drugs being bisoprolol and amlodipine respectively) but for which no recommendations had been made when the PDI guidelines were launched for the other drug classes. Given that the earlier guidelines were launched within six months of each other, two additional models were fitted: one examining prescribing of bisoprolol as the preferred beta-blocker over the study period, allowing for potential changes in prescribing when guidelines for PPIs/statins (April 2013) and SNRIs/SSRIs (April 2014) were disseminated, and one model examining prescribing of amlodipine as the preferred calcium channel blocker, allowing for potential changes in prescribing when guidelines for ACE inhibitors/ARBs (Sept 2013) and urology medications (October 2014) were issued.

By coincidence rather than design issuing of guidelines for each medicine group occurred at the beginning of the calendar quarters listed above, with the exception of the guidelines for ACE inhibitors and ARBs. Sensitivity analyses were used to explore whether the results varied when the calendar quarters were constructed differently (March-May, June-August, September-November, December-February) for these groups. Given that the PDI guidelines were launched in phases, sensitivity analyses were also used to examine whether results were dependent on the length of time considered before and after guidelines.

The models above were used to estimate increases or decreases in costs for each drug group associated with the PDI. Where only one interruption to the time series was included in the model, the predicted number of preferred drug items from each class was compared with the

For peer review only - http://bmjopen.bmj.com/site/about/guidelines.xhtml

number which would have been issued had the trend in prescribing estimated before the guidelines continued i.e. the estimates of $\beta_{oj}(\widehat{\beta_{oJ}})$ and $\beta_{2j}\,(\widehat{\beta_{2J}})$ remained unchanged, $\widehat{\beta_{1J}}$ was constrained to be zero and the estimate of $\beta_{3j}(\widehat{\beta_{3J}})$ was set equal to $\widehat{\beta_{2J}}$. The difference in the number of preferred drug items under the two scenarios was multiplied by the average price of the preferred drug, calculated across all reimbursements between dissemination of the guidelines and the end of 2016. The difference in the number of non-preferred drug items was multiplied by a weighted average of the price of all other drugs from within the medicine class, weighted according to the overall distribution of these items between issuing of the guidelines and the end of December 2016. These two costs were combined to give an overall cost differential. The process was extended analogously to include multiple interruptions as appropriate.

All analyses were conducted using Stata 14.0SE.(17) Results were held to be significant if they referred to statistical significance on a two-sided design-based test evaluated at the 0.05% level.

For peer review only

For peer review only - http://bmjopen.bmj.com/site/about/guidelines.xhtml

Results

Descriptive statistics

A total of 336,535,263 prescription items for medications were reimbursed by 4,465 PCRS prescribers for 1,919,681 GMS adults aged 18 years and over between 2011 and 2016. The median number of items reimbursed per GMS patient was 63 (Interquartile Range (IQR) 13 to 246) with a median total cost per patient of €905.75 (IQR €170.25 to €9,726.93). Approximately 55 million items were reimbursed per year, with the number of items peaking slightly in 2012 and 2013. During the six-year period 48.8 million (19.86%) prescription items were for the single-agent medicines licensed across the seven therapeutic drug classes considered. The drug classes most commonly prescribed to GMS patients were statins (5.93% of all items) and PPIs (5.63%), with the least common being SNRIs (0.99%) and drugs for treating urological conditions (0.67%). The descriptive statistics for each PDI medication class over the six-year period are outlined in Table 1.

The percentage of items relating to the seven drug classes increased slightly from 19.57% in 2011 to 20.04% in 2016, with small changes observed in the volume of prescriptions issued per each PDI medicine group over this time. More detailed breakdowns of PDI medicine groups per calendar year and quarter are given in Appendix Tables A1 & A2 and Figure A1.

Preferred Drugs Initiative

Within the seven PDI drug classes considered, 23.59% of all prescription items were for the named preferred drugs. However, there was considerable variation between PDI drug classes both in terms of ranking and percentage coverage of the preferred drug (see Table 1). The most commonly prescribed preferred drug within the relevant drug class was venlafaxine, which comprised 70.99% of all SNRI prescriptions. This was followed by ramipril (53.41% of all single-agent ACE inhibitors), ER tolterodine (25.79% of urology items), lansoprazole (24.14% of PPIs), citalopram (19.77% of SSRIs), candesartan (10.78% of all single-agent ARBs) and simvastatin (6.59% of all single-agent statins). The ranking of the preferred drugs within classes varied from first (ACE inhibitors and SNRIs), to second-last (statins). There was a small but statistically significant increase over time in the percentage of all medications which were for the PDI drugs, increasing from 4.64% in 2011 to 4.76% in 2016 ($p<0.001$).

Impact of clinical guidelines

For peer review only - http://bmjopen.bmj.com/site/about/guidelines.xhtml

Comparing prescribing patterns within each medication class in the three months pre-and post-publication of the PDI guidelines there was a small increase in the proportion of preferred drugs in four drug classes (PPIs (p<0.001), statins (p<0.001), ACE inhibitors (p<0.001) and SNRIs (p=0.08)), little change in two other drugs classes (ARBs (p=0.76) and SSRIs (p=0.37)), and a reduction in percentage terms in prescribing of the PDI agent ER tolterodine (p<0.001) (Table 1). Two preferred drugs, citalopram and ER tolterodine, were ranked lower within their respective classes between issuing of the guidelines and the end of 2016 than before. Figure 1 illustrates the secular trends for preferred drugs across the PDI categories by calendar quarters between 2011 and 2016: plots of the actual percentage of preferred drug items within each drug group between 2011 and 2016 are given in Appendix Figure A2.

Segmented linear regression showed changes over time in the prescribing of all preferred drugs (Table 2). In all medicine groups except urology, there was evidence of significant increases in prescribing of the preferred drugs immediately following dissemination of the PDI guidelines . For three medicine groups, there was significant evidence of an increase in the percentage of preferred drug items in the quarter immediately following issuing of the guidelines (lansoprazole (1.21%, 95%CI: 0.84% to 1.57%, p<0.001); venlafaxine (0.71%, 95%CI: 0.15% to 1.27%, p<0.001); simvastatin (0.30%, 95%CI: 0.1% to 0.5%, p=0.01)) and small increases in prescribing of the preferred drug in subsequent quarters. The percentage of SNRI medications which were venlafaxine did not change significantly immediately following the licensing of generic duloxetine in April 2015 (p=0.76) or in subsequent quarters (p=0.34). For both candesartan and citalopram, for which prescribing within their PDI drug classes was in decline prior to the guidelines being issued, prescribing increased immediately following the PDI guidelines (candesartan (0.15%, 95%CI: 0.02 to 0.29, p=0.03); citalopram (OR 0.30%, 95%CI: 0.12% to 0.47%, p=0.002)) but did not continue to increase significantly in subsequent quarters. Indeed declines in prescribing of citalopram resumed in July 2014, although the decline was less steep than before the guidelines (p<0.001). There was a small increase in prescribing of the preferred ACE inhibitor (ramipril) immediately following the PDI guidelines (0.16%, 9%CI: 0.01 to 0.31, p=0.04), although subsequent increases per calendar quarter did not differ significantly at the 5% level from increases observed per calendar quarter prior to the PDI guidelines (p=0.08). No statistically significant changes were observed in the prescribing of ER tolterodine immediately following the licensing of mirabegron in January 2013 (p=0.52) or the PDI guidelines in October 2014 (p=0.82), although the rate of decline in prescribing of ER

For peer review only - http://bmjopen.bmj.com/site/about/guidelines.xhtml

tolterodine was lower following the PDI guidelines than between the licensing of mirabegron and dissemination of the PDI guidelines ($p<0.001$).

Sensitivity analyses showed that the results were materially unaffected when the calendar quarters used for analyses of ACE inhibitors and ARBs varied or when the length of time studied before and after the guidelines was changed (Appendix Tables A3, A4).

Reference groups

Beta-blockers and calcium channel blockers accounted for 3.58% (n=12,056,378) and 2.30% (n=7,753,755) of single-agent medications for GMS patients between 2011 and 2016, with the most commonly prescribed medications being bisoprolol (56.83% of all single-agent beta-blockers (n=6,852,022)) and amlodipine (64.70% of all single-agent calcium channel blockers (n=5,016,348)), both of which were selected as preferred drugs in September 2016. There was a steady increase in prescribing of bisoprolol as the beta-blocker of choice and a consistent fall in prescribing of amlodipine within the calcium channel blocker medications over the study period. Effects in these drug groups associated with dissemination of the PDI guidelines for the other drug groups were non-significant at the 5% level (Table 3). See Figure 2 for plots of the estimated percentage of preferred drug items within each therapeutic drug class between 2011 and 2016.

Cost savings

Overall, the cost savings after introduction of the PDI amounted to €2,671k across all seven PDI drug classes (Table 2). The savings associated with changes in prescribing following issuing of guidelines for the seven drug classes were estimated to be €123k in 2013, €396k in 2014, €837k in 2015 and €1,314k in 2016. There were savings in each group, even though changes in dispensed medications were often minimal. The greatest impact was on the amount spent on SNRIs, with an estimated saving of €1,291k between 2014-2016. This is due to the much higher cost of the non-preferred drug duloxetine to the preferred drug venlafaxine. Other groups where the savings were marked were for the two larger volume groups where the guidelines had first been issued- PPIs saving €618k and statins saving €363k. For medicine groups where prescribing of the preferred drug was in decline before guidelines were issued, even the small short-term changes in prescribing translated into some savings. The smallest cost savings were

For peer review only - http://bmjopen.bmj.com/site/about/guidelines.xhtml

in the prescribing of ramipril and ER tolterodine, due to the lack of change in prescribing trends observed within these groups between 2011 and 2016. The combined savings in the reference groups, had the prescribing patterns observed prior to the PDI guidelines remained unchanged, was an estimated €17k.

For peer review only

For peer review only - http://bmjopen.bmj.com/site/about/guidelines.xhtml

Discussion

Principal findings

The seven drug classes considered that form part of the PDI accounted for approximately 20% of all medications reimbursed by the PCRS between 2011 and 2016. Changes in prescribing observed over the study period varied by PDI drug class, with substantial differences in the ranking order and quantity of preferred drug prescribed. Overall, the impact of the PDI guidance was limited, with an inconsistent pattern observed across all therapeutic drug classes, and only a small increase (0.13%) in the percentage of preferred drugs issued overall between 2011 and 2016. Across the PDI drug classes some differences emerged: in the first group of PDI drugs there were increases in prescribing of the preferred drug immediately following issuing of the guidelines and continued though small increases subsequently (PPIs, Statins and SNRIs); in the second group of PDI drugs (SSRIs and ARBs) there was a temporary increase in prescribing of the preferred drug just after the guidelines were issued; lastly, in the third group of PDI drugs (ACE and urology), there appeared to be little or no impact of clinical guidance. The reasons for such diversity are not known. ACE inhibitors are relatively inexpensive and this may account, in part, for the trend in ramipril prescribing remaining relatively unchanged. Although mirabegron has become the most commonly prescribed urology item since its launch in 2013, prescribing of ER Tolterodine was in decline prior to this time.

Context of other studies

PDI guidelines to date have been disseminated to prescribers mainly through correspondence and GP meetings. The literature shows that educational programmes and publication of guidelines in themselves tend to have little effect on influencing prescribing practice, and that these need to be enhanced with other strategies.(18) In a systematic review of 79 studies examining interventions which changed doctor prescribing behaviour, the most effective interventions were patient-mediated interventions, outreach, audit and feedback, and reminders.(19) In a study of changes in the use of losartan versus other single ARBs in Sweden investigators concluded that multiple and intensive demand-side measures are needed to change physician prescribing habits.(20) Other strategies which have been found to be helpful include direct involvement of the community pharmacist and face-to-face engagement from those seeking to encourage change with the prescriber .(21) Technological advances, such as alerts and prompts when issuing a drug may also prove useful. (22)

For peer review only - http://bmjopen.bmj.com/site/about/guidelines.xhtml

Any excess expenditure incurred through the issuing of non-preferred drugs to GMS patients is met directly by the HSE and not by the patient. Options which could reduce such expenditure include reducing choice for either patient or prescriber. It has been suggested that because prescribers can develop expertise of only a certain number of drugs, more restrictive formularies may also provide benefits to quality of prescribing (23, 24). In Sweden, the introduction of the 'Wise List', an evidence-based formulary of essential medicines, increased adherence to guideline recommendations in primary care from 80% to 90% and reduced variation in prescribing (25). The introduction of co-payments, where the patient has to pay the difference between the price of the preferred drug and their chosen alternative, has the potential to be a considerable driver of change. Australia operates a therapeutic brand premium scheme, whereby a co-payment is required from patients when a prescriber has issued a drug within a drug class that is priced above the benchmark for drugs in that group.(26) While dramatic changes in co-payments may result in more patients switching to preferred agents (such as statins, ACE inhibitors and PPIs), they may also increase the risk of patients stopping their medication or becoming non-adherent (27, 28). Recent work has shown the drivers of drug expenditure in high income countries varies substantially, with several other factors aside from physician prescribing behavior and patient preference determining national drug expenditure.(29)

<u>Strengths and limitations</u>

There are a number of strengths to this study. Our prescription sample is large and generalisable: PCRS data covers the entire GMS population of Ireland (around 40% of individuals). Despite the guidelines being introduced incrementally, the results were invariant to the time periods studied pre- and post-publication of clinical guidelines. However, there are limitations to the study. GMS patients are weighted towards older adults and those socially and financially disadvantaged and so the results may not be reflective of the entire population in receipt of prescription medication. There is no way of knowing whether prescribers approached patients with regards to changes in their medication and/or whether these approaches were successful. Patient-specific factors may mean that issuing of the preferred drug may not have been appropriate or possible. Neither prescribers nor patients are homogeneous entities and considerable variation may exist within both.

For peer review only - http://bmjopen.bmj.com/site/about/guidelines.xhtml

Policy implications and future research

The PDI has been developed to encourage evidence-based, cost-effective prescribing, but in view of the limited changes to date has delivered only a small amount of cost savings in terms of the money spent on these prescription items. If cost savings are to be maximised, the energies need to focus on medicine groups which are large volume (e.g. PPIs and statins) and/or where there is considerable variation between the least and most expensive licensed medications in that group (e.g. SNRIs). To enhance the impact of the PDI, multi-faceted interventions appear most likely to succeed. Financial incentives to prescribers may be one possible component of such interventions, as operated in Irish primary care for a time in the 1990's (30), however any incentives for PDI drugs need to be aligned with professional values of prescriber, and be mindful of personal preferences of patients taking long-term medication (31-33). The effectiveness of such interventions is important to consider and although this has generally been evaluated using observational methods, experimental approaches may also be feasible.

Given the increasing demand for and costs associated with health-care provision world-wide, findings from this evaluation may be of interest to other countries seeking to provide treatment that is both evidence-based and cost-effective. This includes countries already implementing preferred drug schemes (e.g. Australia), those which are considering such schemes or indeed any intervention aimed at changing clinical practice. The results show that initiatives which are primarily voluntary in nature may be impactful but their impact can be limited and short-term. They also show that interventions launched concurrently and developed using the same methodological framework may not necessarily yield similar results.

Conclusions

Since the introduction of the PDI in 2013, there have been some cost savings across the PDI drug classes. However, more intensive implementation is needed if the PDI is to deliver the estimated €15million per year cost saving that was anticipated. Multifaceted interventions will be required to enhance the coverage and impact of the PDI so that these benefits can be realised.

For peer review only - http://bmjopen.bmj.com/site/about/guidelines.xhtml

Acknowledgements: Thanks to the Health Services Executive Primary Care Reimbursement Scheme (HSE-PCRS) for the use of pharmacy claims database.

Contributors: RMcD drafted and planned all aspects of study design, cleaned and prepared data for analysis, conducted the statistical analyses and conducted a preliminary overview of the literature. KB prepared the monthly PCRS claims downloads and gave significant methodological guidance on the analysis strategy. FM provided guidance on pharmaceutical matters and contributed to the discussion on context, policy implications and future research. SC and MB facilitated access to the claims data with the PCRS, gave detailed information on roll-out and implementation of the Preferred Drugs Initiative, and contributed to interpretation of the results within the wider context of prescribing in Ireland. TF generated the research question and commented on the conduct, analysis and write-up of the paper. All authors read and approved submission of the paper.

Funding: The primary author was funded by the Health Research Board (HRB) of Ireland under the HRB Centre for Primary Care Phase 2 Funding award, grant HRC/2014/1. KB funded by HRB under grant RL-15-1579

Competing interests: None declared.

Data-sharing statement: No additional data are available.

For peer review only

References

1. Service HSEPCR. Primary Care reimbursement Service Statistical Analysis of Claims and Payments 2015. Available from: http://www.hse.ie/eng/staff/PCRS/PCRS_Publications/PCRS-statistical-analysis-of-claims-and-payments-2015.pdf.
2. Children HotOJCoHa. Report on the Cost of Prescription Drugs in Ireland 2015. 2015.
3. Brick A GP, Nolan A. Ireland: Pharmaceutical Prices, Prescribing Practices and Usage of Generics in a Comparative Context. Dublin: ESRI, 2013.
4. Layte R. (ed) BM, Bennett K, Brick A, Morgenroth E., Normand C, O'Reilly J, Thomas S, Tilson L, Wiley M, Wren M.-A. Demographic Change on the Demand for and Delivery of Health Care in Ireland. Dublin: ESRI, 2009.
5. Programme MM. Welcome to the Medicines Management Programme 2016. Available from: http://www.hse.ie/eng/about/Who/clinical/natclinprog/medicinemanagementprogramme/yourmedicines/.
6. Programme MM. Evaluation Reports. 2017.
7. von Elm E, Altman DG, Egger M, Pocock SJ, Gotzsche PC, Vandenbroucke JP, et al. The Strengthening the Reporting of Observational Studies in Epidemiology (STROBE) statement: guidelines for reporting observational studies. Journal of clinical epidemiology. 2008;61(4):344-9.
8. Sinnott SJ, Bennett K, Cahir C. Pharmacoepidemiology resources in Ireland-an introduction to pharmacy claims data. European journal of clinical pharmacology. 2017.
9. Bhaskaran K, Gasparrini A, Hajat S, Smeeth L, Armstrong B. Time series regression studies in environmental epidemiology. International journal of epidemiology. 2013;42(4):1187-95.
10. Rector TS, Finch MD, Danzon PM, Pauly MV, Manda BS. Effect of tiered prescription copayments on the use of preferred brand medications. Medical care. 2003;41(3):398-406.
11. Lopatto J, Keith SW, Del Canale S, Templin M, Maio V. Evaluating sustained quality improvements: long-term effectiveness of a physician-focused intervention to reduce potentially inappropriate medication prescribing in an older population. Journal of Clinical Pharmacy & Therapeutics. 2014;39(3):266-71.
12. Penfold RB, Zhang F. Use of interrupted time series analysis in evaluating health care quality improvements. Academic pediatrics. 2013;13(6 Suppl):S38-44.
13. Wagner AK, Soumerai SB, Zhang F, Ross-Degnan D. Segmented regression analysis of interrupted time series studies in medication use research. Journal of clinical pharmacy and therapeutics. 2002;27(4):299-309.
14. Education UIfDRa. How can I run a piecewise regression in Stata? 2017. Available from: https://stats.idre.ucla.edu/stata/faq/how-can-i-run-a-piecewise-regression-in-stata/.
15. Bernal J, Cummins, S, Gasparrini, A. Interrupted time series regression for the evaluation of public health interventions: a tutorial. International journal of epidemiology. 2017;46(1):384-55.
16. Prais SJ, and C. B. Winsten. Trend estimators and serial correlation: Working paper 383, Cowles Commission; 1954. Available from: http://cowles.econ.yale.edu/P/ccdp/st/s-0383.pdf.
17. StataCorp. Stata Statistical Software. Texas: StataCorp; 2015.
18. Davis D, O'Brien MA, Freemantle N, Wolf FM, Mazmanian P, Taylor-Vaisey A. Impact of formal continuing medical education: do conferences, workshops, rounds, and other traditional continuing education activities change physician behavior or health care outcomes? Jama. 1999;282(9):867-74.
19. Gill PS, Makela M, Vermeulen KM, Freemantle N, Ryan G, Bond C, et al. Changing doctor prescribing behaviour. Pharmacy world & science : PWS. 1999;21(4):158-67.
20. Godman B, Wettermark B, Miranda J, Bennie M, Martin A, Malmstrom RE. Influence of multiple initiatives in Sweden to enhance ARB prescribing efficiency following generic losartan; findings and implications for other countries. International journal of clinical practice. 2013;67(9):853-62.

For peer review only - http://bmjopen.bmj.com/site/about/guidelines.xhtml

21. Soumerai SB, Avorn J. Principles of educational outreach ('academic detailing') to improve clinical decision making. Jama. 1990;263(4):549-56.
22. Kuperman GJ, Bobb A, Payne TH, Avery AJ, Gandhi TK, Burns G, et al. Medication-related clinical decision support in computerized provider order entry systems: a review. Journal of the American Medical Informatics Association : JAMIA. 2007;14(1):29-40.
23. Persaud N, Ahmad H. Canadian list of essential medications: Potential and uncertainties. Canadian family physician Medecin de famille canadien. 2017;63(4):266-8.
24. Wettermark B, Pehrsson A, Jinnerot D, Bergman U. Drug utilisation 90% profiles--a useful tool for quality assessment of prescribing in primary health care in Stockholm. Pharmacoepidemiology and drug safety. 2003;12(6):499-510.
25. Eriksen J, Gustafsson LL, Ateva K, Bastholm-Rahmner P, Ovesjo ML, Jirlow M, et al. High adherence to the 'Wise List' treatment recommendations in Stockholm: a 15-year retrospective review of a multifaceted approach promoting rational use of medicines. BMJ open. 2017;7(4):e014345.
26. Health AGo. Therapeutic Group Premium Policy 2017. Available from: https://www.pbs.gov.au/browse/group-premium.
27. Sinnott SJ, Normand C, Byrne S, Woods N, Whelton H. Copayments for prescription medicines on a public health insurance scheme in Ireland. Pharmacoepidemiology and drug safety. 2016;25(6):695-704.
28. Huskamp HA, Deverka PA, Epstein AM, Epstein RS, McGuigan KA, Frank RG. The effect of incentive-based formularies on prescription-drug utilization and spending. The New England journal of medicine. 2003;349(23):2224-32.
29. Morgan SG, Leopold C, Wagner AK. Drivers of expenditure on primary care prescription drugs in 10 high-income countries with universal health coverage. CMAJ : Canadian Medical Association journal = journal de l'Association medicale canadienne. 2017;189(23):E794-E9.
30. Walley T, Murphy M, Codd M, Johnston Z, Quirke T. Effects of a monetary incentive on primary care prescribing in Ireland: changes in prescribing patterns in one health board 1990-1995. Pharmacoepidemiology and drug safety. 2000;9(7):591-8.
31. Roland M. Incentives must be closely aligned to professional values. Bmj. 2012;345:e5982.
32. Majumdar SR, Soumerai SB. Why most interventions to improve physician prescribing do not seem to work. CMAJ : Canadian Medical Association journal = journal de l'Association medicale canadienne. 2003;169(1):30-1.
33. Persaud N, Lee T, Ahmad H, Li W, Taglione MS, Rajakulasingam Y, et al. Protocol for a randomised controlled trial evaluating the effects of providing essential medicines at no charge: the Carefully seLected and Easily Accessible at No Charge Medicines (CLEAN Meds) trial. BMJ open. 2017;7(5):e015686.

For peer review only

Table 1:Summary of impact of Preferred Drugs Initiative (2011-2016)

Preferred drug class	PPI	Statin	ACE	ARB	SNRI	SSRI	urology	Total
Total no. items	18,939,282	19,944,634	8,837,006	5,171,204	3,345,307	8,348,567	2,239,263	336,535,263
% of all drugs	5.63%	5.93%	2.63%	1.54%	0.99%	2.48%	0.67%	19.86%
**Preferred drug**	**lansoprazole**	**simvastatin**	**ramipril**	**candesartan**	**venlafaxine**	**citalopram**	**ER tolterodine**	
**Total no. single-agent items**	4,571,751	1,313,389	4,719,996	557,622	1,155,600	1,650,520	577,540	
**% within class**	24.14%	6.59%	53.41%	10.78%	70.99%	19.77%	25.79%	
**Rank within class pre-PDI**	2/5	4/5	1/10	5/8	1/2	2/6	1/9	
**Rank within class post-PDI**	2/5	4/5	1/10	5/8	1/2	3/6	3/9	
**Absolute change in proportion of preferred drug items: first 3 months post-PDI v previous 3 months**	↑ +0.98% (p<0.001)	↑ +0.30% (p<0.001)	↑ 0.53% (p<0.001)	↓ -0.03% (p=0.76)	↑ 0.30% (p=0.08)	↓ -0.09% (p=0.37)	↓ -0.98% (p<0.001)	

PPI: proton pump inhibitor; ACE: angiotensin-converting enzyme (ACE) inhibitor; ARB: angiotensin-II receptor blocker; SNRI: serotonin and noradrenaline reuptake inhibitor ; SSRI: selective serotonin receptor antagonist; ER: Extended Release; PDI: Preferred Drugs Initiative

For peer review only - http://bmjopen.bmj.com/site/about/guidelines.xhtml

Table 2: Segmented regression analysis in relation to PDI guideline publication, class-specific changes and cost savings

**Medicine group (Preferred drug)**	**Guidelines introduced**	**Percentage of preferred drug items: Jan-March 2011 (SE), 95% CI**	**Increase in % of preferred drug items per quarter post March 2011 (SE), 95%CI, p-value**	**Increase in % of preferred drug items Jan-Mar 2013 following licensing of mirabegron, (SE), 95%CI, p-value**	**Increase in % of preferred drug items per quarter post March 2013 (SE), 95%CI, p-value**	**Increase in % of preferred drug items quarter immediately following PDI guidelines, (SE), 95%CI, p-value**	**Increase in % of preferred drug items per quarter post PDI guidelines, (SE), 95%CI, p-value**	**Increase in % of preferred drug April-June 2015 following introduction of generic duloxetine, (SE), 95%CI, p-value**	**Increase in % of preferred drug items per quarter post June 2015 , (SE), 95%CI, p-value**	**Estimated savings between issuing of guidelines and Dec 2016 (€)**
PPIs (lansoprazole)	April 2013	24.53 (0.47), (23.59,25.47)	-0.21 (0.05), (-0.32,-0.11), p=0.001	-	-	1.21 (0.18), (0.84,1.57), p<0.001	0.04 (0.04) (-0.03,0.12), p=0.25	-	-	618,158
Statins (simvastatin)	April 2013	5.94 (0.21), (5.50,6.38)	0.02 (0.03), (-0.04,0.07), p=0.54	-	-	0.30 (0.10), (0.10,0.50), p=0.01	0.07 (0.02), (0.03,0.10), p=0.002	-	-	363,194
ACEs (ramipril)	September 2013	49.14 (0.07), (48.99,49.28)	0.38 (0.01). (0.35,0.40), p<0.001	-	-	0.16 (0.07), (0.01,0.31), p=0.04	0.41 (0.01), (0.39,0.42), p<0.001	-	-	50,163
ARBs (candesartan)	September 2013	11.90 (0.08), (11.73,12.07)	-0.15 (0.01), (-0.17,-0.12), p<0.001	-	-	0.15 (0.06), (0.02,0.29), p=0.03	0.01 (0.01), (-0.01,0.03), p=0.46	-	-	132,625
SNRIs (venlafaxine)	April 2014	73.61 (0.44), (72.69,74.53)	-0.35 (0.05), (-0.46,-0.24), p<0.001	-	-	0.71 (0.27), (0.15,1.27), p=0.02	0.26 (0.13), (-0.02,0.55), p=0.07	-0.09 (0.30), (-0.73,0.54), p=0.76	-0.08 (0.09), (-0.10,0.26), p=0.34	1,291,160
SSRIs (citalopram)	April 2014	23.58 (0.13), (23.31,23.85)	-0.36 (0.01), (-0.39,-0.33), p<0.001	-	-	0.30 (0.08), (0.12,0.47), p=0.002	-0.23 (0.02), (-0.27,-0.19), p<0.001	-	-	169,493
urology (ER tolterodine)	October 2014	37.27 (0.27), (36.69,37.84)	-1.00 (0.05), (-1.11,-0.88), p<0.001	0.16 (0.24), (-0.35,0.66), p=0.52	-1.04 (0.06), (-1.17,-0.91), p<0.001	-0.06 (0.24), (-0.57,0.45), p=0.82	-0.63 (0.09), (-0.73,-0.52), p<0.001	-	-	46,695
**Total savings**										**2,671,447**

For peer review only - http://bmjopen.bmj.com/site/about/guidelines.xhtml

CI: Confidence Interval; SE: Standard Error; ER: Extended Release; PPI: proton pump inhibitor; ACE: angiotensin-converting enzyme (ACE) inhibitor; ARB: angiotensin-II receptor blocker; SNRI: serotonin and noradrenaline reuptake inhibitor ; SSRI: selective serotonin receptor antagonist; ER: Extended Release; PDI: Preferred Drugs Initiative

For peer review only

For peer review only - http://bmjopen.bmj.com/site/about/guidelines.xhtml

Table 3: Segmented regression analysis in relation to PDI guideline publication, reference groups

**Medicine group (Preferred drug)**	**Guidelines introduced**	**Percentage of preferred drug items: Jan-March 2011 (SE), 95% CI**	**Increase in % of preferred drug items per quarter post March 2011 (SE), 95%CI, p-value**	**Increase in % of preferred drug items April-June 2013†, (SE), 95%CI, p-value**	**Increase in % of preferred drug items per quarter post June 2013, (SE), 95%CI, p-value**	**Increase in % of preferred drug items Oct-Dec 2013††, (SE), 95%CI, p-value**	**Increase in % of preferred drug items per quarter post Dec 2013, (SE), 95%CI, p-value**	**Increase in % of preferred drug items April-June 2014*, (SE), 95%CI, p-value**	**Increase in % of preferred drug items per quarter post June 2014, (SE), 95%CI, p-value**	**Increase in % of preferred drug items Oct-Dec 2014** (SE), 95%CI, p-value**	**Increase in % of preferred drug items per quarter post Dec 2014, (SE), 95%CI, p-value**
beta-blockers (bisoprolol)	September 2016	51.20 (0.03), (51.15,51.26)	0.53 (0.01), (0.52,0.54), p<0.001	-0.02 (0.05), (-0.13,0.09), p=0.71	0.50 (0.02), (0.45,0.54), p<0.001	-	-	-0.05 (0.06), (-0.18,0.08), p=0.44	0.41 (0.001), (0.40,0.42), p<0.001	-	-
calcium channel blockers (amlodipine)	September 2016	68.18 (0.03), (68.12,68.29)	-0.34 (0.01), (-0.35,-0.33), p<0.001	-	-	0.12 (0.06), (-0.001,0.23), p=0.06	-0.26 (0.02) (-0.31,-0.21), p<0.001	-	-	0.02 (0.07), (-0.13,0.17), p=0.76	-0.21 (0.01) (-0.22,-0.19), p<0.001

† introduction of PDI guidelines for PPIs/statins; †† introduction of PDI guidelines for ACE/ARBs; * introduction of PDI guidelines for SNRIs/SSRIs; ** introduction of PDI guidelines for urology medications; CI: Confidence Interval; SE: Standard Error; ER: Extended Release; PDI: Preferred Drugs Initiative

For peer review only

For peer review only - http://bmjopen.bmj.com/site/about/guidelines.xhtml

Figure Legends

Figure 1: Distribution of preferred drug items by therapeutic drug class

Figure 2: Estimated percentage of preferred drugs by drug class: segmented regression models

For peer review only

For peer review only - http://bmjopen.bmj.com/site/about/guidelines.xhtml

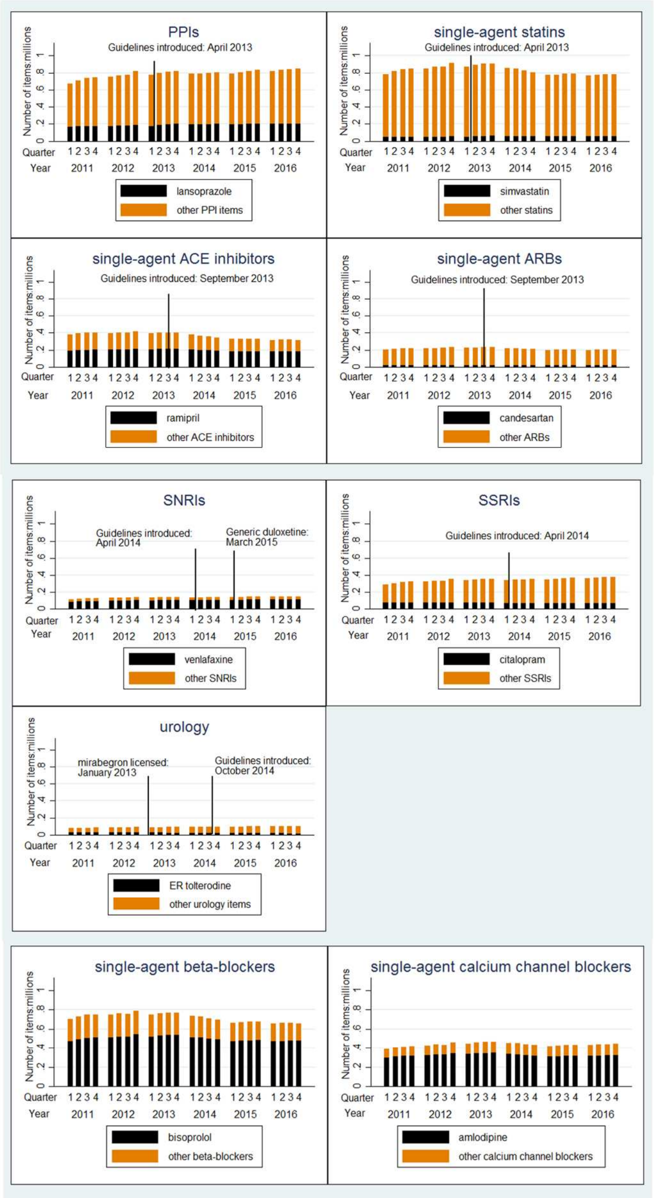

Figure 1: Distribution of preferred drug items by therapeutic drug class

159x290mm (300 x 300 DPI)

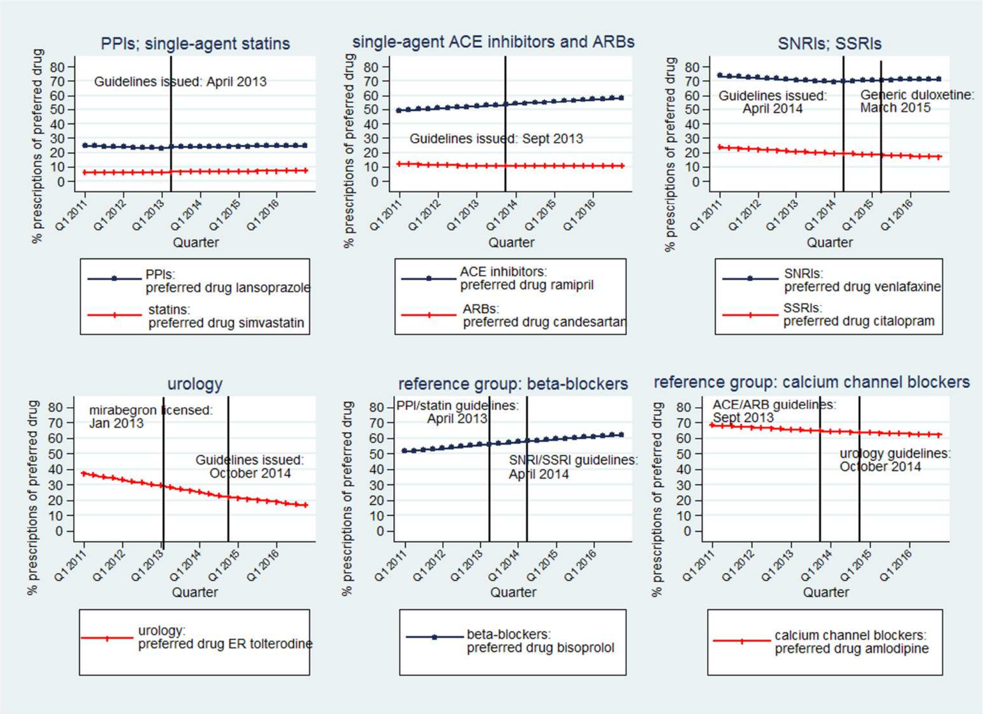

Figure 2: Estimated percentage of preferred drugs by drug class: segmented regression models

180x129mm (300 x 300 DPI)

Appendix

Table A1: Breakdown of PCRS reimbursed items: 2011-2016

Year	No. items issued	No. single-agent items issued across 7 drug classes*	% of items attributed to 7 drug classes*	No. items issued for preferred drugs	% preferred drug items within preferred drug classes	% preferred drug items across all prescriptions
**2011**	54,324,492	10,630,476	19.57%	2,520,986	23.71%	4.64%
**2012**	57,984,934	11,380,582	19.63%	2,641,897	23.21%	4.56%
**2013**	58,455,927	11,640,615	19.91%	2,708,855	23.27%	4.63%
**2014**	55,978,157	11,181,081	19.97%	2,655,422	23.75%	4.74%
**2015**	54,573,162	10,925,162	20.02%	2,610,926	23.90%	4.78%
**2016**	55,218,591	11,067,347	20.04%	2,627,631	23.74%	4.76%
**Total**	336,553,263	66,825,263	19.86%	15,765,717	23.59%	4.68%

***PPIs, Statins, ACEs, ARBs, SNRIs, SSRIs, Urology**

Table A2 Prevalence of PCRS reimbursed items by therapeutic drug class (single agent drugs)

	PPIs	statins	ACEs	ARBs	SNRIs	SSRIs	urology	Other	Total
**Year**									
**2011**	2,860,986 (5.27%)	3,286,352 (6.05%)	1,586,992 (2.92%)	849,807 (1.56%)	470,234 (0.87%)	1,247,643 (2.30%)	328,462 (0.60%)	43,694,016 (80.43%)	54,324,492 (100%)
**2012**	3,114,214 (5.37%)	3,501,257 (6.04%)	1,616,612 (2.79%)	899,594 (1.55%)	537,800 (0.93%)	1,355,921 (2.34%)	355,184 (0.61%)	46,604,352 (80.37%)	57,984,934 (100%)
**2013**	3,203,104 (5.48%)	3,582,112 (6.13%)	1,595,582 (2.73%)	920,851 (1.58%)	566,951 (0.97%)	1,404,466 (2.40%)	367,549 (0.63%)	46,815,312 (80.09%)	58,455,927 (100%)
**2014**	3,180,702 (5.68%)	3,339,227 (5.97%)	1,449,173 (2.59%)	867,567 (1.55%)	567,859 (1.01%)	1,399,724 (2.50%)	376,829 (0.67%)	44,797,076 (80.03%)	55,978,157 (100%)
**2015**	3,241,661 (5.94%)	3,129,117 (5.73%)	1,312,155 (2.40%)	816,250 (1.50%)	588,689 (1.08%)	1,441,270 (2.64%)	396,020 (0.73%)	43,648,000 (79.98%)	54,573,162 (100%)
**2016**	3,338,615 (6.05%)	3,106,569 (5.63%)	1,276,492 (2.31%)	817,135 (1.48%)	613,774 (1.11%)	1,499,543 (2.72%)	415,219 (0.75%)	44,151,244 (79.96%)	55,218,591 (100%)
**Total**	18,939,282 (5.63%)	19,944,634 (5.93%)	8,837,006 (2.63%)	5,171,204 (1.54%)	3,345,307 (0.99%)	8,348,567 (2.48%)	2,239,263 (0.67%)	269,710,000 (80.14%)	336,535,263 (100%)

Fig A1: Breakdown of PCRS reimbursed items by preferred drug status: 2011-2016

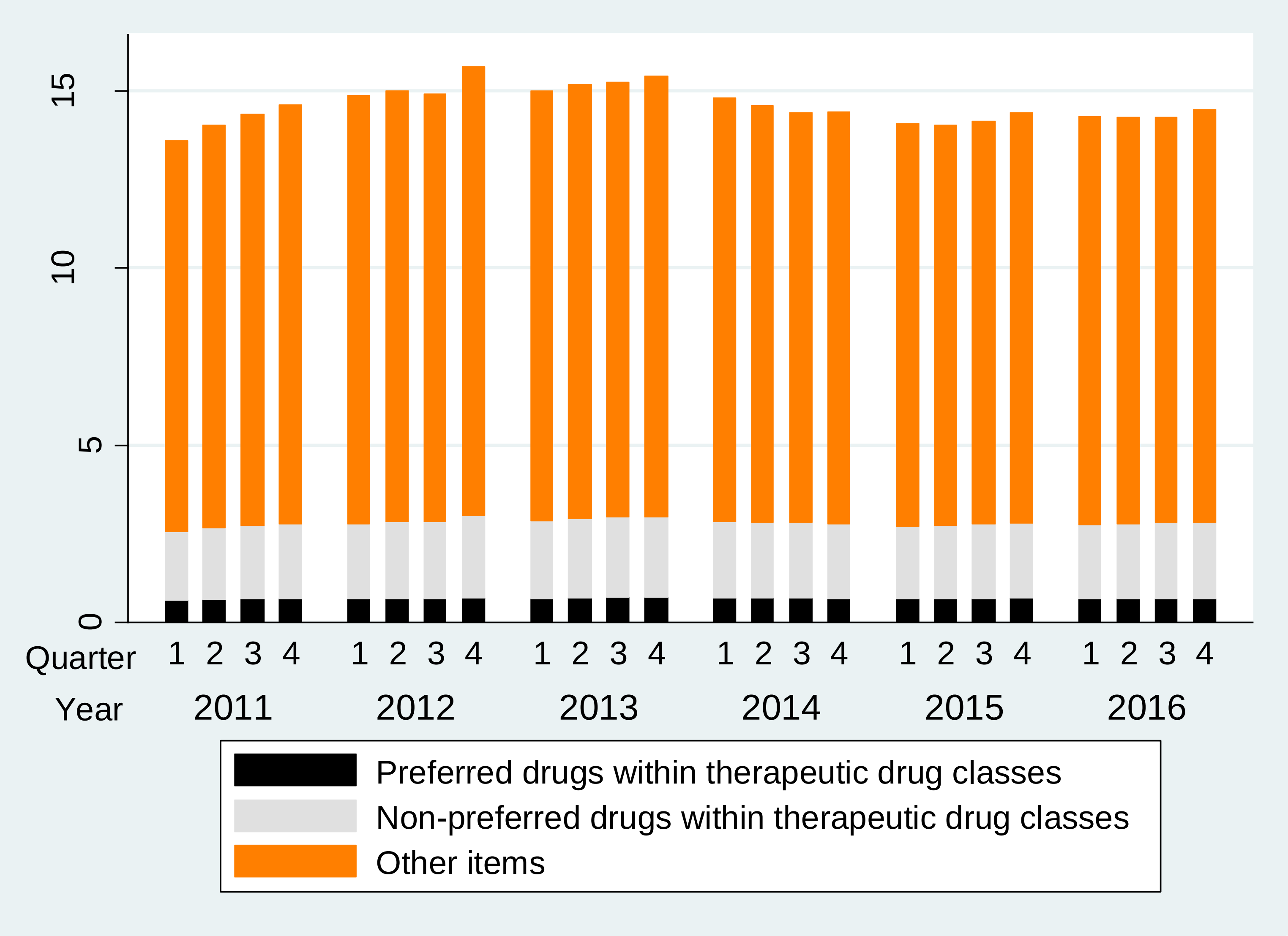

Fig A2: Observed percentage of preferred drugs by drug class

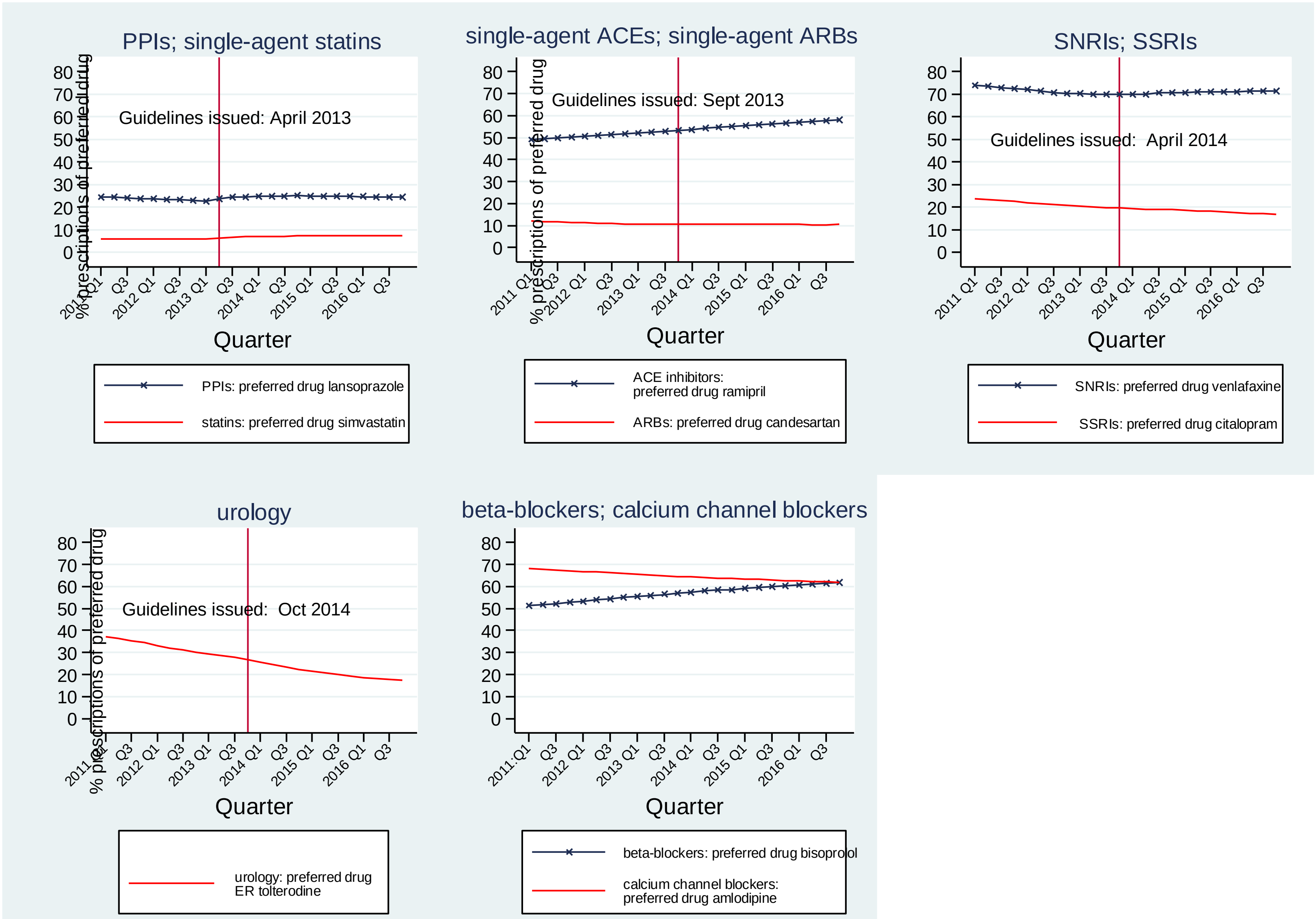

Table A3: Sensitivity analyses for segmented regression models

	Calendar quarters retained for analysis (Study Period)			
	**All available data: 9 quarters before guidelines, 15 quarters after guidelines, (Jan 11- Dec 16)**	**9 quarters before guidelines, 13 quarters after guidelines (Jan 11-Jun 16)**	**9 quarters before guidelines, 11 quarters after guidelines (Jan 11-Dec 15)**	**9 quarters before guidelines, 9 quarters after guidelines (Jan 11-Jun 15)**
**lansoprazole**				
Percentage of preferred drug items: beginning of study period (SE), 95% CI	24.53 (0.47), (23.59,25.47)	24.51 (0.40), (23.66,25.36)	24.47 (0.29), (23.85,25.09)	24.42 (0.19), (24.02,24.83)
Increase in % of preferred drug items per quarter following commencement of study period (SE), 95%CI, p-value	-0.21 (0.05), (-0.32,-0.11), p=0.001	-0.21 (0.05), (-0.32,-0.10), p=0.001	-0.21 (0.04), (-0.30,-0.11), p<0.001	-0.21 (0.04), (-0.28,-0.14), p<0.001
Increase in % of preferred drug items quarter immediately following PDI guidelines, (SE), 95%CI, p-value	1.21 (0.18), (0.84,1.57), p<0.001	1.21 (0.18), (0.83,1.59), p<0.001	1.22 (0.17), (0.85,1.59), p<0.001	1.26 (0.16), (0.90,1.61), p<0.001
Increase in % of preferred drug items per quarter post PDI guidelines, (SE), 95%CI, p-value	0.04 (0.04), (-0.03,0.12), p=0.25	0.06 (0.04), (-0.02,0.14), p=0.14	0.09 (0.04) (0.02,0.18), p=0.01	0.14 (0.03) (0.08,0.22), p<0.001
**simvastatin**				
Percentage of preferred drug items: beginning of study period (SE), 95% CI	5.94 (0.21), (5.50,6.38)	5.92 (0.17), (5.56,6.27)	5.89 (0.12), (5.63,6.15)	5.87 (0.07), (5.73,6.01)
Increase in % of preferred drug items per quarter following commencement of study period (SE), 95%CI, p-value	0.02 (0.03), (-0.04,0.07), p=0.54	0.02 (0.03), (-0.03,0.07), p=0.49	0.02 (0.02), (-0.03,0.07), p=0.32	0.02 (0.02), (-0.02,0.04), p=0.27
Increase in % of preferred drug items quarter immediately following PDI guidelines, (SE), 95%CI, p-value	0.30 (0.10), (0.10,0.50), p=0.01	0.30 (0.10), (0.10,0.51), p=0.01	0.32 (0.10), (0.12,0.52), p=0.01	0.42 (0.09), (0.23,0.62), p<0.001
Increase in % of preferred drug items per quarter post PDI guidelines, (SE), 95%CI, p-value	0.07 (0.02), (0.03,0.10), p=0.002	0.08 (0.02), (0.04,0.12), p=0.001	0.09 (0.02), (0.06,0.13), p<0.001	0.10 (0.01), (0.07,0.13), p<0.001

Table A3 (cont): sensitivity analyses for segmented regression models

	Calendar quarters retained for analysis (Study Period)					
	**All available data: 11 quarters before guidelines, 13 quarters after guidelines (Jan 11-Dec 16)**	**11 quarters before guidelines, 11 quarters after guidelines (Jan 11-Jun 16)**	**11 quarters before guidelines, 9 quarters after guidelines (Jan 11-Dec 15)**	**9 quarters before guidelines, 13 quarters after guidelines (Jul 11-Dec 16)**	**9 quarters before guidelines, 11 quarters after guidelines (Jul 11-Jun 16)**	**9 quarters before guidelines, 9 quarters after guidelines (Jul 11-Jan 15)**
**ramipril**						
Percentage of preferred drug items: beginning of study period (SE), 95% CI	49.14 (0.07), (48.99,49.28)	49.14 (0.08), (48.97,49.30)	49.14 (0.07), (48.98,49.28)	49.93 (0.07), (49.78,50.08)	49.92 (0.07), (49.76,50.10)	49.94 (0.07), (49.78,50.10)
Increase in % of preferred drug items per quarter following commencement of study period (SE), 95%CI, p-value	0.38 (0.01), (0.35,0.40), p<0.001	0.38 (0.02), (0.35,0.40), p<0.001	0.38 (0.02). (0.35,0.40), p<0.001	0.37 (0.01), (0.34,0.40), p<0.001	0.37 (0.01). (0.34,0.40), p<0.001	0.37 (0.01). (0.33,0.40), p<0.001
Increase in % of preferred drug items quarter immediately following PDI guidelines, (SE), 95%CI, p-value	0.16 (0.07), (0.01,0.31), p=0.04	0.16 (0.07), (0.01,0.32), p=0.04	0.16 (0.08), (-0.01,0.33), p=0.04	0.16 (0.07), (0.02,0.34), p=0.03	0.17 (0.08), (0.01,0.35), p=0.04	0.18 (0.09), (-0.01,0.35), p=0.06
Increase in % of preferred drug items per quarter post PDI guidelines, (SE), 95%CI, p-value	0.41 (0.01), (0.39,0.42), p<0.001	0.40 (0.01), (0.38,0.43), p<0.001	0.41 (0.01), (0.38,0.44), p<0.001	0.41 (0.01), (0.39,0.42), p<0.001	0.40 (0.01), (0.38,0.43), p<0.001	0.41 (0.01), (0.38,0.44), p<0.001
**candesartan**						
Percentage of preferred drug items: beginning of study period (SE), 95% CI	11.90 (0.08), (11.73,12.07)	11.90 (0.09), (11.71,12.09)	11.89 (0.08), (11.73,12.06)	11.60 (0.09), (11.42,11.80)	11.61 (0.11), (11.40,11.84)	11.60 (0.08), (11.41,11.78)
Increase in % of preferred drug items per quarter following commencement of study period (SE), 95%CI, p-value	-0.15 (0.01), (-0.17,-0.12), p<0.001	-0.15 (0.01), (-0.17,-0.12), p<0.001	-0.16 (0.01), (-0.17,-0.12), p<0.001	-0.15 (0.01), (-0.17,-0.12), p<0.001	-0.15 (0.01), (-0.18,-0.12), p<0.001	-0.15 (0.01), (-0.18,-0.12), p<0.001
Increase in % of preferred drug items quarter immediately following PDI guidelines, (SE), 95%CI, p-value	0.15 (0.06), (0.02,0.29), p=0.03	0.14 (0.06), (0.01,0.29), p=0.04	0.16 (0.06), (0.02,0.30), p=0.03	0.14 (0.07), (0.01,0.28), p=0.04	0.14 (0.07), (-0.01,0.28), p=0.06	0.15 (0.06), (0.01,0.29), p=0.05
Increase in % of preferred drug items per quarter post PDI guidelines, (SE), 95%CI, p-value	0.01 (0.01), (-0.01,0.03), p=0.46	0.01 (0.01), (-0.02,0.03), p=0.75	0.02 (0.01), (-0.01,0.05), p=0.25	0.01 (0.01), (-0.01,0.03), p=0.49	0.01 (0.01), (-0.03,0.03), p=0.82	0.01 (0.01), (-0.01,0.05), p=0.31

Table A3 (cont): sensitivity analyses for segmented regression models

	Calendar quarters retained for analysis (Study Period)					
	**All available data: 13 quarters before guidelines, 11 quarters after guidelines (Jan 11-Dec 16)**	**13 quarters before guidelines, 9 quarters after guidelines (Jan 11-Jun 16)**	**11 quarters before guidelines, 11 quarters after guidelines (Jul 11-Dec 16)**	**11 quarters before guidelines, 9 quarters after guidelines (Jul 11-Jun 16)**	**9 quarters before guidelines, 11 quarters after guidelines (Jan 12-Dec 16)**	**9 quarters before guidelines, 9 quarters after guidelines (Jan 12-Jun 16)**
**venlafaxine**						
Percentage of preferred drug items: beginning of study period (SE), 95% CI	73.61 (0.44), (72.69,74.53)	73.61 (0.46), (72.63,74.60)	72.56 (0.35), (71.81,73.31)	72.57(0.38), (71.75,73.40)	71.45 (0.22), (70.98,71.91)	71.46 (0.24), (70.94,71.98)
Increase in % of preferred drug items per quarter following commencement of study period (SE), 95%CI, p-value	-0.35 (0.05), (-0.46,-0.24), p<0.001	-0.35 (0.05), (-0.46,-0.24), p<0.001	-0.32 (0.05), (-0.43,-0.21), p<0.001	-0.32 (0.05), (-0.43,-0.20), p<0.001	-0.25 (0.04), (-0.34,-0.16), p<0.001	-0.25 (0.05), (-0.35,-0.14), p<0.001
Increase in % of preferred drug items quarter immediately following PDI guidelines, (SE), 95%CI, p-value	0.71 (0.27), (0.15,1.27), p=0.02	0.71 (0.28), (0.12,1.29), p=0.02	0.71 (0.28), (0.12,1.31), p=0.02	0.70 (0.29), (0.08,1.32), p=0.03	0.79 (0.29), (0.16,1.42), p=0.02	0.78 (0.31), (0.10,1.45), p=0.03
Increase in % of preferred drug items per quarter post PDI guidelines, (SE), 95%CI, p-value	0.26 (0.13), (-0.02,0.55), p=0.07	0.26 (0.14), (-0.04,0.55), p=0.08	0.27 (0.13), (-0.01,0.56), p=0.05	0.26 (0.14), (-0.02,0.57), p=0.08	0.26 (0.12), (0.01,0.52), p=0.04	0.26 (0.13), (-0.02,0.53), p=0.07
Increase in % of preferred drug April-June 2015 following introduction of generic duloxetine, (SE), 95%CI, p-value	-0.09 (0.30), (-0.73,0.54), p=0.76	-0.09 (0.31), (-0.76,0.57), p=0.77	-0.11 (0.32), (-0.79,0.57), p=0.74	-0.11 (0.33), (-0.82,0.60), p=0.75	-0.15 (0.35), (-0.89,0.60), p=0.68	-0.17 (0.37), (-0.97,0.64), p=0.66
Increase in % of preferred drug items per quarter post June 2015 , (SE), 95%CI, p-value	-0.08 (0.09), (-0.10,0.26), p=0.34	0.14 (0.12), (-0.10,0.39), p=0.24	-0.08 (0.08), (-0.09,0.26), p=0.34	0.14 (0.11), (-0.10,0.39), p=0.26	0.07 (0.06), (-0.06,0.20), p=0.27	0.10 (0.09), (-0.10,0.33), p=0.27
**citalopram**						
Percentage of preferred drug items: beginning of study period (SE), 95% CI	23.58 (0.13), (23.31,23.85)	23.58 (0.12), (23.32,23.83)	22.88 (0.14), (22.89,23.17)	22.87 (0.13), (22.59,23.14)	21.95 (0.08), (21.78,22.12)	21.93 (0.06), (21.81,22.04)
Increase in % of preferred drug items per quarter following commencement of study period (SE), 95%CI, p-value	-0.36 (0.01), (-0.39,-0.33), p<0.001	-0.36 (0.01), (-0.39,-0.33), p<0.001	-0.36 (0.02), (-0.40,-0.32), p<0.001	-0.36 (0.02), (-0.40,-0.33), p<0.001	-0.33 (0.01), (-0.36,-0.30), p<0.001	-0.33 (0.01), (-0.36,-0.31), p<0.001
Increase in % of preferred drug items quarter immediately following PDI guidelines, (SE), 95%CI, p-value	0.30 (0.08), (0.12,0.47), p=0.002	0.30 (0.08), (0.12,0.48), p=0.003	0.30 (0.09), (0.11,0.48), p=0.003	0.30 (0.09), (0.11,0.50), p=0.005	0.30 (0.08), (0.13,0.47), p=0.002	0.34 (0.08), (0.17,0.51), p=0.001
Increase in % of preferred drug items per quarter post PDI guidelines, (SE), 95%CI, p-value	-0.23 (0.02), (-0.27,-0.19), p<0.001	-0.22 (0.02), (-0.26,-0.18), p<0.001	-0.23 (0.02), (-0.27,-0.19), p<0.001	-0.22 (0.02), (-0.27,-0.17), p<0.001	-0.24 (0.02), (-0.26,-0.21), p<0.001	-0.23 (0.01), (-0.25,-0.20), p<0.001

Table A3 (cont): sensitivity analyses for segmented regression models

	Calendar quarters retained for analysis (Study Period)			
	**All available data: 15 quarters before guidelines, 9 quarters after guidelines (Jan 11-Dec 16)**	**13 quarters before guidelines, 9 quarters after guidelines (Jul 11-Dec 16)**	**11 quarters before guidelines, 9 quarters after guidelines (Jan 12-Dec 16)**	**9 quarters before guidelines, 9 quarters after guidelines (Jul 12-Dec 16)**
**ER tolterodine**				
Percentage of preferred drug items: beginning of study period (SE), 95% CI	37.27 (0.27), (36.69,37.84)	35.45 (0.30), (34.81,36.09)	33.16 (0.33), (32.46,33.87)	31.10 (0.39) (30.29,31.93)
Increase in % of preferred drug items per quarter following commencement of study period (SE), 95%CI, p-value	-1.00 (0.05), (-1.11,-0.88), p<0.001	-1.04 (0.07), (-1.21,-0.88), p<0.001	-0.97 (0.11), (-1.21,-0.73), p<0.001	-0.98 (0.06), (-1.11,-0.86), p<0.001
Increase in % of preferred drug items Jan-Mar 2013 following licensing of mirabegron, (SE), 95%CI, p-value	0.16 (0.24), (-0.35,0.66), p=0.52	0.21 (0.26), (-0.34,0.75), p=0.43	0.11 (0.25), (-0.44,0.65), p=0.68	*
Increase in % of preferred drug items per quarter post March 2013 (SE), 95%CI, p-value	-1.04 (0.06), (-1.17,-0.91), p<0.001	-1.03 (0.07), (-1.17,-0.89), p<0.001	-1.03 (0.06), (-1.17,-0.89), p<0.001	*
Increase in % of preferred drug items quarter immediately following PDI guidelines, (SE), 95%CI, p-value	-0.06 (0.24), (-0.57,0.45), p=0.82	-0.05 (0.25), (-0.57,0.49), p=0.86	-0.01 (0.24), (-0.51,0.49), p=0.96	-0.05 (0.22), (-0.52,0.43), p=0.86
Increase in % of preferred drug items per quarter post PDI guidelines, (SE), 95%CI, p-value	-0.63 (0.09), (-0.73,-0.52), p<0.001	-0.63 (0.06), (-0.73,-0.51), p<0.001	-0.62 (0.05), (-0.73,-0.51), p<0.001	-0.63 (0.06), (-0.75,-0.50), p<0.001

*omitted due to close proximity of study period (July 2012) and licensing of mirabegron (Jan 2013)

Table A4: Sensitivity analyses: alternative definition of calendar quarters for ACE inhibitors/ARBs

	**Calendar quarters: Jan-Mar, Apr-Jun, Jul-Sep, Oct-Dec (24 calendar quarters: Jan 11-Dec 16)**	**Calendar quarters: Mar-May, Jun-Aug, Sep-Nov, Dec-Feb (23 calendar quarters: Mar 11-Nov 16)**
**ramipril**		
Percentage of preferred drug items: beginning of study period (SE), 95% CI	49.14 (0.07), (48.99,49.28)	49.25 (0.07), (49.27, 49.58)
Increase in % of preferred drug items per quarter following commencement of study period (SE), 95%CI, p-value	0.38 (0.01), (0.35,0.40), p<0.001	0.37 (0.01), (0.34,0.40), p<0.001
Increase in % of preferred drug items quarter immediately following PDI guidelines, (SE), 95%CI, p-value	0.16 (0.07), (0.01,0.31), p=0.04	0.14 (0.08), (-0.01,0.30), p=0.05
Increase in % of preferred drug items per quarter post PDI guidelines, (SE), 95%CI, p-value	0.41 (0.01), (0.39,0.42), p<0.001	0.41 (0.01), (0.39,0.43), p<0.001
**candesartan**		
Percentage of preferred drug items: beginning of study period (SE), 95% CI	11.90 (0.08), (11.73,12.07)	11.78 (0.07), (11.63,11.92)
Increase in % of preferred drug items per quarter following commencement of study period (SE), 95%CI, p-value	-0.15 (0.01), (-0.17,-0.12), p<0.001	-0.15 (0.01), (-0.17,-0.13), p<0.001
Increase in % of preferred drug items quarter immediately following PDI guidelines, (SE), 95%CI, p-value	0.15 (0.06), (0.02,0.29), p=0.03	0.17 (0.06), (0.06,0.29), p=0.01
Increase in % of preferred drug items per quarter post PDI guidelines, (SE), 95%CI, p-value	0.01 (0.01), (-0.01,0.03), p=0.46	0.01 (0.01), (-0.02,0.02), p=0.90

STROBE 2007 (v4) checklist of items to be included in reports of observational studies in epidemiology*
Checklist for cohort, case-control, and cross-sectional studies (combined)

Section/Topic	Item #	Recommendation	Reported on page #
**Title and abstract**	1	(*a*) Indicate the study's design with a commonly used term in the title or the abstract	5
		(*b*) Provide in the abstract an informative and balanced summary of what was done and what was found	2
**Introduction**			
Background/rationale	2	Explain the scientific background and rationale for the investigation being reported	4
Objectives	3	State specific objectives, including any pre-specified hypotheses	4
**Methods**			
Study design	4	Present key elements of study design early in the paper	5
Setting	5	Describe the setting, locations, and relevant dates, including periods of recruitment, exposure, follow-up, and data collection	5
Participants	6	(*a*) *Cohort study*—Give the eligibility criteria, and the sources and methods of selection of participants. Describe methods of follow-up<br>*Case-control study*—Give the eligibility criteria, and the sources and methods of case ascertainment and control selection. Give the rationale for the choice of cases and controls<br>*Cross-sectional study*—Give the eligibility criteria, and the sources and methods of selection of participants	5
		(*b*) *Cohort study*—For matched studies, give matching criteria and number of exposed and unexposed<br>*Case-control study*—For matched studies, give matching criteria and the number of controls per case	
Variables	7	Clearly define all outcomes, exposures, predictors, potential confounders, and effect modifiers. Give diagnostic criteria, if applicable	5
Data sources/ measurement	8*	For each variable of interest, give sources of data and details of methods of assessment (measurement). Describe comparability of assessment methods if there is more than one group	*5*
Bias	9	Describe any efforts to address potential sources of bias	10-all available data used
Study size	10	Explain how the study size was arrived at	5-all available data used
Quantitative variables	11	Explain how quantitative variables were handled in the analyses. If applicable, describe which groupings were chosen and why	5-groupings as per medicine group
Statistical methods	12	(*a*) Describe all statistical methods, including those used to control for confounding	5/6
		(*b*) Describe any methods used to examine subgroups and interactions	n/a

For peer review only

		(*c*) Explain how missing data were addressed	n/a
		(*d*) *Cohort study*—If applicable, explain how loss to follow-up was addressed<br>*Case-control study*—If applicable, explain how matching of cases and controls was addressed<br>*Cross-sectional study*—If applicable, describe analytical methods taking account of sampling strategy	n/a
		(*e*) Describe any sensitivity analyses	6, Appendix
**Results**			
Participants	13*	(a) Report numbers of individuals at each stage of study—eg numbers potentially eligible, examined for eligibility, confirmed eligible, included in the study, completing follow-up, and analysed	7
		(b) Give reasons for non-participation at each stage	n/a
		(c) Consider use of a flow diagram	N/A
Descriptive data	14*	(a) Give characteristics of study participants (eg demographic, clinical, social) and information on exposures and potential confounders	N/A
		(b) Indicate number of participants with missing data for each variable of interest	N/A
		(c) *Cohort study*—Summarise follow-up time (eg, average and total amount)	All data 2011-2016 used
Outcome data	15*	*Cohort study*—Report numbers of outcome events or summary measures over time	N/a
		Case-control study—Report numbers in each exposure category, or summary measures of exposure	N/A
		Cross-sectional study—Report numbers of outcome events or summary measures	7, Table 1
Main results	16	(*a*) Give unadjusted estimates and, if applicable, confounder-adjusted estimates and their precision (eg, 95% confidence interval). Make clear which confounders were adjusted for and why they were included	7,8
		(*b*) Report category boundaries when continuous variables were categorized	n/a
		(*c*) If relevant, consider translating estimates of relative risk into absolute risk for a meaningful time period	8
Other analyses	17	Report other analyses done—eg analyses of subgroups and interactions, and sensitivity analyses	8, Appendix
**Discussion**			
Key results	18	Summarise key results with reference to study objectives	9
Limitations	19	Discuss limitations of the study, taking into account sources of potential bias or imprecision. Discuss both direction and magnitude of any potential bias	10
Interpretation	20	Give a cautious overall interpretation of results considering objectives, limitations, multiplicity of analyses, results from similar studies, and other relevant evidence	9/10
Generalisability	21	Discuss the generalisability (external validity) of the study results	9/10
**Other information**			
Funding	22	Give the source of funding and the role of the funders for the present study and, if applicable, for the original study on which the present article is based	12

For peer review only

*Give information separately for cases and controls in case-control studies and, if applicable, for exposed and unexposed groups in cohort and cross-sectional studies.
Note: An Explanation and Elaboration article discusses each checklist item and gives methodological background and published examples of transparent reporting. The STROBE checklist is best used in conjunction with this article (freely available on the Web sites of PLoS Medicine at http://www.plosmedicine.org/, Annals of Internal Medicine at http://www.annals.org/, and Epidemiology at http://www.epidem.com/). Information on the STROBE Initiative is available at www.strobe-statement.org.

For peer review only

BMJ Open

An evaluation of prescribing trends and patterns of claims within the Preferred Drugs Initiative in Ireland (2011-2016): an interrupted time-series study

Journal:	*BMJ Open*
Manuscript ID	bmjopen-2017-019315.R3
Article Type:	Research
Date Submitted by the Author:	17-Jan-2018
Complete List of Authors:	McDowell, Ronald; Royal College of Surgeons in Ireland, HRB Centre for Primary Care Research<br>Bennett, Kathleen; Royal College of Surgeons in Ireland, Population Health Sciences<br>Moriarty, Frank; Royal College of Surgeons in Ireland, HRB Centre for Primary Care Research, Department of General Practice<br>Clarke, Sarah; Health Service Executive Medicines Management Programme<br>Barry, Michael; National Centre for Pharmacoeconomics, Trinity Centre for Health Sciences<br>Fahey, Tom; Royal College of Surgeons in Ireland, Department of General Practice
**Primary Subject Heading**:	Epidemiology
Secondary Subject Heading:	General practice / Family practice, Health policy, Public health
Keywords:	Ireland, physician prescribing patterns, interrupted time series

SCHOLARONE™
Manuscripts

An evaluation of prescribing trends and patterns of claims within the Preferred Drugs Initiative in Ireland (2011-2016): an interrupted time-series study

McDowell RD[1], Bennett K[2], Moriarty F[1], Clarke S[3], Barry M[4], Fahey T[1]

[1] HRB Centre for Primary Care Research,
Department of General Practice,
Royal College of Surgeons in Ireland Medical School,
123 St Stephens Green,
Dublin 2

[2] Division of Population Health Sciences,
Royal College of Surgeons in Ireland,
Beaux Lane House,
Mercer Street Lower,
Dublin 2

[3] Health Service Executive Medicines Management Programme,
Trinity Centre for Health Sciences,
St. James's Hospital,
Dublin 8

[4] National Centre for Pharmacoeconomics,
Trinity Centre for Health Sciences,
St. James's Hospital,
Dublin 8

Correspondence to:

Ronald McDowell
Email: ronaldmcdowell@rcsi.ie

Word count: 4,175

Keywords: Ireland, physician prescribing patterns, interrupted time series

For peer review only

Abstract

Objective: To examine the impact of the Preferred Drugs Initiative (PDI), an Irish health policy aimed at enhancing evidence-based cost-effective prescribing, on prescribing trends and the cost of prescription medicines across seven medication classes.

Design: Retrospective repeated cross-sectional study spanning the years 2011 to 2016.

Setting: Health Service Executive Primary Care Reimbursement Scheme pharmacy claims data for General Medical Services (GMS) patients, approximately 40% of the Irish population.

Participants: Adults aged ≥18 years between 2011 to 2016 eligible for the GMS scheme.

Primary and secondary outcomes: The percentage of PDI medications within each drug class per calendar quarter. Linear regression was used to model prescribing of the preferred drug within each medication group and to assess the impact of PDI guidelines and other relevant changes in prescribing practice. Savings in drug expenditure were estimated.

Results: Between 2011 and 2016 around one quarter (23.59%) of all medications were for single-agent drugs licensed in the seven drug classes. There was a small increase in the percentage of PDI drugs, increasing from 4.64% of all medications in 2011 to 4.76% in 2016 ($p<0.001$). The percentage of preferred drugs within each drug class was significantly higher immediately following publication of the guidelines for all classes except urology, with the largest increases noted for lansoprazole (1.21%, 95%CI: 0.84% to 1.57%, $p<0.001$) and venlafaxine (0.71%, 95%CI: 0.15% to 1.27%), $p=0.02$). Trends in prescribing of the preferred drugs between PDI guidelines and the end of 2016 varied between drug classes. Total cost savings between 2013 and 2016 were estimated to be €2.7million.

Conclusion: There has been a small increase in prescribing of PDI drugs in response to prescribing guidelines, with inconsistent changes observed across therapeutic classes. These findings are relevant where health services are seeking to develop more active prescribing interventions aimed at changing prescribing practice.

For peer review only - http://bmjopen.bmj.com/site/about/guidelines.xhtml

For peer review only

Strengths and limitations of this study

- PCRS data covers pharmacy claims for prescriptions issued to General Medical Services (GMS) Scheme eligible patients (around 40% of the Irish population)
- Methods used are appropriate given the phased introduction of the preferred drug guidelines
- GMS patients over-represent older adults and those in receipt of social welfare
- Results based on aggregated data give an overview of the Preferred Drugs Initiative in its early years but require further detailed analysis to examine prescriber and patient heterogeneity.

For peer review only

For peer review only - http://bmjopen.bmj.com/site/about/guidelines.xhtml

Background

The Health Service Executive (HSE) in Ireland spent €1.05 billion in 2015 reimbursing pharmacists for the cost of prescription items issued to General Medical Services (GMS) eligible patients via the Primary Care Reimbursement Scheme (PCRS).(1) This is the largest community drug scheme in Ireland, providing access to free or minimal cost health care for patients whose household income falls below the eligibility threshold specified by the Irish Government, as well as the majority of people aged ≥70 years (approximately 95%) where a higher income threshold applies. Currently GMS eligible patients in Ireland have their prescription charges paid directly by the State, with a patient-levy of €2.50 for each item dispensed, up to a maximum of €25 per month. Historically Ireland has spent as much as 50% above the EU average per capita on drugs for a variety of reasons, such as low levels of use of generic medications and higher negotiated prices with pharmaceutical companies for both patented and generic drugs.(2, 3)

Against the background of an ageing population (4), the economic downturn of 2008 and rising drug costs the HSE established the Medicines Management Programme (MMP) in 2013. The MMP has undertaken a number of initiatives aimed at enhancing evidence-based and cost-effective prescribing (5), one of which is the Preferred Drugs Initiative (PDI). The PDI recommends a single 'preferred drug' within a therapeutic drug class as the prescriber's drug of first choice. Factors considered when selecting the preferred drug include clinical efficacy, ease of administration, the possibility of side effects or interactions with other drugs, cost, and national and international clinical guidelines. Recommendations for preferred drugs are made on an ongoing basis, with the findings disseminated through the publication of prescribing guidelines and GP meetings. The regulations covering generic substitution of branded medications are separate to the PDI guidelines, with generic substitution of drugs implemented where possible unless there are clinical reasons for prescribing the branded medication. The issuing of preferred drugs is voluntary and no incentives are given to prescribers to issue the preferred drug instead of others from within the same therapeutic drug class, with the patient levy remaining unaltered irrespective of preferred- or non-preferred drug status. Although the preferred drug may not necessarily be the least expensive licensed medication within each drug class, it has been estimated that increased provision of the preferred drugs could save the HSE €15 million per year.(5)

For peer review only - http://bmjopen.bmj.com/site/about/guidelines.xhtml

As of September 2016 reports detailing the rationale behind the choice of the preferred drugs have been published for the first ten therapeutic drug classes covered by the Initiative.(6) These are proton pump inhibitors (PPIs), statins, angiotensin-converting enzyme (ACE) inhibitors, angiotensin-II receptor blockers (ARBs), serotonin noradrenaline reuptake inhibitors (SNRIs), selective serotonin reuptake inhibitors (SSRIs), medications for treating urological conditions (urinary incontinence, frequency and overactive bladder), oral anticoagulants for stroke prevention in patients with non-valvular atrial fibrillation, beta-blockers and calcium channel blockers. There has been no evaluation of changes in prescribing following the introduction of the PDI to date. The aims of this paper are to: (i) examine the trends and patterns of pharmacy claims for seven PDI drug classes among eligible adult GMS patients in Ireland between 2011 and 2016; (ii) assess the impact of the PDI recommendations over time using segmented regression analysis; and (iii) estimate the cost savings due to the PDI during these years.

For peer review only - http://bmjopen.bmj.com/site/about/guidelines.xhtml

Methods

The STrengthening the Reporting of Observational Studies in Epidemiology (STROBE) guidelines were used in the reporting of this study (7).

Data

HSE-PCRS monthly pharmacy claims were analysed from 2011 to 2016 (8). This study period provided an average of three years of claims data both before and after the PDI across the seven drug classes considered. The data includes all pharmacy claims made for GMS patients and for which the cost of the claim has been reimbursed to community pharmacies by the HSE.

Preferred Drugs Initiative

The first seven medication classes covered by the PDI are considered in this paper. The preferred drugs in each of these classes were lansoprazole (PPIs), simvastatin (statins), ramipril (ACE inhibitors), candesartan (ARBs), venlafaxine (SNRIs), citalopram (SSRIs) and extended release (ER) tolterodine (urology medications). Guidelines for beta-blockers and calcium channel blockers were introduced in September 2016. Prescriptions issued to children (those under 18 years), hospital emergency items, out-of-hours prescriptions and items not considered medications (such as medical devices and dressings) were excluded; the PDI is primarily aimed at the treatment of adults in the general population.

Analytical methods/approach

Descriptive statistics were used to summarise relevant medications from the HSE-PCRS database and the classes of PDI drugs. Only single-agent drugs are considered in this paper, as this is the primary focus of the PDI.

The time-scale used for the analyses of time series depends on the research question of interest (9). Calendar quarters (January-March, April-June, July-September, October-December) were used to aggregate the data consistent with other analyses of prescribing data using interrupted time series (10-12). The use of calendar quarters was deemed clinically appropriate: changes in prescribing patterns tend to be gradual and guidelines are not necessarily disseminated or actioned on the first day of each calendar month. Furthermore Irish GMS eligible patients in receipt of prescription medication can receive three-months' worth of repeat prescriptions per consultation with their GP. For each therapeutic drug class a linear

For peer review only - http://bmjopen.bmj.com/site/about/guidelines.xhtml

regression model was used to estimate the percentage of the preferred drug per drug class per calendar quarter between 2011 to 2016, allowing for any changes that might have taken place following issuing of guidelines or other changes in clinical practice. This is a commonly-used strategy for analysing interrupted time series.(13) For medicine groups where the only “interruption” considered was dissemination of PDI guidelines, the regression equations used had the form

$$p_{ij} \quad = (\beta_{oj} + \beta_{1j}x_{ij1} + \beta_{2j}x_{ij2} + \beta_{3j}x_{ij3}) + e_{ij} \qquad (i = 0, \ldots, 23)$$

where for each medicine group j $(j = 1, \ldots, 7)$

p_{ij} is the percentage of items of the preferred drug reimbursed at time (quarter) i

β_{oj} is the estimated percentage of items being preferred drugs at t=0 (Jan-Mar 2011),

β_{1j} is the estimated change in the percentage of items being preferred drugs immediately following guidelines (the “change of level”)

β_{2j} is the estimated change in the percentage of items being preferred drugs per calendar quarter (the “slope”) before the guidelines

β_{3j} is the estimated change in the percentage of items being preferred drugs per calendar quarter (the “slope”) post -guidelines

e_{ij} is the residual for calendar quarter i.

The x_{ijk} $(k = 1,2,3)$ were calculated from the data according to standard practice. (14)

More than one change of level can be incorporated into any interrupted time series where this is relevant to the research question (13, 15). It was not feasible to include changes in the price of drugs in these models given the large number of drugs considered. Across the drug classes all drugs were licensed and available in Ireland between 2011 and 2016, and all generics were licensed prior to the study period, the key exceptions being the licensing of generic duloxetine in April 2015 and the licensing of mirabegron in January 2013. These two events were incorporated into the analyses for SNRIs and urology medications respectively.

Examination of the autocorrelation and partial autocorrelation coefficients showed that there was significant residual autocorrelation between adjacent calendar quarters (but not between non-adjacent quarters) in each drug group, and this was incorporated into the models using Prais–Winsten regression (16). The potential for seasonal autocorrelation was also considered:

For peer review only - http://bmjopen.bmj.com/site/about/guidelines.xhtml

in this context seasonal autocorrelation would mean that a given medication within a drug class is on average more or less likely to be prescribed than other drugs in the same class by virtue of the time of year. The PDI guidelines do not refer to any such clinical considerations (6) and we additionally hypothesised that seasonal autocorrelation would not be of statistical significance. This hypothesis was tested for each drug class by comparing the regression models which included Fourier terms to account for seasonality (9) and models without the seasonality terms. For each drug class seasonal autocorrelation was not of statistical significance and the seasonality terms were removed on the grounds of parsimony.

The PDI guidelines were national guidelines and consequently no control groups were available with which to compare prescribing under the PDI. However, we constructed two reference groups using the drug classes beta-blockers and calcium channel blockers. These were drug classes for which PDI guidelines were launched in September 2016 (the preferred drugs being bisoprolol and amlodipine respectively) but for which no recommendations had been made when the PDI guidelines were launched for the other drug classes. Given that the earlier guidelines were launched within six months of each other, two additional models were fitted: one examining prescribing of bisoprolol as the preferred beta-blocker over the study period, allowing for potential changes in prescribing when guidelines for PPIs/statins (April 2013) and SNRIs/SSRIs (April 2014) were disseminated, and one model examining prescribing of amlodipine as the preferred calcium channel blocker, allowing for potential changes in prescribing when guidelines for ACE inhibitors/ARBs (Sept 2013) and urology medications (October 2014) were issued.

By coincidence rather than design issuing of guidelines for each medicine group occurred at the beginning of the calendar quarters listed above, with the exception of the guidelines for ACE inhibitors and ARBs. Sensitivity analyses were used to explore whether the results varied when the calendar quarters were constructed differently (March-May, June-August, September-November, December-February) for these groups. Given that the PDI guidelines were launched in phases, sensitivity analyses were also used to examine whether results were dependent on the length of time considered before and after guidelines.

The models above were used to estimate increases or decreases in costs for each drug group associated with the PDI. Where only one interruption to the time series was included in the model, the predicted number of preferred drug items from each class was compared with the

For peer review only - http://bmjopen.bmj.com/site/about/guidelines.xhtml

number which would have been issued had the trend in prescribing estimated before the guidelines continued i.e. the estimates of $\beta_{oj}(\widehat{\beta_{oJ}})$ and $\beta_{2j}(\widehat{\beta_{2J}})$ remained unchanged, $\widehat{\beta_{1J}}$ was constrained to be zero and the estimate of $\beta_{3j}(\widehat{\beta_{3J}})$ was set equal to $\widehat{\beta_{2J}}$. The difference in the number of preferred drug items under the two scenarios was multiplied by the average price of the preferred drug, calculated across all reimbursements between dissemination of the guidelines and the end of 2016. The difference in the number of non-preferred drug items was multiplied by a weighted average of the price of all other drugs from within the medicine class, weighted according to the overall distribution of these items between issuing of the guidelines and the end of December 2016. These two costs were combined to give an overall cost differential. The process was extended analogously to include multiple interruptions as appropriate.

All analyses were conducted using Stata 14.0SE.(17) Results were held to be significant if they referred to statistical significance on a two-sided design-based test evaluated at the 0.05% level.

For peer review only

For peer review only - http://bmjopen.bmj.com/site/about/guidelines.xhtml

Results

Descriptive statistics

A total of 336,535,263 prescription items for medications were reimbursed by 4,465 PCRS prescribers for 1,919,681 GMS adults aged 18 years and over between 2011 and 2016. The median number of items reimbursed per GMS patient was 63 (Interquartile Range (IQR) 13 to 246) with a median total cost per patient of €905.75 (IQR €170.25 to €9,726.93). Approximately 55 million items were reimbursed per year, with the number of items peaking slightly in 2012 and 2013. During the six-year period 48.8 million (19.86%) prescription items were for the single-agent medicines licensed across the seven therapeutic drug classes considered. The drug classes most commonly prescribed to GMS patients were statins (5.93% of all items) and PPIs (5.63%), with the least common being SNRIs (0.99%) and drugs for treating urological conditions (0.67%). The descriptive statistics for each PDI medication class over the six-year period are outlined in Table 1.

The percentage of items relating to the seven drug classes increased slightly from 19.57% in 2011 to 20.04% in 2016, with small changes observed in the volume of prescriptions issued per each PDI medicine group over this time. More detailed breakdowns of PDI medicine groups per calendar year and quarter are given in Appendix Tables A1 & A2 and Figure A1.

Preferred Drugs Initiative

Within the seven PDI drug classes considered, 23.59% of all prescription items were for the named preferred drugs. However, there was considerable variation between PDI drug classes both in terms of ranking and percentage coverage of the preferred drug (see Table 1). The most commonly prescribed preferred drug within the relevant drug class was venlafaxine, which comprised 70.99% of all SNRI prescriptions. This was followed by ramipril (53.41% of all single-agent ACE inhibitors), ER tolterodine (25.79% of urology items), lansoprazole (24.14% of PPIs), citalopram (19.77% of SSRIs), candesartan (10.78% of all single-agent ARBs) and simvastatin (6.59% of all single-agent statins). The ranking of the preferred drugs within classes varied from first (ACE inhibitors and SNRIs), to second-last (statins). There was a small but statistically significant increase over time in the percentage of all medications which were for the PDI drugs, increasing from 4.64% in 2011 to 4.76% in 2016 ($p<0.001$).

Impact of clinical guidelines

For peer review only - http://bmjopen.bmj.com/site/about/guidelines.xhtml

Comparing prescribing patterns within each medication class in the three months pre-and post-publication of the PDI guidelines there was a small increase in the proportion of preferred drugs in four drug classes (PPIs (p<0.001), statins (p<0.001), ACE inhibitors (p<0.001) and SNRIs (p=0.08)), little change in two other drugs classes (ARBs (p=0.76) and SSRIs (p=0.37)), and a reduction in percentage terms in prescribing of the PDI agent ER tolterodine (p<0.001) (Table 1). Two preferred drugs, citalopram and ER tolterodine, were ranked lower within their respective classes between issuing of the guidelines and the end of 2016 than before. Figure 1 illustrates the secular trends for preferred drugs across the PDI categories by calendar quarters between 2011 and 2016: plots of the actual percentage of preferred drug items within each drug group between 2011 and 2016 are given in Appendix Figure A2.

Segmented linear regression showed changes over time in the prescribing of all preferred drugs (Table 2). In all medicine groups except urology, there was evidence of significant increases in prescribing of the preferred drugs immediately following dissemination of the PDI guidelines . For three medicine groups, there was significant evidence of an increase in the percentage of preferred drug items in the quarter immediately following issuing of the guidelines (lansoprazole (1.21%, 95%CI: 0.84% to 1.57%, p<0.001); venlafaxine (0.71%, 95%CI: 0.15% to 1.27%, p<0.001); simvastatin (0.30%, 95%CI: 0.1% to 0.5%, p=0.01)) and small increases in prescribing of the preferred drug in subsequent quarters. The percentage of SNRI medications which were venlafaxine did not change significantly immediately following the licensing of generic duloxetine in April 2015 (p=0.76) or in subsequent quarters (p=0.34). For both candesartan and citalopram, for which prescribing within their PDI drug classes was in decline prior to the guidelines being issued, prescribing increased immediately following the PDI guidelines (candesartan (0.15%, 95%CI: 0.02 to 0.29, p=0.03); citalopram (OR 0.30%, 95%CI: 0.12% to 0.47%, p=0.002)) but did not continue to increase significantly in subsequent quarters. Indeed declines in prescribing of citalopram resumed in July 2014, although the decline was less steep than before the guidelines (p<0.001). There was a small increase in prescribing of the preferred ACE inhibitor (ramipril) immediately following the PDI guidelines (0.16%, 9%CI: 0.01 to 0.31, p=0.04), although subsequent increases per calendar quarter did not differ significantly at the 5% level from increases observed per calendar quarter prior to the PDI guidelines (p=0.08). No statistically significant changes were observed in the prescribing of ER tolterodine immediately following the licensing of mirabegron in January 2013 (p=0.52) or the PDI guidelines in October 2014 (p=0.82), although the rate of decline in prescribing of ER

For peer review only - http://bmjopen.bmj.com/site/about/guidelines.xhtml

tolterodine was lower following the PDI guidelines than between the licensing of mirabegron and dissemination of the PDI guidelines (p<0.001).

Sensitivity analyses showed that the results were materially unaffected when the calendar quarters used for analyses of ACE inhibitors and ARBs varied or when the length of time studied before and after the guidelines was changed (Appendix Tables A3, A4).

Reference groups

Beta-blockers and calcium channel blockers accounted for 3.58% (n=12,056,378) and 2.30% (n=7,753,755) of single-agent medications for GMS patients between 2011 and 2016, with the most commonly prescribed medications being bisoprolol (56.83% of all single-agent beta-blockers (n=6,852,022)) and amlodipine (64.70% of all single-agent calcium channel blockers (n=5,016,348)), both of which were selected as preferred drugs in September 2016. There was a steady increase in prescribing of bisoprolol as the beta-blocker of choice and a consistent fall in prescribing of amlodipine within the calcium channel blocker medications over the study period. Effects in these drug groups associated with dissemination of the PDI guidelines for the other drug groups were non-significant at the 5% level (Table 3). See Figure 2 for plots of the estimated percentage of preferred drug items within each therapeutic drug class between 2011 and 2016.

Cost savings

Overall, the cost savings after introduction of the PDI amounted to €2,671k across all seven PDI drug classes (Table 2). The savings associated with changes in prescribing following issuing of guidelines for the seven drug classes were estimated to be €123k in 2013, €396k in 2014, €837k in 2015 and €1,314k in 2016. There were savings in each group, even though changes in dispensed medications were often minimal. The greatest impact was on the amount spent on SNRIs, with an estimated saving of €1,291k between 2014-2016. This is due to the much higher cost of the non-preferred drug duloxetine to the preferred drug venlafaxine. Other groups where the savings were marked were for the two larger volume groups where the guidelines had first been issued- PPIs saving €618k and statins saving €363k. For medicine groups where prescribing of the preferred drug was in decline before guidelines were issued, even the small short-term changes in prescribing translated into some savings. The smallest cost savings were

For peer review only - http://bmjopen.bmj.com/site/about/guidelines.xhtml

in the prescribing of ramipril and ER tolterodine, due to the lack of change in prescribing trends observed within these groups between 2011 and 2016. The combined savings in the reference groups, had the prescribing patterns observed prior to the PDI guidelines remained unchanged, was an estimated €17k.

For peer review only

Discussion

Principal findings

The seven drug classes considered that form part of the PDI accounted for approximately 20% of all medications reimbursed by the PCRS between 2011 and 2016. Changes in prescribing observed over the study period varied by PDI drug class, with substantial differences in the ranking order and quantity of preferred drug prescribed. Overall, the impact of the PDI guidance was limited, with an inconsistent pattern observed across all therapeutic drug classes, and only a small increase (0.13%) in the percentage of preferred drugs issued overall between 2011 and 2016. Across the PDI drug classes some differences emerged: in the first group of PDI drugs there were increases in prescribing of the preferred drug immediately following issuing of the guidelines and continued though small increases subsequently (PPIs, Statins and SNRIs); in the second group of PDI drugs (SSRIs and ARBs) there was a temporary increase in prescribing of the preferred drug just after the guidelines were issued; lastly, in the third group of PDI drugs (ACE and urology), there appeared to be little or no impact of clinical guidance. The reasons for such diversity are not known. ACE inhibitors are relatively inexpensive and this may account, in part, for the trend in ramipril prescribing remaining relatively unchanged. Although mirabegron has become the most commonly prescribed urology item since its launch in 2013, prescribing of ER Tolterodine was in decline prior to this time.

Context of other studies

PDI guidelines to date have been disseminated to prescribers mainly through correspondence and GP meetings. The literature shows that educational programmes and publication of guidelines in themselves tend to have little effect on influencing prescribing practice, and that these need to be enhanced with other strategies.(18) In a systematic review of 79 studies examining interventions which changed doctor prescribing behaviour, the most effective interventions were patient-mediated interventions, outreach, audit and feedback, and reminders.(19) In a study of changes in the use of losartan versus other single ARBs in Sweden investigators concluded that multiple and intensive demand-side measures are needed to change physician prescribing habits.(20) Other strategies which have been found to be helpful include direct involvement of the community pharmacist and face-to-face engagement from those seeking to encourage change with the prescriber .(21) Technological advances, such as alerts and prompts when issuing a drug may also prove useful. (22)

For peer review only - http://bmjopen.bmj.com/site/about/guidelines.xhtml

Any excess expenditure incurred through the issuing of non-preferred drugs to GMS patients is met directly by the HSE and not by the patient. Options which could reduce such expenditure include reducing choice for either patient or prescriber. It has been suggested that because prescribers can develop expertise of only a certain number of drugs, more restrictive formularies may also provide benefits to quality of prescribing (23, 24). In Sweden, the introduction of the 'Wise List', an evidence-based formulary of essential medicines, increased adherence to guideline recommendations in primary care from 80% to 90% and reduced variation in prescribing (25). The introduction of co-payments, where the patient has to pay the difference between the price of the preferred drug and their chosen alternative, has the potential to be a considerable driver of change. Australia operates a therapeutic brand premium scheme, whereby a co-payment is required from patients when a prescriber has issued a drug within a drug class that is priced above the benchmark for drugs in that group.(26) While dramatic changes in co-payments may result in more patients switching to preferred agents (such as statins, ACE inhibitors and PPIs), they may also increase the risk of patients stopping their medication or becoming non-adherent (27, 28). Recent work has shown the drivers of drug expenditure in high income countries varies substantially, with several other factors aside from physician prescribing behavior and patient preference determining national drug expenditure.(29)

Strengths and limitations

There are a number of strengths to this study. Our prescription sample is large and generalisable: PCRS data covers the entire GMS population of Ireland (around 40% of individuals). Despite the guidelines being introduced incrementally, the results were invariant to the time periods studied pre- and post-publication of clinical guidelines. However, there are limitations to the study. GMS patients are weighted towards older adults and those socially and financially disadvantaged and so the results may not be reflective of the entire population in receipt of prescription medication. There is no way of knowing whether prescribers approached patients with regards to changes in their medication and/or whether these approaches were successful. Patient-specific factors may mean that issuing of the preferred drug may not have been appropriate or possible. Neither prescribers nor patients are homogeneous entities and considerable variation may exist within both. Although the changes in prescribing observed within the PDI medicine groups were not observed in the reference groups, there may be factors

For peer review only - http://bmjopen.bmj.com/site/about/guidelines.xhtml

other than the PDI guidelines which have contributed to prescribing changes and the associated cost savings within the PDI drug groups.

Policy implications and future research

The PDI has been developed to encourage evidence-based, cost-effective prescribing, but in view of the limited changes to date has delivered only a small amount of cost savings in terms of the money spent on these prescription items. If cost savings are to be maximised, the energies need to focus on medicine groups which are large volume (e.g. PPIs and statins) and/or where there is considerable variation between the least and most expensive licensed medications in that group (e.g. SNRIs). To enhance the impact of the PDI, multi-faceted interventions appear most likely to succeed. Financial incentives to prescribers may be one possible component of such interventions, as operated in Irish primary care for a time in the 1990's (30), however any incentives for PDI drugs need to be aligned with professional values of prescriber, and be mindful of personal preferences of patients taking long-term medication (31-33). The effectiveness of such interventions is important to consider and although this has generally been evaluated using observational methods, experimental approaches may also be feasible.

Given the increasing demand for and costs associated with health-care provision world-wide, findings from this evaluation may be of interest to other countries seeking to provide treatment that is both evidence-based and cost-effective. This includes countries already implementing preferred drug schemes (e.g. Australia), those which are considering such schemes or indeed any intervention aimed at changing clinical practice. The results show that initiatives which are primarily voluntary in nature may be impactful but their impact can be limited and short-term. They also show that interventions launched concurrently and developed using the same methodological framework may not necessarily yield similar results.

Conclusions

Since the introduction of the PDI in 2013, there have been some cost savings across the PDI drug classes. However, more intensive implementation is needed if the PDI is to deliver the estimated €15million per year cost saving that was anticipated. Multifaceted interventions will be required to enhance the coverage and impact of the PDI so that these benefits can be realised.

For peer review only - http://bmjopen.bmj.com/site/about/guidelines.xhtml

Acknowledgements: Thanks to the Health Services Executive Primary Care Reimbursement Scheme (HSE-PCRS) for the use of pharmacy claims database.

Contributors: RMcD drafted and planned all aspects of study design, cleaned and prepared data for analysis, conducted the statistical analyses and conducted a preliminary overview of the literature. KB prepared the monthly PCRS claims downloads and gave significant methodological guidance on the analysis strategy. FM provided guidance on pharmaceutical matters and contributed to the discussion on context, policy implications and future research. SC and MB facilitated access to the claims data with the PCRS, gave detailed information on roll-out and implementation of the Preferred Drugs Initiative, and contributed to interpretation of the results within the wider context of prescribing in Ireland. TF generated the research question and commented on the conduct, analysis and write-up of the paper. All authors read and approved submission of the paper.

Funding: The primary author was funded by the Health Research Board (HRB) of Ireland under the HRB Centre for Primary Care Phase 2 Funding award, grant HRC/2014/1. KB funded by HRB under grant RL-15-1579

Competing interests: None declared.

Data-sharing statement: No additional data are available.

For peer review only

For peer review only - http://bmjopen.bmj.com/site/about/guidelines.xhtml

References

1. Service HSEPCR. Primary Care reimbursement Service Statistical Analysis of Claims and Payments 2015. Available from: http://www.hse.ie/eng/staff/PCRS/PCRS_Publications/PCRS-statistical-analysis-of-claims-and-payments-2015.pdf.
2. Children HotOJCoHa. Report on the Cost of Prescription Drugs in Ireland 2015. 2015.
3. Brick A GP, Nolan A. Ireland: Pharmaceutical Prices, Prescribing Practices and Usage of Generics in a Comparative Context. Dublin: ESRI, 2013.
4. Layte R. (ed) BM, Bennett K, Brick A, Morgenroth E., Normand C, O'Reilly J, Thomas S, Tilson L, Wiley M, Wren M.-A. Demographic Change on the Demand for and Delivery of Health Care in Ireland. Dublin: ESRI, 2009.
5. Programme MM. Welcome to the Medicines Management Programme 2016. Available from: http://www.hse.ie/eng/about/Who/clinical/natclinprog/medicinemanagementprogramme/yourmedicines/.
6. Programme MM. Evaluation Reports. 2017.
7. von Elm E, Altman DG, Egger M, Pocock SJ, Gotzsche PC, Vandenbroucke JP, et al. The Strengthening the Reporting of Observational Studies in Epidemiology (STROBE) statement: guidelines for reporting observational studies. Journal of clinical epidemiology. 2008;61(4):344-9.
8. Sinnott SJ, Bennett K, Cahir C. Pharmacoepidemiology resources in Ireland-an introduction to pharmacy claims data. European journal of clinical pharmacology. 2017.
9. Bhaskaran K, Gasparrini A, Hajat S, Smeeth L, Armstrong B. Time series regression studies in environmental epidemiology. International journal of epidemiology. 2013;42(4):1187-95.
10. Rector TS, Finch MD, Danzon PM, Pauly MV, Manda BS. Effect of tiered prescription copayments on the use of preferred brand medications. Medical care. 2003;41(3):398-406.
11. Lopatto J, Keith SW, Del Canale S, Templin M, Maio V. Evaluating sustained quality improvements: long-term effectiveness of a physician-focused intervention to reduce potentially inappropriate medication prescribing in an older population. Journal of Clinical Pharmacy & Therapeutics. 2014;39(3):266-71.
12. Penfold RB, Zhang F. Use of interrupted time series analysis in evaluating health care quality improvements. Academic pediatrics. 2013;13(6 Suppl):S38-44.
13. Wagner AK, Soumerai SB, Zhang F, Ross-Degnan D. Segmented regression analysis of interrupted time series studies in medication use research. Journal of clinical pharmacy and therapeutics. 2002;27(4):299-309.
14. Education UIfDRa. How can I run a piecewise regression in Stata? 2017. Available from: https://stats.idre.ucla.edu/stata/faq/how-can-i-run-a-piecewise-regression-in-stata/.
15. Bernal J, Cummins, S, Gasparrini, A. Interrupted time series regression for the evaluation of public health interventions: a tutorial. International journal of epidemiology. 2017;46(1):384-55.
16. Prais SJ, and C. B. Winsten. Trend estimators and serial correlation: Working paper 383, Cowles Commission; 1954. Available from: http://cowles.econ.yale.edu/P/ccdp/st/s-0383.pdf.
17. StataCorp. Stata Statistical Software. Texas: StataCorp; 2015.
18. Davis D, O'Brien MA, Freemantle N, Wolf FM, Mazmanian P, Taylor-Vaisey A. Impact of formal continuing medical education: do conferences, workshops, rounds, and other traditional continuing education activities change physician behavior or health care outcomes? Jama. 1999;282(9):867-74.
19. Gill PS, Makela M, Vermeulen KM, Freemantle N, Ryan G, Bond C, et al. Changing doctor prescribing behaviour. Pharmacy world & science : PWS. 1999;21(4):158-67.
20. Godman B, Wettermark B, Miranda J, Bennie M, Martin A, Malmstrom RE. Influence of multiple initiatives in Sweden to enhance ARB prescribing efficiency following generic losartan; findings and implications for other countries. International journal of clinical practice. 2013;67(9):853-62.

For peer review only - http://bmjopen.bmj.com/site/about/guidelines.xhtml

21. Soumerai SB, Avorn J. Principles of educational outreach ('academic detailing') to improve clinical decision making. Jama. 1990;263(4):549-56.
22. Kuperman GJ, Bobb A, Payne TH, Avery AJ, Gandhi TK, Burns G, et al. Medication-related clinical decision support in computerized provider order entry systems: a review. Journal of the American Medical Informatics Association : JAMIA. 2007;14(1):29-40.
23. Persaud N, Ahmad H. Canadian list of essential medications: Potential and uncertainties. Canadian family physician Medecin de famille canadien. 2017;63(4):266-8.
24. Wettermark B, Pehrsson A, Jinnerot D, Bergman U. Drug utilisation 90% profiles--a useful tool for quality assessment of prescribing in primary health care in Stockholm. Pharmacoepidemiology and drug safety. 2003;12(6):499-510.
25. Eriksen J, Gustafsson LL, Ateva K, Bastholm-Rahmner P, Ovesjo ML, Jirlow M, et al. High adherence to the 'Wise List' treatment recommendations in Stockholm: a 15-year retrospective review of a multifaceted approach promoting rational use of medicines. BMJ open. 2017;7(4):e014345.
26. Health AGo. Therapeutic Group Premium Policy 2017. Available from: https://www.pbs.gov.au/browse/group-premium.
27. Sinnott SJ, Normand C, Byrne S, Woods N, Whelton H. Copayments for prescription medicines on a public health insurance scheme in Ireland. Pharmacoepidemiology and drug safety. 2016;25(6):695-704.
28. Huskamp HA, Deverka PA, Epstein AM, Epstein RS, McGuigan KA, Frank RG. The effect of incentive-based formularies on prescription-drug utilization and spending. The New England journal of medicine. 2003;349(23):2224-32.
29. Morgan SG, Leopold C, Wagner AK. Drivers of expenditure on primary care prescription drugs in 10 high-income countries with universal health coverage. CMAJ : Canadian Medical Association journal = journal de l'Association medicale canadienne. 2017;189(23):E794-E9.
30. Walley T, Murphy M, Codd M, Johnston Z, Quirke T. Effects of a monetary incentive on primary care prescribing in Ireland: changes in prescribing patterns in one health board 1990-1995. Pharmacoepidemiology and drug safety. 2000;9(7):591-8.
31. Roland M. Incentives must be closely aligned to professional values. Bmj. 2012;345:e5982.
32. Majumdar SR, Soumerai SB. Why most interventions to improve physician prescribing do not seem to work. CMAJ : Canadian Medical Association journal = journal de l'Association medicale canadienne. 2003;169(1):30-1.
33. Persaud N, Lee T, Ahmad H, Li W, Taglione MS, Rajakulasingam Y, et al. Protocol for a randomised controlled trial evaluating the effects of providing essential medicines at no charge: the Carefully seLected and Easily Accessible at No Charge Medicines (CLEAN Meds) trial. BMJ open. 2017;7(5):e015686.

For peer review only - http://bmjopen.bmj.com/site/about/guidelines.xhtml

Table 1:Summary of impact of Preferred Drugs Initiative (2011-2016)

Preferred drug class	PPI	Statin	ACE	ARB	SNRI	SSRI	urology	Total
Total no. items	18,939,282	19,944,634	8,837,006	5,171,204	3,345,307	8,348,567	2,239,263	336,535,263
% of all drugs	5.63%	5.93%	2.63%	1.54%	0.99%	2.48%	0.67%	19.86%
**Preferred drug**	**lansoprazole**	**simvastatin**	**ramipril**	**candesartan**	**venlafaxine**	**citalopram**	**ER tolterodine**	
**Total no. single-agent items**	4,571,751	1,313,389	4,719,996	557,622	1,155,600	1,650,520	577,540	
**% within class**	24.14%	6.59%	53.41%	10.78%	70.99%	19.77%	25.79%	
**Rank within class pre-PDI**	2/5	4/5	1/10	5/8	1/2	2/6	1/9	
**Rank within class post-PDI**	2/5	4/5	1/10	5/8	1/2	3/6	3/9	
**Absolute change in proportion of preferred drug items: first 3 months post-PDI v previous 3 months**	↑ +0.98% (p<0.001)	↑ +0.30% (p<0.001)	↑ 0.53% (p<0.001)	↓ -0.03% (p=0.76)	↑ 0.30% (p=0.08)	↓ -0.09% (p=0.37)	↓ -0.98% (p<0.001)	

PPI: proton pump inhibitor; ACE: angiotensin-converting enzyme (ACE) inhibitor; ARB: angiotensin-II receptor blocker; SNRI: serotonin and noradrenaline reuptake inhibitor ; SSRI: selective serotonin receptor antagonist; ER: Extended Release; PDI: Preferred Drugs Initiative

For peer review only - http://bmjopen.bmj.com/site/about/guidelines.xhtml

Table 2: Segmented regression analysis in relation to PDI guideline publication, class-specific changes and cost savings

Medicine group (Preferred drug)	Guidelines introduced	Percentage of preferred drug items: Jan-March 2011 (SE), 95% CI	Increase in % of preferred drug items per quarter post March 2011 (SE), 95%CI, p-value	Increase in % of preferred drug items Jan-Mar 2013 following licensing of mirabegron, (SE), 95%CI, p-value	Increase in % of preferred drug items per quarter post March 2013 (SE), 95%CI, p-value	Increase in % of preferred drug items quarter immediately following PDI guidelines, (SE), 95%CI, p-value	Increase in % of preferred drug items per quarter post PDI guidelines, (SE), 95%CI, p-value	Increase in % of preferred drug April-June 2015 following introduction of generic duloxetine, (SE), 95%CI, p-value	Increase in % of preferred drug items per quarter post June 2015 , (SE), 95%CI, p-value	Estimated savings between issuing of guidelines and Dec 2016 (€)
PPIs (lansoprazole)	April 2013	24.53 (0.47), (23.59,25.47)	-0.21 (0.05), (-0.32,-0.11), p=0.001	-	-	1.21 (0.18), (0.84,1.57), p<0.001	0.04 (0.04) (-0.03,0.12), p=0.25	-	-	618,158
Statins (simvastatin)	April 2013	5.94 (0.21), (5.50,6.38)	0.02 (0.03), (-0.04,0.07), p=0.54	-	-	0.30 (0.10), (0.10,0.50), p=0.01	0.07 (0.02), (0.03,0.10), p=0.002	-	-	363,194
ACEs (ramipril)	September 2013	49.14 (0.07), (48.99,49.28)	0.38 (0.01). (0.35,0.40), p<0.001	-	-	0.16 (0.07), (0.01,0.31), p=0.04	0.41 (0.01), (0.39,0.42), p<0.001	-	-	50,163
ARBs (candesartan)	September 2013	11.90 (0.08), (11.73,12.07)	-0.15 (0.01), (-0.17,-0.12), p<0.001	-	-	0.15 (0.06), (0.02,0.29), p=0.03	0.01 (0.01), (-0.01,0.03), p=0.46	-	-	132,625
SNRIs (venlafaxine)	April 2014	73.61 (0.44), (72.69,74.53)	-0.35 (0.05), (-0.46,-0.24), p<0.001	-	-	0.71 (0.27), (0.15,1.27), p=0.02	0.26 (0.13), (-0.02,0.55), p=0.07	-0.09 (0.30), (-0.73,0.54), p=0.76	-0.08 (0.09), (-0.10,0.26), p=0.34	1,291,160
SSRIs (citalopram)	April 2014	23.58 (0.13), (23.31,23.85)	-0.36 (0.01), (-0.39,-0.33), p<0.001	-	-	0.30 (0.08), (0.12,0.47), p=0.002	-0.23 (0.02), (-0.27,-0.19), p<0.001	-	-	169,493
urology (ER tolterodine)	October 2014	37.27 (0.27), (36.69,37.84)	-1.00 (0.05), (-1.11,-0.88), p<0.001	0.16 (0.24), (-0.35,0.66), p=0.52	-1.04 (0.06), (-1.17,-0.91), p<0.001	-0.06 (0.24), (-0.57,0.45), p=0.82	-0.63 (0.09), (-0.73,-0.52), p<0.001	-	-	46,695
**Total savings**										**2,671,447**

For peer review only - http://bmjopen.bmj.com/site/about/guidelines.xhtml

CI: Confidence Interval; SE: Standard Error; ER: Extended Release; PPI: proton pump inhibitor; ACE: angiotensin-converting enzyme (ACE) inhibitor; ARB: angiotensin-II receptor blocker; SNRI: serotonin and noradrenaline reuptake inhibitor ; SSRI: selective serotonin receptor antagonist; ER: Extended Release; PDI: Preferred Drugs Initiative

For peer review only

For peer review only - http://bmjopen.bmj.com/site/about/guidelines.xhtml

Table 3: Segmented regression analysis in relation to PDI guideline publication, reference groups

**Medicine group (Preferred drug)**	**Guidelines introduced**	**Percentage of preferred drug items: Jan-March 2011 (SE), 95% CI**	**Increase in % of preferred drug items per quarter post March 2011 (SE), 95%CI, p-value**	**Increase in % of preferred drug items April-June 2013†, (SE), 95%CI, p-value**	**Increase in % of preferred drug items per quarter post June 2013, (SE), 95%CI, p-value**	**Increase in % of preferred drug items Oct-Dec 2013††, (SE), 95%CI, p-value**	**Increase in % of preferred drug items per quarter post Dec 2013, (SE), 95%CI, p-value**	**Increase in % of preferred drug items April-June 2014*, (SE), 95%CI, p-value**	**Increase in % of preferred drug items per quarter post June 2014, (SE), 95%CI, p-value**	**Increase in % of preferred drug items Oct-Dec 2014** (SE), 95%CI, p-value**	**Increase in % of preferred drug items per quarter post Dec 2014, (SE), 95%CI, p-value**
beta-blockers (bisoprolol)	September 2016	51.20 (0.03), (51.15,51.26)	0.53 (0.01), (0.52,0.54), p<0.001	-0.02 (0.05), (-0.13,0.09), p=0.71	0.50 (0.02), (0.45,0.54), p<0.001	-	-	-0.05 (0.06), (-0.18,0.08), p=0.44	0.41 (0.001), (0.40,0.42), p<0.001	-	-
calcium channel blockers (amlodipine)	September 2016	68.18 (0.03), (68.12,68.29)	-0.34 (0.01), (-0.35,-0.33), p<0.001	-	-	0.12 (0.06), (-0.001,0.23), p=0.06	-0.26 (0.02) (-0.31,-0.21), p<0.001	-	-	0.02 (0.07), (-0.13,0.17), p=0.76	-0.21 (0.01) (-0.22,-0.19), p<0.001

† introduction of PDI guidelines for PPIs/statins; †† introduction of PDI guidelines for ACE/ARBs; * introduction of PDI guidelines for SNRIs/SSRIs; ** introduction of PDI guidelines for urology medications; CI: Confidence Interval; SE: Standard Error; ER: Extended Release; PDI: Preferred Drugs Initiative

For peer review only - http://bmjopen.bmj.com/site/about/guidelines.xhtml

Figure Legends

Figure 1: Distribution of preferred drug items by therapeutic drug class

Figure 2: Estimated percentage of preferred drugs by drug class: segmented regression models

For peer review only

For peer review only - http://bmjopen.bmj.com/site/about/guidelines.xhtml

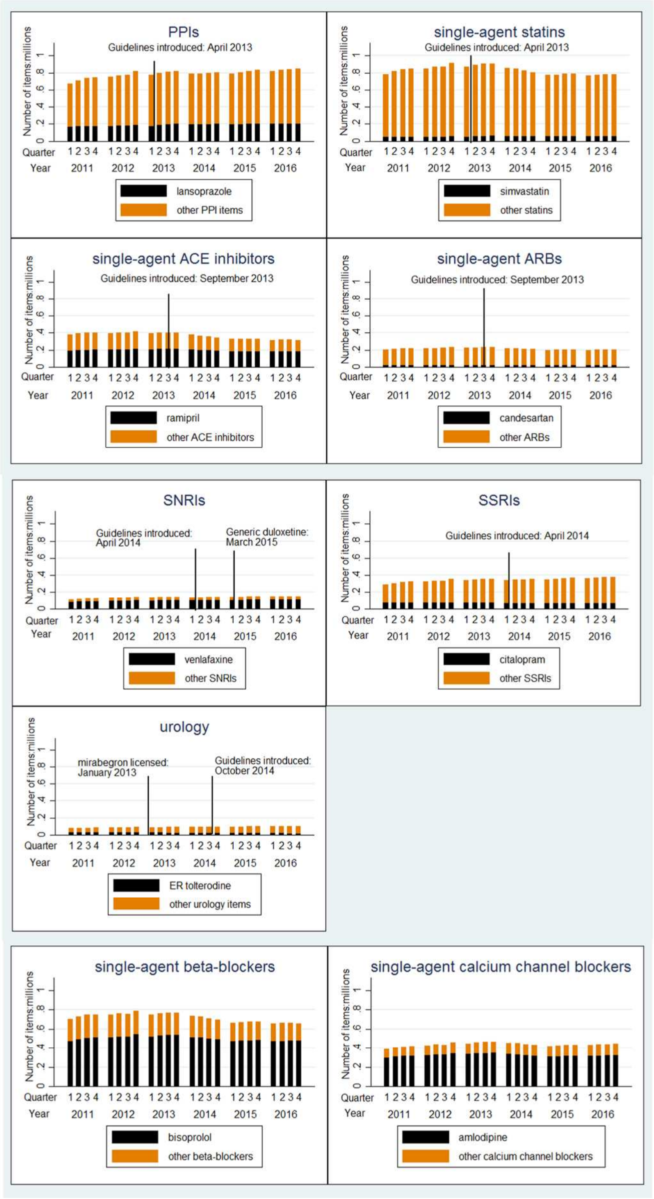

Figure 1: Distribution of preferred drug items by therapeutic drug class

159x290mm (300 x 300 DPI)

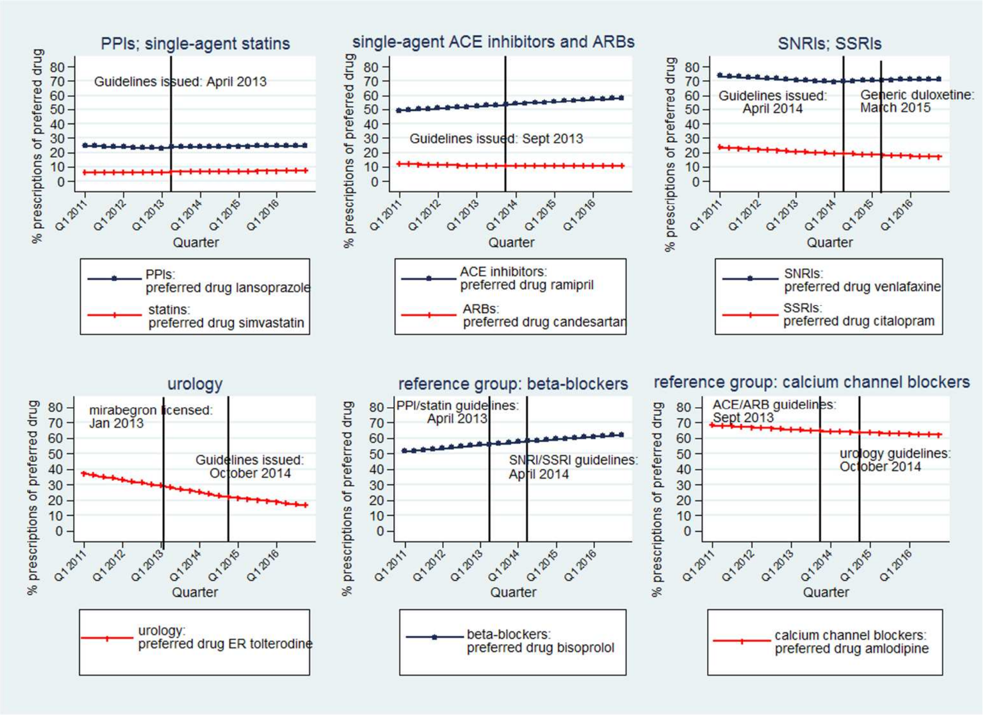

Figure 2: Estimated percentage of preferred drugs by drug class: segmented regression models

180x129mm (300 x 300 DPI)

Appendix

Table A1: Breakdown of PCRS reimbursed items: 2011-2016

Year	No. items issued	No. single-agent items issued across 7 drug classes*	% of items attributed to 7 drug classes*	No. items issued for preferred drugs	% preferred drug items within preferred drug classes	% preferred drug items across all prescriptions
**2011**	54,324,492	10,630,476	19.57%	2,520,986	23.71%	4.64%
**2012**	57,984,934	11,380,582	19.63%	2,641,897	23.21%	4.56%
**2013**	58,455,927	11,640,615	19.91%	2,708,855	23.27%	4.63%
**2014**	55,978,157	11,181,081	19.97%	2,655,422	23.75%	4.74%
**2015**	54,573,162	10,925,162	20.02%	2,610,926	23.90%	4.78%
**2016**	55,218,591	11,067,347	20.04%	2,627,631	23.74%	4.76%
**Total**	336,553,263	66,825,263	19.86%	15,765,717	23.59%	4.68%

***PPIs, Statins, ACEs, ARBs, SNRIs, SSRIs, Urology**

Table A2 Prevalence of PCRS reimbursed items by therapeutic drug class (single agent drugs)

Year	PPIs	statins	ACEs	ARBs	SNRIs	SSRIs	urology	Other	Total
**2011**	2,860,986 (5.27%)	3,286,352 (6.05%)	1,586,992 (2.92%)	849,807 (1.56%)	470,234 (0.87%)	1,247,643 (2.30%)	328,462 (0.60%)	43,694,016 (80.43%)	54,324,492 (100%)
**2012**	3,114,214 (5.37%)	3,501,257 (6.04%)	1,616,612 (2.79%)	899,594 (1.55%)	537,800 (0.93%)	1,355,921 (2.34%)	355,184 (0.61%)	46,604,352 (80.37%)	57,984,934 (100%)
**2013**	3,203,104 (5.48%)	3,582,112 (6.13%)	1,595,582 (2.73%)	920,851 (1.58%)	566,951 (0.97%)	1,404,466 (2.40%)	367,549 (0.63%)	46,815,312 (80.09%)	58,455,927 (100%)
**2014**	3,180,702 (5.68%)	3,339,227 (5.97%)	1,449,173 (2.59%)	867,567 (1.55%)	567,859 (1.01%)	1,399,724 (2.50%)	376,829 (0.67%)	44,797,076 (80.03%)	55,978,157 (100%)
**2015**	3,241,661 (5.94%)	3,129,117 (5.73%)	1,312,155 (2.40%)	816,250 (1.50%)	588,689 (1.08%)	1,441,270 (2.64%)	396,020 (0.73%)	43,648,000 (79.98%)	54,573,162 (100%)
**2016**	3,338,615 (6.05%)	3,106,569 (5.63%)	1,276,492 (2.31%)	817,135 (1.48%)	613,774 (1.11%)	1,499,543 (2.72%)	415,219 (0.75%)	44,151,244 (79.96%)	55,218,591 (100%)
**Total**	18,939,282 (5.63%)	19,944,634 (5.93%)	8,837,006 (2.63%)	5,171,204 (1.54%)	3,345,307 (0.99%)	8,348,567 (2.48%)	2,239,263 (0.67%)	269,710,000 (80.14%)	336,535,263 (100%)

For peer review only

Fig A1: Breakdown of PCRS reimbursed items by preferred drug status: 2011-2016

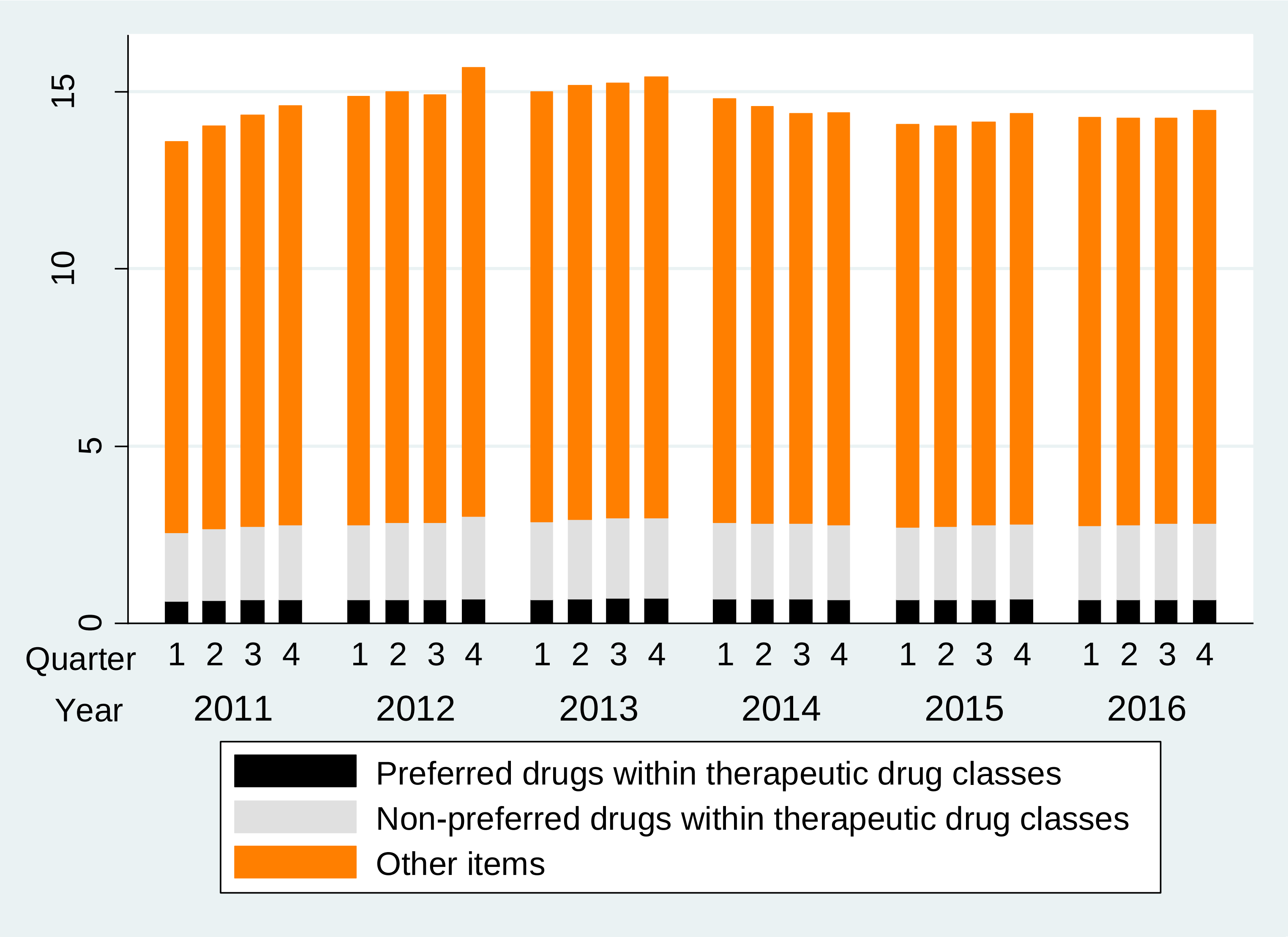

Fig A2: Observed percentage of preferred drugs by drug class

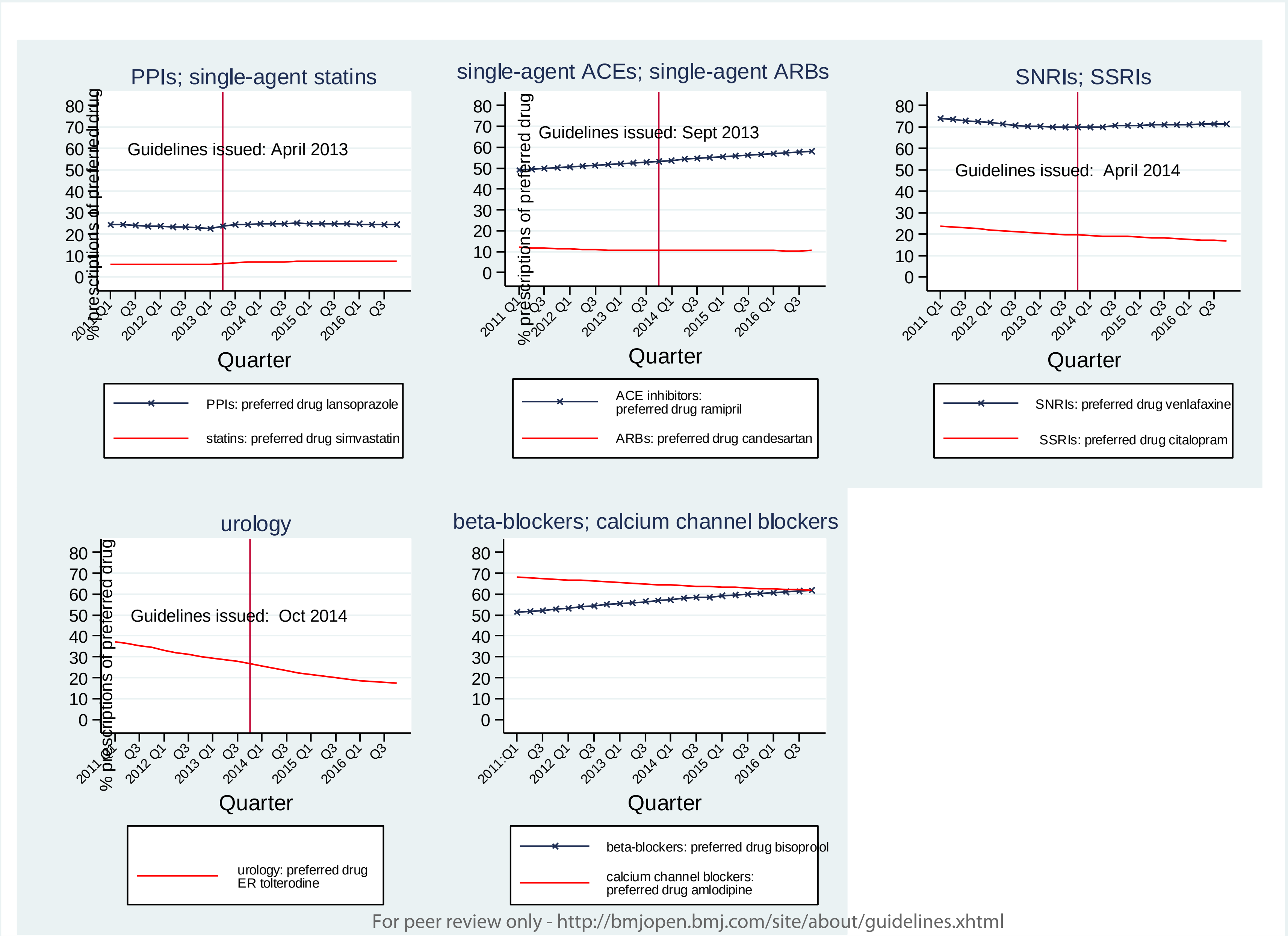

Table A3: Sensitivity analyses for segmented regression models

	Calendar quarters retained for analysis (Study Period)			
	**All available data: 9 quarters before guidelines, 15 quarters after guidelines, (Jan 11- Dec 16)**	**9 quarters before guidelines, 13 quarters after guidelines (Jan 11-Jun 16)**	**9 quarters before guidelines, 11 quarters after guidelines (Jan 11-Dec 15)**	**9 quarters before guidelines, 9 quarters after guidelines (Jan 11-Jun 15)**
**lansoprazole**				
Percentage of preferred drug items: beginning of study period (SE), 95% CI	24.53 (0.47), (23.59,25.47)	24.51 (0.40), (23.66,25.36)	24.47 (0.29), (23.85,25.09)	24.42 (0.19), (24.02,24.83)
Increase in % of preferred drug items per quarter following commencement of study period (SE), 95%CI, p-value	-0.21 (0.05), (-0.32,-0.11), p=0.001	-0.21 (0.05), (-0.32,-0.10), p=0.001	-0.21 (0.04), (-0.30,-0.11), p<0.001	-0.21 (0.04), (-0.28,-0.14), p<0.001
Increase in % of preferred drug items quarter immediately following PDI guidelines, (SE), 95%CI, p-value	1.21 (0.18), (0.84,1.57), p<0.001	1.21 (0.18), (0.83,1.59), p<0.001	1.22 (0.17), (0.85,1.59), p<0.001	1.26 (0.16), (0.90,1.61), p<0.001
Increase in % of preferred drug items per quarter post PDI guidelines, (SE), 95%CI, p-value	0.04 (0.04), (-0.03,0.12), p=0.25	0.06 (0.04), (-0.02,0.14), p=0.14	0.09 (0.04) (0.02,0.18), p=0.01	0.14 (0.03) (0.08,0.22), p<0.001
**simvastatin**				
Percentage of preferred drug items: beginning of study period (SE), 95% CI	5.94 (0.21), (5.50,6.38)	5.92 (0.17), (5.56,6.27)	5.89 (0.12), (5.63,6.15)	5.87 (0.07), (5.73,6.01)
Increase in % of preferred drug items per quarter following commencement of study period (SE), 95%CI, p-value	0.02 (0.03), (-0.04,0.07), p=0.54	0.02 (0.03), (-0.03,0.07), p=0.49	0.02 (0.02), (-0.03,0.07), p=0.32	0.02 (0.02), (-0.02,0.04), p=0.27
Increase in % of preferred drug items quarter immediately following PDI guidelines, (SE), 95%CI, p-value	0.30 (0.10), (0.10,0.50), p=0.01	0.30 (0.10), (0.10,0.51), p=0.01	0.32 (0.10), (0.12,0.52), p=0.01	0.42 (0.09), (0.23,0.62), p<0.001
Increase in % of preferred drug items per quarter post PDI guidelines, (SE), 95%CI, p-value	0.07 (0.02), (0.03,0.10), p=0.002	0.08 (0.02), (0.04,0.12), p=0.001	0.09 (0.02), (0.06,0.13), p<0.001	0.10 (0.01), (0.07,0.13), p<0.001

Table A3 (cont): sensitivity analyses for segmented regression models

	Calendar quarters retained for analysis (Study Period)					
	**All available data: 11 quarters before guidelines, 13 quarters after guidelines (Jan 11-Dec 16)**	**11 quarters before guidelines, 11 quarters after guidelines (Jan 11-Jun 16)**	**11 quarters before guidelines, 9 quarters after guidelines (Jan 11-Dec 15)**	**9 quarters before guidelines, 13 quarters after guidelines (Jul 11-Dec 16)**	**9 quarters before guidelines, 11 quarters after guidelines (Jul 11-Jun 16)**	**9 quarters before guidelines, 9 quarters after guidelines (Jul 11-Jan 15)**
**ramipril**						
Percentage of preferred drug items: beginning of study period (SE), 95% CI	49.14 (0.07), (48.99,49.28)	49.14 (0.08), (48.97,49.30)	49.14 (0.07), (48.98,49.28)	49.93 (0.07), (49.78,50.08)	49.92 (0.07), (49.76,50.10)	49.94 (0.07), (49.78,50.10)
Increase in % of preferred drug items per quarter following commencement of study period (SE), 95%CI, p-value	0.38 (0.01), (0.35,0.40), p<0.001	0.38 (0.02), (0.35,0.40), p<0.001	0.38 (0.02). (0.35,0.40), p<0.001	0.37 (0.01), (0.34,0.40), p<0.001	0.37 (0.01). (0.34,0.40), p<0.001	0.37 (0.01). (0.33,0.40), p<0.001
Increase in % of preferred drug items quarter immediately following PDI guidelines, (SE), 95%CI, p-value	0.16 (0.07), (0.01,0.31), p=0.04	0.16 (0.07), (0.01,0.32), p=0.04	0.16 (0.08), (-0.01,0.33), p=0.04	0.16 (0.07), (0.02,0.34), p=0.03	0.17 (0.08), (0.01,0.35), p=0.04	0.18 (0.09), (-0.01,0.35), p=0.06
Increase in % of preferred drug items per quarter post PDI guidelines, (SE), 95%CI, p-value	0.41 (0.01), (0.39,0.42), p<0.001	0.40 (0.01), (0.38,0.43), p<0.001	0.41 (0.01), (0.38,0.44), p<0.001	0.41 (0.01), (0.39,0.42), p<0.001	0.40 (0.01), (0.38,0.43), p<0.001	0.41 (0.01), (0.38,0.44), p<0.001
**candesartan**						
Percentage of preferred drug items: beginning of study period (SE), 95% CI	11.90 (0.08), (11.73,12.07)	11.90 (0.09), (11.71,12.09)	11.89 (0.08), (11.73,12.06)	11.60 (0.09), (11.42,11.80)	11.61 (0.11), (11.40,11.84)	11.60 (0.08), (11.41,11.78)
Increase in % of preferred drug items per quarter following commencement of study period (SE), 95%CI, p-value	-0.15 (0.01), (-0.17,-0.12), p<0.001	-0.15 (0.01), (-0.17,-0.12), p<0.001	-0.16 (0.01), (-0.17,-0.12), p<0.001	-0.15 (0.01), (-0.17,-0.12), p<0.001	-0.15 (0.01), (-0.18,-0.12), p<0.001	-0.15 (0.01), (-0.18,-0.12), p<0.001
Increase in % of preferred drug items quarter immediately following PDI guidelines, (SE), 95%CI, p-value	0.15 (0.06), (0.02,0.29), p=0.03	0.14 (0.06), (0.01,0.29), p=0.04	0.16 (0.06), (0.02,0.30), p=0.03	0.14 (0.07), (0.01,0.28), p=0.04	0.14 (0.07), (-0.01,0.28), p=0.06	0.15 (0.06), (0.01,0.29), p=0.05
Increase in % of preferred drug items per quarter post PDI guidelines, (SE), 95%CI, p-value	0.01 (0.01), (-0.01,0.03), p=0.46	0.01 (0.01), (-0.02,0.03), p=0.75	0.02 (0.01), (-0.01,0.05), p=0.25	0.01 (0.01), (-0.01,0.03), p=0.49	0.01 (0.01), (-0.03,0.03), p=0.82	0.01 (0.01), (-0.01,0.05), p=0.31

Table A3 (cont): sensitivity analyses for segmented regression models

	**Calendar quarters retained for analysis (Study Period)**					
	**All available data: 13 quarters before guidelines, 11 quarters after guidelines (Jan 11-Dec 16)**	**13 quarters before guidelines, 9 quarters after guidelines (Jan 11-Jun 16)**	**11 quarters before guidelines, 11 quarters after guidelines (Jul 11-Dec 16)**	**11 quarters before guidelines, 9 quarters after guidelines (Jul 11-Jun 16)**	**9 quarters before guidelines, 11 quarters after guidelines (Jan 12-Dec 16)**	**9 quarters before guidelines, 9 quarters after guidelines (Jan 12-Jun 16)**
**venlafaxine**						
Percentage of preferred drug items: beginning of study period (SE), 95% CI	73.61 (0.44), (72.69,74.53)	73.61 (0.46), (72.63,74.60)	72.56 (0.35), (71.81,73.31)	72.57(0.38), (71.75,73.40)	71.45 (0.22), (70.98,71.91)	71.46 (0.24), (70.94,71.98)
Increase in % of preferred drug items per quarter following commencement of study period (SE), 95%CI, p-value	-0.35 (0.05), (-0.46,-0.24), p<0.001	-0.35 (0.05), (-0.46,-0.24), p<0.001	-0.32 (0.05), (-0.43,-0.21), p<0.001	-0.32 (0.05), (-0.43,-0.20), p<0.001	-0.25 (0.04), (-0.34,-0.16), p<0.001	-0.25 (0.05), (-0.35,-0.14), p<0.001
Increase in % of preferred drug items quarter immediately following PDI guidelines, (SE), 95%CI, p-value	0.71 (0.27), (0.15,1.27), p=0.02	0.71 (0.28), (0.12,1.29), p=0.02	0.71 (0.28), (0.12,1.31), p=0.02	0.70 (0.29), (0.08,1.32), p=0.03	0.79 (0.29), (0.16,1.42), p=0.02	0.78 (0.31), (0.10,1.45), p=0.03
Increase in % of preferred drug items per quarter post PDI guidelines, (SE), 95%CI, p-value	0.26 (0.13), (-0.02,0.55), p=0.07	0.26 (0.14), (-0.04,0.55), p=0.08	0.27 (0.13), (-0.01,0.56), p=0.05	0.26 (0.14), (-0.02,0.57), p=0.08	0.26 (0.12), (0.01,0.52), p=0.04	0.26 (0.13), (-0.02,0.53), p=0.07
Increase in % of preferred drug April-June 2015 following introduction of generic duloxetine, (SE), 95%CI, p-value	-0.09 (0.30), (-0.73,0.54), p=0.76	-0.09 (0.31), (-0.76,0.57), p=0.77	-0.11 (0.32), (-0.79,0.57), p=0.74	-0.11 (0.33), (-0.82,0.60), p=0.75	-0.15 (0.35), (-0.89,0.60), p=0.68	-0.17 (0.37), (-0.97,0.64), p=0.66
Increase in % of preferred drug items per quarter post June 2015 , (SE), 95%CI, p-value	-0.08 (0.09), (-0.10,0.26), p=0.34	0.14 (0.12), (-0.10,0.39), p=0.24	-0.08 (0.08), (-0.09,0.26), p=0.34	0.14 (0.11), (-0.10,0.39), p=0.26	0.07 (0.06), (-0.06,0.20), p=0.27	0.10 (0.09), (-0.10,0.33), p=0.27
**citalopram**						
Percentage of preferred drug items: beginning of study period (SE), 95% CI	23.58 (0.13), (23.31,23.85)	23.58 (0.12), (23.32,23.83)	22.88 (0.14), (22.89,23.17)	22.87 (0.13), (22.59,23.14)	21.95 (0.08), (21.78,22.12)	21.93 (0.06), (21.81,22.04)
Increase in % of preferred drug items per quarter following commencement of study period (SE), 95%CI, p-value	-0.36 (0.01), (-0.39,-0.33), p<0.001	-0.36 (0.01), (-0.39,-0.33), p<0.001	-0.36 (0.02), (-0.40,-0.32), p<0.001	-0.36 (0.02), (-0.40,-0.33), p<0.001	-0.33 (0.01), (-0.36,-0.30), p<0.001	-0.33 (0.01), (-0.36,-0.31), p<0.001
Increase in % of preferred drug items quarter immediately following PDI guidelines, (SE), 95%CI, p-value	0.30 (0.08), (0.12,0.47), p=0.002	0.30 (0.08), (0.12,0.48), p=0.003	0.30 (0.09), (0.11,0.48), p=0.003	0.30 (0.09), (0.11,0.50), p=0.005	0.30 (0.08), (0.13,0.47), p=0.002	0.34 (0.08), (0.17,0.51), p=0.001
Increase in % of preferred drug items per quarter post PDI guidelines, (SE), 95%CI, p-value	-0.23 (0.02), (-0.27,-0.19), p<0.001	-0.22 (0.02), (-0.26,-0.18), p<0.001	-0.23 (0.02), (-0.27,-0.19), p<0.001	-0.22 (0.02), (-0.27,-0.17), p<0.001	-0.24 (0.02), (-0.26,-0.21), p<0.001	-0.23 (0.01), (-0.25,-0.20), p<0.001

Table A3 (cont): sensitivity analyses for segmented regression models

	Calendar quarters retained for analysis (Study Period)			
	**All available data: 15 quarters before guidelines, 9 quarters after guidelines (Jan 11-Dec 16)**	**13 quarters before guidelines, 9 quarters after guidelines (Jul 11-Dec 16)**	**11 quarters before guidelines, 9 quarters after guidelines (Jan 12-Dec 16)**	**9 quarters before guidelines, 9 quarters after guidelines (Jul 12-Dec 16)**
**ER tolterodine**				
Percentage of preferred drug items: beginning of study period (SE), 95% CI	37.27 (0.27), (36.69,37.84)	35.45 (0.30), (34.81,36.09)	33.16 (0.33), (32.46,33.87)	31.10 (0.39) (30.29,31.93)
Increase in % of preferred drug items per quarter following commencement of study period (SE), 95%CI, p-value	-1.00 (0.05), (-1.11,-0.88), p<0.001	-1.04 (0.07), (-1.21,-0.88), p<0.001	-0.97 (0.11), (-1.21,-0.73), p<0.001	-0.98 (0.06), (-1.11,-0.86), p<0.001
Increase in % of preferred drug items Jan-Mar 2013 following licensing of mirabegron, (SE), 95%CI, p-value	0.16 (0.24), (-0.35,0.66), p=0.52	0.21 (0.26), (-0.34,0.75), p=0.43	0.11 (0.25), (-0.44,0.65), p=0.68	*
Increase in % of preferred drug items per quarter post March 2013 (SE), 95%CI, p-value	-1.04 (0.06), (-1.17,-0.91), p<0.001	-1.03 (0.07), (-1.17,-0.89), p<0.001	-1.03 (0.06), (-1.17,-0.89), p<0.001	*
Increase in % of preferred drug items quarter immediately following PDI guidelines, (SE), 95%CI, p-value	-0.06 (0.24), (-0.57,0.45), p=0.82	-0.05 (0.25), (-0.57,0.49), p=0.86	-0.01 (0.24), (-0.51,0.49), p=0.96	-0.05 (0.22), (-0.52,0.43), p=0.86
Increase in % of preferred drug items per quarter post PDI guidelines, (SE), 95%CI, p-value	-0.63 (0.09), (-0.73,-0.52), p<0.001	-0.63 (0.06), (-0.73,-0.51), p<0.001	-0.62 (0.05), (-0.73,-0.51), p<0.001	-0.63 (0.06), (-0.75,-0.50), p<0.001

*omitted due to close proximity of study period (July 2012) and licensing of mirabegron (Jan 2013)

Table A4: Sensitivity analyses: alternative definition of calendar quarters for ACE inhibitors/ARBs

	**Calendar quarters: Jan-Mar, Apr-Jun, Jul-Sep, Oct-Dec (24 calendar quarters: Jan 11-Dec 16)**	**Calendar quarters: Mar-May, Jun-Aug, Sep-Nov, Dec-Feb (23 calendar quarters: Mar 11-Nov 16)**
**ramipril**		
Percentage of preferred drug items: beginning of study period (SE), 95% CI	49.14 (0.07), (48.99,49.28)	49.25 (0.07), (49.27, 49.58)
Increase in % of preferred drug items per quarter following commencement of study period (SE), 95%CI, p-value	0.38 (0.01), (0.35,0.40), p<0.001	0.37 (0.01), (0.34,0.40), p<0.001
Increase in % of preferred drug items quarter immediately following PDI guidelines, (SE), 95%CI, p-value	0.16 (0.07), (0.01,0.31), p=0.04	0.14 (0.08), (-0.01,0.30), p=0.05
Increase in % of preferred drug items per quarter post PDI guidelines, (SE), 95%CI, p-value	0.41 (0.01), (0.39,0.42), p<0.001	0.41 (0.01), (0.39,0.43), p<0.001
**candesartan**		
Percentage of preferred drug items: beginning of study period (SE), 95% CI	11.90 (0.08), (11.73,12.07)	11.78 (0.07), (11.63,11.92)
Increase in % of preferred drug items per quarter following commencement of study period (SE), 95%CI, p-value	-0.15 (0.01), (-0.17,-0.12), p<0.001	-0.15 (0.01), (-0.17,-0.13), p<0.001
Increase in % of preferred drug items quarter immediately following PDI guidelines, (SE), 95%CI, p-value	0.15 (0.06), (0.02,0.29), p=0.03	0.17 (0.06), (0.06,0.29), p=0.01
Increase in % of preferred drug items per quarter post PDI guidelines, (SE), 95%CI, p-value	0.01 (0.01), (-0.01,0.03), p=0.46	0.01 (0.01), (-0.02,0.02), p=0.90

For peer review only - http://bmjopen.bmj.com/site/about/guidelines.xhtml

STROBE 2007 (v4) checklist of items to be included in reports of observational studies in epidemiology*
Checklist for cohort, case-control, and cross-sectional studies (combined)

Section/Topic	Item #	Recommendation	Reported on page #
**Title and abstract**	1	(*a*) Indicate the study's design with a commonly used term in the title or the abstract	5
		(*b*) Provide in the abstract an informative and balanced summary of what was done and what was found	2
**Introduction**			
Background/rationale	2	Explain the scientific background and rationale for the investigation being reported	4
Objectives	3	State specific objectives, including any pre-specified hypotheses	4
**Methods**			
Study design	4	Present key elements of study design early in the paper	5
Setting	5	Describe the setting, locations, and relevant dates, including periods of recruitment, exposure, follow-up, and data collection	5
Participants	6	(*a*) *Cohort study*—Give the eligibility criteria, and the sources and methods of selection of participants. Describe methods of follow-up<br>*Case-control study*—Give the eligibility criteria, and the sources and methods of case ascertainment and control selection. Give the rationale for the choice of cases and controls<br>*Cross-sectional study*—Give the eligibility criteria, and the sources and methods of selection of participants	5
		(*b*) *Cohort study*—For matched studies, give matching criteria and number of exposed and unexposed<br>*Case-control study*—For matched studies, give matching criteria and the number of controls per case	
Variables	7	Clearly define all outcomes, exposures, predictors, potential confounders, and effect modifiers. Give diagnostic criteria, if applicable	5
Data sources/ measurement	8*	For each variable of interest, give sources of data and details of methods of assessment (measurement). Describe comparability of assessment methods if there is more than one group	*5*
Bias	9	Describe any efforts to address potential sources of bias	10-all available data used
Study size	10	Explain how the study size was arrived at	5-all available data used
Quantitative variables	11	Explain how quantitative variables were handled in the analyses. If applicable, describe which groupings were chosen and why	5-groupings as per medicine group
Statistical methods	12	(*a*) Describe all statistical methods, including those used to control for confounding	5/6
		(*b*) Describe any methods used to examine subgroups and interactions	n/a

For peer review only

		(*c*) Explain how missing data were addressed	n/a
		(*d*) *Cohort study*—If applicable, explain how loss to follow-up was addressed<br>*Case-control study*—If applicable, explain how matching of cases and controls was addressed<br>*Cross-sectional study*—If applicable, describe analytical methods taking account of sampling strategy	n/a
		(*e*) Describe any sensitivity analyses	6, Appendix
**Results**			
Participants	13*	(a) Report numbers of individuals at each stage of study—eg numbers potentially eligible, examined for eligibility, confirmed eligible, included in the study, completing follow-up, and analysed	7
		(b) Give reasons for non-participation at each stage	n/a
		(c) Consider use of a flow diagram	N/A
Descriptive data	14*	(a) Give characteristics of study participants (eg demographic, clinical, social) and information on exposures and potential confounders	N/A
		(b) Indicate number of participants with missing data for each variable of interest	N/A
		(c) *Cohort study*—Summarise follow-up time (eg, average and total amount)	All data 2011-2016 used
Outcome data	15*	*Cohort study*—Report numbers of outcome events or summary measures over time	N/a
		Case-control study—Report numbers in each exposure category, or summary measures of exposure	N/A
		Cross-sectional study—Report numbers of outcome events or summary measures	7, Table 1
Main results	16	(*a*) Give unadjusted estimates and, if applicable, confounder-adjusted estimates and their precision (eg, 95% confidence interval). Make clear which confounders were adjusted for and why they were included	7,8
		(*b*) Report category boundaries when continuous variables were categorized	n/a
		(*c*) If relevant, consider translating estimates of relative risk into absolute risk for a meaningful time period	8
Other analyses	17	Report other analyses done—eg analyses of subgroups and interactions, and sensitivity analyses	8, Appendix
**Discussion**			
Key results	18	Summarise key results with reference to study objectives	9
Limitations	19	Discuss limitations of the study, taking into account sources of potential bias or imprecision. Discuss both direction and magnitude of any potential bias	10
Interpretation	20	Give a cautious overall interpretation of results considering objectives, limitations, multiplicity of analyses, results from similar studies, and other relevant evidence	9/10
Generalisability	21	Discuss the generalisability (external validity) of the study results	9/10
**Other information**			
Funding	22	Give the source of funding and the role of the funders for the present study and, if applicable, for the original study on which the present article is based	12

For peer review only

*Give information separately for cases and controls in case-control studies and, if applicable, for exposed and unexposed groups in cohort and cross-sectional studies.

Note: An Explanation and Elaboration article discusses each checklist item and gives methodological background and published examples of transparent reporting. The STROBE checklist is best used in conjunction with this article (freely available on the Web sites of PLoS Medicine at http://www.plosmedicine.org/, Annals of Internal Medicine at http://www.annals.org/, and Epidemiology at http://www.epidem.com/). Information on the STROBE Initiative is available at www.strobe-statement.org.

For peer review only
